# Canadian clinical practice guidelines for the management of anxiety, posttraumatic stress and obsessive-compulsive disorders

**DOI:** 10.1186/1471-244X-14-S1-S1

**Published:** 2014-07-02

**Authors:** Martin A Katzman, Pierre Bleau, Pierre Blier, Pratap Chokka, Kevin Kjernisted, Michael Van Ameringen, the Canadian Anxiety Guidelines Initiative Group on behalf of the Anxiety Disorders Association of Canada/Association Canadienne des troubles anxieux and McGill University

**Affiliations:** 1 Department of Psychiatry, University of Toronto, Toronto, ON, M5S 1A1, Canada; 2 Department of Psychiatry, McGill University, Montreal, QC, H3A 1A1, Canada; 3 Department of Psychiatry and Cellular/Molecular Medicines, University of Ottawa, Ottawa, ON, K1Z 7K4, Canada; 4 Department of Psychiatry, University of Alberta, Edmonton, AB, T6G 2R7, Canada; 5 Department of Psychiatry, University of British Columbia, Vancouver, BC, V6T 2A1, Canada; 6 Department of Psychiatry and Behavioural Neuroscience, McMaster University, Hamilton, ON, L8N 3K7, Canada

**Keywords:** guidelines, recommendations, anxiety disorders, panic disorder, agoraphobia, specific phobia, social anxiety disorder, generalized anxiety disorder, GAD, obsessive-compulsive disorder, OCD, posttraumatic stress disorder, PTSD, pharmacotherapy, psychotherapy, special populations

## Abstract

**Background:**

Anxiety and related disorders are among the most common mental disorders, with lifetime prevalence reportedly as high as 31%. Unfortunately, anxiety disorders are under-diagnosed and under-treated.

**Methods:**

These guidelines were developed by Canadian experts in anxiety and related disorders through a consensus process. Data on the epidemiology, diagnosis, and treatment (psychological and pharmacological) were obtained through MEDLINE, PsycINFO, and manual searches (1980–2012). Treatment strategies were rated on strength of evidence, and a clinical recommendation for each intervention was made, based on global impression of efficacy, effectiveness, and side effects, using a modified version of the periodic health examination guidelines.

**Results:**

These guidelines are presented in 10 sections, including an introduction, principles of diagnosis and management, six sections (Sections 3 through 8) on the specific anxiety-related disorders (panic disorder, agoraphobia, specific phobia, social anxiety disorder, generalized anxiety disorder, obsessive-compulsive disorder, and posttraumatic stress disorder), and two additional sections on special populations (children/adolescents, pregnant/lactating women, and the elderly) and clinical issues in patients with comorbid conditions.

**Conclusions:**

Anxiety and related disorders are very common in clinical practice, and frequently comorbid with other psychiatric and medical conditions. Optimal management requires a good understanding of the efficacy and side effect profiles of pharmacological and psychological treatments.

## Introduction

Anxiety and related disorders are among the most common of mental disorders. Lifetime prevalence of anxiety disorders is reportedly as high as 31%; higher than the lifetime prevalence of mood disorders and substance use disorders (SUDs) [1-5]. Unfortunately, anxiety disorders are under-diagnosed [6] and under-treated [5,7,8].

These guidelines were developed to assist clinicians, including primary care physicians and psychiatrists, as well as psychologists, social workers, occupational therapists, and nurses with the diagnosis and treatment of anxiety and related disorders by providing practical, evidence-based recommendations. This guideline document is not focused on any individual type of clinician but rather on assessing the data and making recommendations. Subsequent “user friendly” tools and other initiatives are planned.

The guidelines include panic disorder, agoraphobia, specific phobia, social anxiety disorder (SAD), generalized anxiety disorder (GAD), as well as obsessive-compulsive disorder (OCD), and posttraumatic stress disorder (PTSD). Also included are brief discussions of clinically relevant issues in the management of anxiety and related disorders in children and adolescents, women who are pregnant or lactating, and elderly patients, and patients with comorbid conditions.

### Methods

These guidelines are based on a thorough review of the current literature and were developed by a panel of Canadian experts in anxiety and related disorders through a consensus process. Data on the epidemiology, diagnosis, and treatment (psychological and pharmacological) were obtained through MEDLINE searches of English language citations (1980–2012), using search terms encompassing the specific treatments and specific anxiety and related disorders. These searches were supplemented with data from PsycINFO and manual searches of the bibliographies of efficacy studies, meta-analyses, and review articles. Treatment strategies were rated on strength of evidence for the intervention (Table 1). A clinical recommendation for each intervention was then made, based on global impression of efficacy in clinical trials, effectiveness in clinical practice, and side effects, using a modified version of the periodic health examination guidelines (Table 2).

**Table 1:** Levels of evidence

1	Meta-analysis or at least 2 randomized controlled trials (RCTs) that included a placebo condition
**2**	At least 1 RCT with placebo or active comparison condition

**3**	Uncontrolled trial with at least 10 subjects

**4**	Anecdotal reports or expert opinion

Levels of evidence do not assume positive or negative or equivocal results, they merely represent the quality and nature of the studies that have been conducted.

Level 1 and Level 2 evidence refer to treatment studies in which randomized comparisons are available. Recommendations involving epidemiological or risk factors primarily arise from observational studies, hence the highest level of evidence for these is usually Level 3. Recommendations, such as principles of care, reflect consensus opinion based on evidence from various data sources, and therefore are primarily Level 4 evidence.

**Table 2:** Treatment recommendation summary

**First-line**	Level 1 or Level 2 evidence plus clinical support for efficacy and safety
**Second-line**	Level 3 evidence or higher plus clinical support for efficacy and safety
**Third-line**	Level 4 evidence or higher plus clinical support for efficacy and safety
**Not recommended**	Level 1 or Level 2 evidence for lack of efficacy

The guidelines were initiated prior to the introduction of the American Psychiatric Association’s (APA) fifth edition of the Diagnostic and Statistical Manual of Mental Disorders (DSM-5) and the committee was sensitive to potential changes to the nosology of anxiety and related disorders and its impact on the guidelines. However, it was agreed that, since the evidence for treatment is based on studies using DSM-IV criteria (or earlier), the introduction of the DSM-5 would not fundamentally alter the evidence and recommendations at this time. Whether using DSM-5 diagnostic criteria for the inclusion patients in clinical trials in the future will have an impact on outcomes, remains to be seen.

The panel of Canadian experts in anxiety and related disorders responsible for the development of these guidelines via consensus process included 10 psychiatrists and seven psychologists who were organized into subpanels based on their expertise in particular anxiety or related disorders as well as in treating specific patient populations. Preliminary treatment recommendations and the evidence upon which they had been based were reviewed at a meeting of the panel in December 2012; subsequently, draft guidelines were prepared by the subpanels which were then circulated to the entire group for consensus ratification during 2013. Preliminary recommendations were also presented to the Canadian psychiatric community for input in September 2012 at the Canadian Psychiatric Association annual conference.

These guidelines are presented in 10 sections, the first of which is this introduction. In the following section, the principles of diagnosis and management of anxiety and related disorders are covered. That section provides an overview of the differential diagnoses associated with anxiety and related disorders in general, discusses issues that affect all anxiety disorders, and presents the general advantages and disadvantages of psychological treatment and pharmacotherapy options. In the subsequent six sections (Sections 3 through 8), the specific diagnosis and management of the individual anxiety and related disorders (panic disorder, specific phobia, SAD, OCD, GAD, and PTSD) are reviewed and recommendations are made for psychological and pharmacological treatments. Section 9 discusses issues that may warrant special attention pertaining to anxiety and related disorders in children and adolescents, pregnant or lactating women, and the elderly. The last section of these guidelines addresses clinical issues that may arise when treating patients with anxiety and related disorders who are also diagnosed with comorbid psychiatric conditions such as major depressive disorder (MDD), bipolar disorder, or other psychoses, and attention deficit/hyperactivity disorder (ADHD), or medical comorbidities, such as pain syndromes, cardiovascular disease, and diabetes/metabolic syndrome.

## Principles of diagnosis and management of anxiety and related disorders

### Epidemiology

#### Prevalence and impact

Anxiety and related disorders are among the most common mental disorders, with lifetime prevalence rates as high as 31% [1-5] and 12-month prevalence rates of about 18% [3,4]. Rates for individual disorders vary widely. Women generally have higher prevalence rates for most anxiety disorders, compared with men [4,5,9]. Anxiety and related disorders are associated with an increased risk of developing a comorbid major depressive disorder [10-12].

Anxiety and related disorders put a significant burden on patients and their family members [13]. They are associated with substantial functional impairment, which increases as the severity of anxiety [14] or the number of comorbid anxiety disorders increases [7,15]. In addition, studies have demonstrated quality of life impairments in patients with various anxiety and related disorders [16,17]. Anxiety has a considerable economic impact on society as well, being associated with greater use of health care services [5,18] and decreased work productivity [18,19].

Importantly, studies report that about 40% of patients diagnosed with anxiety and related disorder are untreated [5,7].

#### Suicide risk

In large surveys, anxiety and related disorders were independently associated with a significant 1.7-2.5 times increased risk of suicide attempts [20-23]; however, data are conflicting as to whether the risk is moderated by gender [20,23]. Increased risk of suicide attempts or completed suicide has been reported for patients with panic disorder, PTSD [20,24], and GAD [24], even in the absence of a comorbid mood disorder. These data indicate that patients with an anxiety disorder warrant explicit evaluation for suicide risk. The presence of a comorbid mood disorder significantly increases the risk of suicidal behavior [22,25].

### Initial assessment of patients with anxiety

The management of patients presenting with anxiety symptoms should initially follow the flow of the five main components outlined in Table 3.

**Table 3:** Overview of the management of anxiety and related disorders

• Screen for anxiety and related symptoms • Conduct differential diagnosis (consider severity, impairment, and comorbidity) • Identify specific anxiety or related disorder • Psychological and/or pharmacological treatment • Perform follow-up

#### Screen for anxiety and related symptoms

Anxiety and related disorders are generally characterized by the features of excessive anxiety, fear, worry, and avoidance. While anxiety can be a normal part of everyday life, anxiety disorders are associated with functional impairment; as part of the key diagnostic criteria for anxiety disorders is the requirement that the symptoms cause clinically significant distress or impairment in social, occupational, or other important areas of functioning [26].

Asking patients if they are feeling nervous, anxious or on edge, or whether they have uncontrollable worry, can be useful to detect anxiety in patients in whom the clinician suspects an anxiety or related disorder [7]. The DSM-5 suggests the questions shown in Table 4 for the identification of anxiety-related symptoms; items scored as mild or greater may warrant further assessment [26]. If anxiety symptoms are endorsed, they should be explored in more detail by including questions about the onset of the anxiety symptoms, associations with life events or trauma, the nature of the anxiety (i.e., worry, avoidance, or obsession), and the impact they have had on the patient’s current functioning.

**Table 4:** General screening questions

• During the past two weeks how much have you been bothered by the following problems? ○ Feeling nervous, anxious, frightened, worried, or on edge ○ Feeling panic or being frightened ○ Avoiding situations that make you anxious

Adapted from reference [26].

Table 5 presents suggested screening questions for individual anxiety and related disorders, from various validated screening tools [27-30], some of which are freely available online (e.g., http://www.macanxiety.com/online-anxiety-screening-test).

**Table 5:** Screening questions for specific anxiety and related disorders

**Panic disorder – MACSCREEN **[29,30] • Do you have sudden episodes/spells/attacks of intense fear or discomfort that are unexpected or out of the blue? If you answered "YES" then continue • Have you had more than one of these attacks? • Does the worst part of these attacks usually peak within several minutes? • Have you ever had one of these attacks and spent the next month or more living in fear of having another attack or worrying about the consequences of the attack?
**SAD** (Based on Mini-SPIN [28]) • Does fear of embarrassment cause you to avoid doing things or speaking to people? • Do you avoid activities in which you are the center of attention? • Is being embarrassed or looking stupid among your worst fears?

**GAD **[31] • During the past 4 weeks, have you been bothered by feeling worried, tense, or anxious most of the time? • Are you frequently tense, irritable, and having trouble sleeping?

**OCD – MACSCREEN **[29,30] **Obsessions:** • Are you bothered by repeated and unwanted thoughts of any of the following types: ○ Thoughts of hurting someone else ○ Sexual thoughts ○ Excessive concern about contamination/germs/disease ○ Preoccupation with doubts (“what if” questions) or an inability to make decisions ○ Mental rituals (e.g., counting, praying, repeating) ○ Other unwanted intrusive thoughts • If you answered "YES" to any of the above… Do you have trouble resisting these thoughts, images, or impulses when they come into your mind? **Compulsions:** • Do you feel driven to perform certain actions or habits over and over again, or in a certain way, or until it feels just right? Such as: ○ Washing, cleaning ○ Checking (e.g., doors, locks, appliances) ○ Ordering/arranging ○ Repeating (e.g., counting, touching, praying) ○ Hoarding/collecting/saving • If you answered "YES" to any of the above… Do you have trouble resisting the urge to do these things?

**PTSD – MACSCREEN **[29,30] • Have you experienced or seen a life-threatening or traumatic event such as a rape, accident, someone badly hurt or killed, assault, natural or man-made disaster, war, or torture? **If you answered "YES" then continue** • Do you re-experience the event in disturbing (upsetting) ways such as dreams, intrusive memories, flashbacks, or physical reactions to situations that remind you of the event?

#### Conduct differential diagnosis

The differential diagnosis of anxiety and related disorders should consider whether the anxiety is due to another medical or psychiatric condition, is comorbid with another medical or psychiatric condition, or is medication-induced or drug-related [32].

When a patient presents with excessive or uncontrollable anxiety it is important to identify other potential causes of the symptoms, including direct effects of a substance (e.g., drug abuse or medication) or medical condition (e.g., hyperthyroidism, cardiopulmonary disorders, traumatic brain injury), or another mental disorder [26]. However, since comorbid conditions are common, the presence of some of these other conditions may not preclude the diagnosis of an anxiety or related disorder.

Certain risk factors have been associated with anxiety and related disorders and should increase the clinician’s index of suspicion (Table 6) [4,9,33-37]. A family [33] or personal history of mood or anxiety disorders [34,35] is an important predictor of anxiety symptoms. In addition, family history is associated with a more recurrent course, greater impairment, and greater service use [33]. A personal history of stressful life events is also associated the development of anxiety and related disorders [36,37], in particular, childhood abuse [37].

**Table 6:** Common risk factors in patients with anxiety and related disorders

• Family history of anxiety [33] • Personal history of anxiety or mood disorder [34,35] • Childhood stressful life events or trauma [36,37] • Being female [4,9] • Chronic medical illness [34,40] • Behavioral inhibition [41,42]

Women generally have higher prevalence rates across all anxiety and related disorders, compared with men [4,5,9]. The median of age of onset is very early for some phobias and for separation anxiety disorder (seven to 14 years), but later for GAD, panic disorder, and PTSD (24-50 years) [1,2].

Loneliness [38], low education [38], and adverse parenting [39], as well as chronic somatic illnesses, such as cardiovascular disease, diabetes, asthma, and obesity may increase the risk for a lifetime diagnosis of anxiety [34,40].

##### Comorbid medical and psychiatric disorders

Anxiety and related disorders frequently co-occur with other psychiatric disorders [3]. More than half of patients with an anxiety disorder have multiple anxiety disorders [3,15], and almost 30% will have three or more comorbid anxiety or related disorders [3]. Anxiety is often comorbid with substance use and mood disorders [3,40]. An estimated 52% of patients with bipolar disorder [43], 60% of patients with MDD [44], and 47% of those with ADHD [45] will have a comorbid anxiety or related disorder. Therefore, anxiety disorders should be considered in these patients.

The high frequency of comorbidity must be considered when diagnosing anxiety and related disorders since this can have important implications for diagnosis and treatment [32]. Anxiety disorders comorbid with other anxiety or depressive disorders are associated with poorer treatment outcomes, greater severity and chronicity [46-49], more impaired functioning [46], increased health service use [50], and higher treatment costs [51]. The impact tends to increase with an increasing number of comorbid conditions [46].

Patients with anxiety disorders have a higher prevalence of hypertension and other cardiovascular conditions, gastrointestinal disease, arthritis, thyroid disease, respiratory disease, migraine headaches, and allergic conditions compared to those without anxiety disorders [16,52]. Comorbid anxiety and related disorders have a significant impact on quality of life (QoL) in patients with medical conditions [52].

##### Baseline assessment

Baseline assessment should include a review of systems, prescribed medications, over-the-counter agents, alcohol use, caffeine intake, and illicit drug use, in addition to evaluation of the anxiety symptoms and functioning [32]. Table 7 lists potential investigations that can be considered based on an individual patient’s presentation and specific symptoms (e.g., dizziness or tachycardia). Ideally, a physical examination and baseline laboratory investigations should be performed before pharmacotherapy is initiated, with repeat assessments according to best practice guidelines [32]. Patients with anxiety and related disorders should be monitored initially every one to two weeks and then every four weeks for weight changes and adverse effects of medications, as this is a major factor contributing to discontinuation of medication.

**Table 7:** Considerations for baseline laboratory investigations (as needed based on patient’s presenting symptoms)

**Basic lab tests**	
• Complete blood count	• Fasting glucose
• Fasting lipid profile (TC, vLDL, LDL, HDL, TG)	• Thyroid-stimulating hormone
• Electrolytes	• Liver enzymes
**If warranted**	
• Urine toxicology for substance use	

Adapted from references [32,53]. HDL = high density lipoprotein; LDL = low density lipoprotein; TC = total cholesterol; TG = triglyceride; vLDL = very low density lipoprotein.

Closer monitoring may be required in children younger than 10 years of age, older or medically ill patients, patients on medications associated with metabolic changes, and those on multiple medications [32].

#### Identify specific anxiety or related disorder

The fifth edition of the Diagnostic and Statistical Manual of Mental Disorders (DSM-5) has been finalized by the American Psychiatric Association (APA) [26]. The new DSM-5 provides diagnostic criteria for psychiatric disorders based on scientific reviews of the literature, field trial data, internal evaluations, public comments, and a final review by APA’s Board of Trustees.

The “anxiety disorders” chapter now includes panic disorder, agoraphobia, GAD, selective mutism, separation anxiety disorder, SAD (social phobia), specific phobia, substance/medication-induced anxiety disorder, as well as anxiety disorder due to another medical condition or not elsewhere classified. OCD and PTSD have been moved to separate chapters on obsessive-compulsive and related disorders and trauma- and stressor-related disorders, respectively [26].

Table 8 provides a brief summary of the key DSM-5 diagnostic features of the anxiety and related disorders that are included in these guidelines [26]. While the DSM-5 is the most up-to-date diagnostic criteria, it is important to note that the evidence for treatment is based on studies using DSM-IV criteria (or earlier) for inclusion of patients. However, most of the diagnostic criteria have not changed substantially (see Sections 3–9 for more information on diagnosis); the exception being agoraphobia, which is now designated as a separate diagnosis.

**Table 8:** Key features of specific anxiety and related disorders

Disorder	Key features
Panic disorder	• Recurrent unexpected panic attacks, in the absence of triggers • Persistent concern about additional panic attacks and/or maladaptive change in behavior related to the attacks

Agoraphobia	• Marked, unreasonable fear or anxiety about a situation • Active avoidance of feared situation due to thoughts that escape might be difficult or help unavailable if panic-like symptoms occur

Specific phobia	• Marked, unreasonable fear or anxiety about a specific object or situation, which is actively avoided (e.g., flying, heights, animals, receiving an injection, seeing blood)

Social anxiety disorder (SAD)	• Marked, excessive or unrealistic fear or anxiety about social situations in which there is possible exposure to scrutiny by others • Active avoidance of feared situation

Generalized anxiety disorder (GAD)	• Excessive, difficult to control anxiety and worry (apprehensive expectation) about multiple events or activities (e.g., school/work difficulties) • Accompanied by symptoms such as restlessness/feeling on edge or muscle tension

Obsessive–compulsive disorder (OCD)	• Obsessions: recurrent and persistent thoughts, urges, or images that are experienced as intrusive and unwanted and that cause marked anxiety or distress • Compulsions: repetitive behaviors (e.g., hand washing) or mental acts (e.g., counting) that the individual feels driven to perform to reduce the anxiety generated by the obsessions

Posttraumatic stress disorder (PTSD)	• Exposure to actual or threatened death, serious injury, or sexual violation • Intrusion symptoms (e.g., distressing memories or dreams, flashbacks, intense distress) and avoidance of stimuli associated with the event • Negative alterations in cognitions and mood (e.g., negative beliefs and emotions, detachment), as well as marked alterations in arousal and reactivity (e.g., irritable behavior, hypervigilance)

Adapted from reference [26].

Specific individual anxiety and related disorders should be diagnosed with the DSM-5 criteria in the sections devoted to each anxiety disorder. An accurate diagnosis is important to help guide treatment.

#### Psychological and pharmacological treatment

Treatment options for anxiety and related disorders include psychological and pharmacological treatments. All patients should receive education about their disorder, efficacy (including expected time to onset of therapeutic effects) and tolerability of treatment choices, aggravating factors, and signs of relapse [32]. Information on self-help materials such as books or websites may also be helpful.

The choice of psychological or pharmacological treatment depends on factors such as patient preference and motivation, ability of the patient to engage in the treatment, severity of illness, clinicians’ skills and experience, availability of psychological treatments, patient’s prior response to treatment, and the presence of comorbid medical or psychiatric disorders [32].

A brief overview of psychological and pharmacological treatments is provided below, with more specific recommendations in the individual sections for each anxiety and related disorder.

##### Overview of psychological treatment

Psychological treatments play an important role in the management of anxiety and related disorders. Regardless of whether formal psychological treatment is undertaken, patients should receive education and be encouraged to face their fears. Meta-analyses have demonstrated the efficacy of psychological treatments in group and individual formats in patients with panic disorder [54-56], specific phobia [57], SAD [58,59], OCD [60-63], GAD [55,64,65], or PTSD [66-69], particularly exposure-based and other cognitive behavioral therapy (CBT) protocols [70,71], as well as mindfulness-based cognitive therapy (MBCT) [72]. When choosing psychological treatments for individual patients, the forms of therapy that have been most thoroughly evaluated in the particular anxiety or related disorder should be used first.

CBT is not a single approach to treatment, but rather a process that focuses on addressing the factors that caused and maintain the individual patient’s anxiety symptoms [73]. Some of the core components of CBT are shown in Table 9 [73].

**Table 9:** Components of cognitive behavioral interventions

Exposure	• Encourage patients to face fears • Patients learn corrective information through experience • Extinction of fear occurs through repeated exposure • Successful coping enhances self-efficacy
**Safety response inhibition**	• Patients restrict their usual anxiety-reducing behaviors (e.g., escape, need for reassurance) • Decreases negative reinforcement • Coping with anxiety without using anxiety-reducing behavior enhances self-efficacy

**Cognitive strategies**	• Cognitive restructuring, behavioral experiments, and related strategies target patients’ exaggerated perception of danger (e.g., fear of negative evaluation in SAD) • Provides corrective information regarding the level of threat • Can also target self-efficacy beliefs

**Arousal management**	• Relaxation and breathing control skills can help patient control increased anxiety levels

**Surrender of safety signals**	• Patient relinquishes safety signals (e.g., presence of a companion, knowledge of the location of the nearest toilet) • Patients learn adaptive self-efficacy beliefs

Adapted from reference [73].

CBT can be effectively delivered as individual or group therapy for most anxiety and related disorders. In addition, a variety of self-directed or minimal intervention formats (e.g., bibliotherapy/self-help books, or internet/computer-based programs with or without minimal therapist contact) have demonstrated significant improvements in anxiety symptoms [74-79]. Meta-analyses have also shown that exposure therapy can be effectively administered in a virtual reality format [80,81]. These strategies may be particularly useful in cases where real-life exposure is difficult due to inconvenience, expense, or patient reluctance.

Psychotherapy and pharmacotherapy generally demonstrate about equivalent efficacy for the treatment of most anxiety and related disorders [71,82]. Results with combination therapy vary for the different anxiety disorders, and results have been conflicting [82,83] (see Sections 3–9 for evidence and references regarding combination therapy). Therefore, current evidence does not support the routine combination of CBT and pharmacotherapy as initial treatment. However, when patients do not benefit from CBT or have a limited response, a trial of pharmacotherapy is advisable. Similarly, patients who show limited benefit from pharmacotherapy may benefit from CBT. All patients being treated with pharmacotherapy should be instructed to gradually face their fears (exposure to decrease avoidance).

##### Overview of pharmacological treatment

This section provides a general overview of some of the commonly recommended pharmacological agents. Evidence and recommendations for specific medications are described in the individual sections for each of the anxiety and related disorders.

Table 10 shows medications that have Health Canada approved indications for use in different anxiety and related disorders [84], and dosing suggestions are shown in Additional file 1. Various antidepressants including selective serotonin reuptake inhibitors (SSRIs), serotonin norepinephrine reuptake inhibitors (SNRIs), noradrenergic and specific serotonergic antidepressants (NaSSAs), tricyclic antidepressants (TCAs), monoamine oxidase inhibitors (MAOIs), and reversible inhibitors of monoamine oxidase A (RIMAs) have demonstrated some efficacy in the treatment of anxiety and related disorders (see Sections 3–9 for evidence and references). SSRIs and SNRIs are usually preferred as initial treatments, since they are generally safer and better tolerated than TCAs or MAOIs [32].

**Table 10:** Medications with Health Canada–approved indications for anxiety and related disorders

	Anxiety disorders	Panic disorder	Social anxiety disorder	Obsessive–compulsive disorder	Generalized anxiety disorder	Posttraumatic stress disorder
**ANTIDEPRESSANTS**						

**SSRIs**						

Escitalopram (Cipralex^®^)				X	X	

Fluoxetine (Prozac^®^)				X		

Fluvoxamine (Luvox^®^)				X		

Paroxetine (Paxil^®^)		X	X	X	X	X

Paroxetine CR (Paxil^®^ CR)		X	X			

Sertraline (Zoloft^®^)		X		X		

**TCAs**						

Clomipramine				X		

**Other antidepressants**						

Venlafaxine XR (Effexor^®^ XR)		X	X		X	

Duloxetine (Cymbalta^®^)					X	

**AZAPIRONES**						

Buspirone (BuSpar^®^, Buspirex^®^)					X	

BENZODIAZEPINES*	X					

Data from respective Canadian product monographs [84].

*Multiple generic and brand name products, consult product monographs: alprazolam, bromazepam, chlordiazepoxide, clorazepate, diazepam, lorazepam, and oxazepam are indicated for anxiety disorders; alprazolam is also indicated for panic disorder.

CR = controlled release; SSRI = selective serotonin reuptake inhibitor; TCA = tricyclic antidepressant; XR = extended release.

Benzodiazepines may be useful as adjunctive therapy early in treatment, particularly for acute anxiety or agitation, to help patients in times of acute crises, or while waiting for onset of adequate efficacy of SSRIs or other antidepressants [32]. Due to concerns about possible dependency, sedation, cognitive impairment, and other side effects, benzodiazepines should usually be restricted to short-term use, and generally dosed regularly rather than as-needed [32].

Several anticonvulsants and atypical antipsychotics have demonstrated efficacy in some anxiety and related disorders, but for various reasons, including side effects, as well as limited randomized controlled trial (RCT) data and clinical experience, these agents are generally recommended as second-line, third-line, or adjunctive therapies (see Sections 3–9 for evidence and references).

The choice of medication should take into consideration the evidence for its efficacy and safety/tolerability for the treatment of the specific anxiety and related disorder, as well as for any comorbid conditions the patient might have, in both acute and long-term use.

##### Safety and side effects

*Antidepressants:* The most common side effects seen with SSRIs and SNRIs include headache, irritability, gastrointestinal complaints, insomnia, sexual dysfunction, weight gain, increased anxiety, drowsiness, and tremor [85-88]. Patients report that the most common bothersome side effects are sexual dysfunction, drowsiness, fatigue, and weight gain [87,88]. Most side effects occur early and transiently during the first two weeks of treatment, but others, such as sexual dysfunction and weight gain, may persist for the duration of treatment [85,87,89].

Use of SSRIs or SNRIs has been associated with an increased risk of upper gastrointestinal bleeding, particularly when used in combination with nonsteroidal anti-inflammatory drugs (NSAIDs) [90,91]. SSRI use has also been associated with low bone mineral density [92,93], as well as an increased risk of fractures [94] and hyponatremia [95].

Abrupt discontinuation of SSRIs or SNRIs can lead to a discontinuation syndrome with gastrointestinal, psychiatric, vasomotor, and other symptoms [85,96].

Health Canada and the US Food and Drug Administration (FDA) require antidepressants to include a warning regarding an increased risk of suicidal ideation and behavior in children and adolescents [97,98]. The increased risk of suicidal behavior reported in pediatric patients [99] does not appear to be seen in adults, and may in fact be decreased [99,100]. Careful monitoring for evidence of self-harming or suicidal thoughts or behaviors is important in both adult and pediatric patients.

SSRIs and SNRIs are generally better tolerated and safer than TCAs and MAOIs, having less anticholinergic effects, toxicity, lethality, and psychomotor or cognitive impairment [85,101]. MAOIs are generally reserved for second- or third-line treatment because of side effects, drug interactions, and dietary restrictions [32].

*Anxiolytics*: The most common side effects associated with benzodiazepines include primarily sedation, fatigue, ataxia, slurred speech, memory impairment, and weakness [85]. Benzodiazepines are associated with withdrawal reactions, rebound, and dependence, with the risk being greater with short- and intermediate-acting compared to long-acting agents [102]. These agents should be used with caution in patients with SUDs [85,103]. Older patients (generally over 65 years of age) may be at high risk for falls and fractures due to psychomotor impairment associated with benzodiazepines [104,105]. Cognitive impairment has been reported [106], some of which may persist after cessation of therapy [107]. In particular, memory impairment has been associated with high-dose or high-potency benzodiazepines, particularly in older people [102,107].

Reported side effects of azapirones (buspirone) include dizziness, drowsiness, and nausea [32,108].

*Atypical antipsychotics*: Atypical antipsychotics are associated to varying degrees with weight gain, diabetes, and other metabolic side effects, including alterations in glucose and lipid levels [109-116]. Metabolic disturbances generally appear to be higher with olanzapine, intermediate with risperidone and quetiapine, and lower with aripiprazole, asenapine, lurasidone, and ziprasidone [109-114].

Atypical antipsychotics have varying sedative effects, with quetiapine, clozapine, asenapine, and olanzapine generally causing more sedation than ziprasidone, risperidone, lurasidone, or aripiprazole [111,115]. Data on cognitive effects are conflicting, with some studies suggesting improvements [111], while other data suggest greater cognitive dysfunction in patients using, versus those not using, antipsychotics [117].

Because of the risks of diabetes and weight gain, and the fact that there is limited RCT evidence of the efficacy of these agents in anxiety and related disorders, atypical antipsychotics are generally recommended as second-line, third-line, or adjunctive therapies (see Sections 3–9 for evidence and references).

*Anticonvulsants*: Anticonvulsants are associated with gastrointestinal side effects, somnolence, weight gain, tremor, as well as dermatologic and hematologic side effects [111,118]. In addition, several anticonvulsants have a potential risk of serious rash, erythema multiforme, Stevens-Johnson syndrome, or toxic epidermal necrolysis [111]. Regular monitoring of serum medication levels and liver function is required for patients on divalproex [84,111].

#### Follow-up

Anxiety and related disorders are often chronic and a systematic approach to treatment should include patient education, assessment of comorbidities, and evidence-based pharmacological and psychological interventions with adequate monitoring and duration. Pharmacological treatment is often associated with a delay of about two to eight weeks in onset of symptom relief, with full response taking up to 12 weeks or more. Longer-term therapy has been associated with continued symptomatic improvement and the prevention of relapse, and therapy should be continued for at least 12-24 months for most patients [32].

Medication should be initiated at low doses and titrated to the recommended dosage range at one- to two-week intervals over four to six weeks. Once the therapeutic range has been achieved, improvement is usually seen over the next four to eight weeks. Follow-up should occur at two-week intervals for the first six weeks and monthly thereafter [32].

For a patient undergoing psychotherapy, the treatment schedule is structured around weekly contact with a therapist for about 12-20 weeks, although shorter protocols and minimal intervention programs have also proven effective (see Sections 3–9 for evidence and references). A follow-up appointment four weeks later and then every two to three months is usually sufficient [32].

##### Assessing response to treatment

Therapy should seek to improve symptoms and distress. The optimal goal is full remission of symptoms and return to a premorbid level of functioning [32,85]. However, goals may need to be individualized for some patients with disorders that have been present since childhood as they may never have had adequate premorbid functioning. A response to therapy is often defined as a percentage reduction in symptoms (usually 25-50%) on an appropriate scale. Remission is often defined as loss of diagnostic status, a pre-specified low score on an appropriate disorder-specific scale, and no functional impairment in fully recovered patients as measured by a scale such as the Sheehan Disability Scale or SF-36 [32,119,120].

Objective scales can be used to help assess a patient’s progress. The Clinical Global Impression (CGI) scale is brief, comprehensive, and can easily be used at each appointment to assess improvement. The clinician-rated Hamilton Anxiety Rating Scale (HARS) can assess anxiety symptoms in general and is often used in clinical trials but is less practical in clinical practice. A variety of self-report and clinician-rated scales are available to assess the specific anxiety or related disorder.

## Panic disorder and agoraphobia

### Epidemiology

The lifetime and 12-month prevalence of panic disorder have been estimated at 4.7-5.1% and 2.1-2.8%, respectively [121,122]. The estimated prevalence of panic attacks is considerably greater at 28.3% (lifetime) and 6.4-11.2% (12-month) [121,123]. Youth with panic attacks (which often do not meet diagnostic criteria for panic disorder) will frequently have or develop other psychiatric disorders including mood disorders (bipolar disorder and MDD), other anxiety or related disorders, SUDs, eating disorders, psychotic disorders, and personality disorders [122,124,125]. Annually, 8-10% of the general public will have a panic attack without ever developing any identifiable psychopathology [126]. About 40-70% of patients with panic disorder experience nocturnal panic (waking from sleep in a state of panic) [127]. Rates of 12-month and lifetime agoraphobia (without panic) are quite low, at 0.8% and 1.4%, respectively [2,3].

The risk of panic disorder and agoraphobia is higher in women than men, and patients who are middle-aged, widowed/divorced, and those of low income [122]. In the Canadian Community Health Survey 1.2 (CCHS 1.2) there were no differences in the rates of panic disorder or agoraphobia in urban versus rural settings [128].

Panic disorder has a negative impact on both psychological and physical functioning, and puts a substantial burden on the patient’s family [13]. Patients with panic disorder have more QoL impairment and dissatisfaction [16,17], greater likelihood of suicide attempts [20], and increased cognitive and emotional dysfunction [129-133] compared to healthy controls. Panic disorder is also associated with substantial societal costs [134], both in terms of health care utilization [135] and loss of workplace productivity [136]. In a 2012 survey, panic disorder conferred a substantial rate of work absenteeism (mean: 36.0 days/year) [136].

#### Comorbidity

Patients with panic disorder, or those experiencing panic attacks, have significantly increased odds of being diagnosed with a comorbid disorder, including another anxiety or related disorder, mood disorder, impulse-control disorder, or SUD [121,137]. MDD is very common, occurring in an estimated 35-40% of patients with panic disorder [121]. Panic disorder also frequently co-occurs with agoraphobia [138].

Panic disorder is more prevalent in patients with medical conditions, including thyroid disease, cancer, chronic pain, cardiac disease, irritable bowel syndrome, migraine, as well as allergic and respiratory diseases compared with the general population [85,139-141]. The presence of medical comorbidity is associated with greater severity of panic disorder symptoms and disability [140,142].

### Diagnosis

For a diagnosis of panic disorder, a patient must have had recurrent, unexpected panic attacks (Table 11), followed by at least one month of persistent concern or worry about further attacks or their consequences, or a significant maladaptive behavioral change related to attacks (Table 12) [26].

**Table 11:** DSM-5 criteria for panic attacks

• An abrupt surge of intense fear or intense discomfort that reaches a peak within minutes, and includes ≥4 of the following symptoms: (1) Palpitations, pounding heart, or accelerated heart rate (2) Sweating (3) Trembling or shaking (4) Sensations of shortness of breath or smothering (5) Feelings of choking (6) Chest pain or discomfort (7) Nausea or abdominal distress (8) Feeling dizzy, unsteady, light-headed, or faint (9) Chills or heat sensations (10) Paresthesias (numbness or tingling sensations) (11) Derealization (feelings of unreality) or depersonalization (being detached from oneself) (12) Fear of losing control or going crazy (13) Fear of dying

Adapted from reference [26].

**Table 12:** DSM-5 diagnosis of panic disorder

• The person has experienced both of the following: ○ Recurrent unexpected panic attacks ○ ≥1 of the attacks followed by ≥1 month of 1 or both of the following: • Persistent concern or worry about additional panic attacks or their consequences • Significant maladaptive change in behavior related to the attacks

Adapted from DSM-5 [26].

A panic attack continues to be considered a noncodable event in the DSM-5, with only minor revisions, including removal of the “10-minute” window, changing “hot flushes” to “heat sensations,” and the re-ordering of the list of symptoms to increase clinical utility [26,143].

Compared to the DSM-IV-TR [144], changes to the diagnostic criteria for panic disorder largely consisted of minor phrasing changes to improve clinical utility, with the most substantial change being the title of the disorder [26,143]. The DSM-5 now lists agoraphobia (anxiety about having a panic attack in certain situations, which are avoided or endured with marked distress) as a separate codable disorder, whereas previously panic disorder could be diagnosed as “panic disorder with agoraphobia” or “panic disorder without agoraphobia” [26,145].

For a diagnosis of agoraphobia, a patient must have intense fear about at least two different types of situations, with the fear resulting from thoughts that escape may be difficult or help may be unavailable if panic-like symptoms occur (Table 13) [26,145]. The situations provoke anxiety and are avoided or endured with intense fear or anxiety, or may require that a companion be present. The resultant fear or anxiety is out of proportion to any actual danger from the situation, causes substantial functional impairment, and usually lasts for six months or longer [26].

**Table 13:** DSM-5 diagnosis of agoraphobia

• Marked fear or anxiety about ≥2 of the following 5 groups of situations: (1) Public transportation (e.g., traveling in automobiles, buses, trains, ships, or planes) (2) Open spaces (e.g., parking lots, market places, or bridges) (3) Being in shops, theatres, or cinemas (4) Standing in line or being in a crowd (5) Being outside of the home alone in other situations
• The individual fears or avoids these situations due to thoughts that escape might be difficult or help might not be available in the event of panic-like symptoms
• The agoraphobic situations almost always provoke fear or anxiety
• The situations are actively avoided, require presence of a companion, or endured with marked fear or anxiety
• The fear or anxiety is out of proportion to actual danger posed by agoraphobic situation
• The fear, anxiety, or avoidance is persistent, typically lasting ≥6 months
• The fear, anxiety, and avoidance cause clinically significant distress or functional impairment

Adapted from DSM-5 [26].

While the most up-to-date DSM-5 diagnostic criteria are presented here, the treatment data described within this section are based on studies involving patients meeting DSM-IV panic criteria (or older).

Establishing the context in which panic attacks occur, and whether there is any prior history of recurrent, unexpected panic attacks, is important for accurate diagnosis. Panic attacks frequently occur in other psychiatric disorders (e.g., MDD, PTSD), and medical conditions (e.g., cardiac, respiratory), and the DSM-5 has identified panic attacks as a specifier to be used in the absence of a diagnosable panic disorder [85]. Another disorder may better account for the panic attacks; for example, panic attacks in social situations may be SAD, those related to defined phobic objects or situations may be specific phobia, those related to reminders of traumatic events may be PTSD [26,85], and those related to being kidnapped by extraterrestrials may be schizophrenia [26]. Some medical conditions that can be associated with panic symptoms include hyper- or hypothyroidism, hypoglycemia, seizure disorders, and cardiac conditions [26,85]. Panic attacks may also be associated with intoxication or withdrawal from drugs of abuse, medications such as decongestants, stimulants, or beta-adrenergic agonist inhalers, or caffeine [85].

### Psychological treatment

CBT has been extensively studied, and is an efficacious psychological treatment for panic disorder (Level 1) [56,70,146,147]. In fact, CBT was significantly favored over medications for the treatment of panic disorder in a meta-analysis [71]. In a meta-analysis of 42 studies, exposure and combinations of exposure, cognitive restructuring and other CBT techniques had the most consistent evidence of efficacy for the treatment of panic disorder [56]. Strategies that included exposure were the most effective for panic measures. For measures of agoraphobia, combined strategies were more effective than single techniques, which did not result in significant improvements. Factors that improved the effectiveness of treatments were the inclusion of homework and a follow-up program [56]. Another meta-analysis also found that CBT that included interoceptive exposure was superior to relaxation therapy for panic symptoms [55]. CBT can be effectively delivered in both individual and group settings [56,148,149]. Conducting exposure in virtual reality appears to be effective when used as part of a CBT protocol [150-154].

Minimal intervention formats, such as self-help books (bibliotherapy) [75,76,155-158], treatment via telephone/videoconferencing [75,159-161], and internet-based CBT (ICBT) [75,79,162-169] have been shown to be more effective than wait-list or relaxation controls, as effective as face-to-face CBT, and may be cost-effective options particularly for agoraphobic patients who are unwilling or unable to attend a clinic. When using bibliotherapy, providing information all at one time was as effective as pacing [157], and therapist support does not appear to be essential [75,158]. Most ICBT programs have some therapist contact by either telephone or email, and once weekly contact appeared to be as effective as more frequent contact [168].

CBT panic disorder protocols usually involve 12-14 weekly sessions, but briefer strategies of six to seven sessions have been shown to be as effective [148,149,170]. In addition, compressing the duration of therapy by administering 13 sessions over three weeks has also been shown to be as effective as traditional weekly CBT [171]. Patients with higher baseline severity, disability, or comorbidity may have better outcomes with standard CBT [172]. CBT programs sometimes include one or more follow-up or “booster” sessions [170,173].

Predictors of decreased response to CBT were severity of panic disorder, strength of blood/injury fears, earlier age of initial onset of panic symptoms, comorbid social anxieties, and degree of agoraphobic avoidance [174,175]. Changes in symptoms are preceded by changes in beliefs during therapy [176], and change in beliefs and avoidance behaviors are considered key process variables [170,176].

Eye movement desensitization and reprocessing (EMDR) does not appear to offer advantages over the same strategy without the eye movement component for the treatment of panic disorder [177,178].

#### Combined psychological and pharmacological treatment

A meta-analysis of 21 trials found that combination psychotherapy and pharmacotherapy with antidepressants was superior to CBT or pharmacotherapy alone during the acute treatment phase and while medication was continued [179,180]. After termination of treatment, combined therapy was more effective than pharmacotherapy alone and was as effective as psychotherapy [179,180]. Prior meta-analyses have reported similar findings [54,146,181], suggesting that CBT alone or CBT combined with pharmacotherapy should be considered as first-line treatment.

A meta-analysis of the combination of psychotherapy and benzodiazepines included only three trials, and found no benefit to combination therapy compared with psychotherapy or medication alone [182]. The follow-up data suggested that the combination might be inferior to behavior therapy alone [182].

Adding self-administered CBT to SSRI therapy did not result in significant improvements overall, but patients did report a significantly greater rate of decline in fear of bodily sensations compared to medication alone [183]. Early results suggest a benefit of MBCT as an adjunct to pharmacotherapy in relieving anxiety and depressive symptoms in patients with panic disorder [184,185].

Providing CBT sessions around the time of medication discontinuation was associated with a lower relapse rate during follow-up among patients treated with antidepressants [186]. In addition, CBT has been shown to be helpful in facilitating benzodiazepine discontinuation [187,188].

A cost-effectiveness study found that combined CBT and pharmacotherapy was associated with a robust clinical improvement compared to usual care, with only a moderate increase in costs [189].

In a RCT, buspirone enhanced the effects of CBT in the short-term, but had no significant benefit over CBT alone during long-term follow-up [190].

Data on the efficacy of d-cycloserine as an adjunct to CBT are conflicting, with one study suggesting significant benefits at posttreatment and one-month follow-up [191], while another found an acceleration of symptom reduction in severely ill patients but no significant improvement in outcomes overall [192] compared to CBT plus placebo. Another compound acting at the *N*-methyl-D-aspartate (NMDA) receptor, Org 25935, demonstrated no benefit over placebo in augmenting CBT for panic disorder [193].

#### Long-term effects of psychological treatment

In naturalistic long-term follow-up studies, the benefits of CBT were maintained for up to three years [148,169,170,188]. At two-year follow-up, individual, group, and brief CBT were associated with lower relapse rates compared to the wait-list control [148]. A long-term follow-up study of patients who had become panic-free with exposure therapy found that 93% remained in remission after two years and 62% after 10 years [194].

A meta-analysis found that at six to 24 months follow-up, remission/response rates with the combination of psychotherapy and antidepressants continued to be superior to antidepressants alone, or to psychotherapy as long as therapy was continued [179,180].

### Pharmacological treatment

The management of patients with panic disorder should follow the principles discussed in Section 2. Pharmacological interventions that have good evidence for efficacy in treating panic disorder include SSRIs, TCAs, and other antidepressants, as well as benzodiazepines. Treatments that have been investigated for use in panic disorder have been assessed according to the criteria for strength of evidence (Tables 1 and 2) and are summarized in Tables 14 and 15.

**Table 14:** Strength of evidence for pharmacotherapy for panic disorder

Agent	Level of evidence	Agent	Level of evidence
***Antidepressants***			

**SSRIs**		**TCAs**	
Citalopram [198-200]	1	Clomipramine [199,211,213,232,233]	1
Fluoxetine [201-204]	1	Imipramine [207,224,233-240]	1
		
Fluvoxamine [195,205-210]	1	**MAOIs and RIMAs**	
Paroxetine [211-219]	1	Phenelzine [240]	2
Sertraline [183,220-224]	1	Moclobemide [204,232,241,242]	1*
Escitalopram [198]	2	Tranylcypromine [243]	3
		
Paroxetine CR [225]	2	**Other antidepressants**	
**SNRIs**		Reboxetine [200,219,244]	1
Venlafaxine XR [215,216,227-229]	1	Mirtazapine [203,245,246]	2
Duloxetine [230]	3	Bupropion SR [247,248]	3*
Milnacipran [231]	3		

***Other therapies***			

**Anxiolytics**		**Atypical antipsychotics**	
** Benzodiazepines**		Risperidone [217,267]	2
Alprazolam [234,249-254]	1	Olanzapine [268]	3
Clonazepam [218,250,255-258]	1	Quetiapine [267]	3
Lorazepam [251,259,260]	1	Adjunctive aripiprazole [269]	3
Diazepam [261-263]	1	Adjunctive olanzapine [270]	3
Adjunctive clonazepam [264,265]	1	Adjunctive risperidone [271]	3
		
Adjunctive alprazolam ODT [266]	3	**Anticonvulsants**	
		
**Other treatments**		Divalproex [272-275]	3
Buspirone [254,282]	1 (-ve)	Levetiracetam [276]	3
Trazodone [283]	2 (-ve)	Gabapentin [277]	2 (-ve)^†^
Propranolol [262,284,285]	2 (-ve)	Tiagabine [278,279]	2 (-ve)
Adjunctive pindolol [286]	2	Carbamazepine [280]	3 (-ve)
		Adjunctive divalproex [281]	3

*Conflicting data. ^†^No significant superiority over placebo in overall population, but significant benefits in subgroup of more severely ill patients. CR = controlled release; MAOI = monoamine oxidase inhibitor; ODT = orally disintegrating tablets; RIMA = reversible inhibitor of monoamine oxidase A; SNRI = serotonin–norepinephrine reuptake inhibitor; SR = sustained release; SSRI = selective serotonin reuptake inhibitor; TCA = tricyclic antidepressant; XR = extended release; (-ve) = negative.

**Table 15:** Recommendations for pharmacotherapy for panic disorder

First-line	Citalopram, escitalopram, fluoxetine, fluvoxamine, paroxetine, paroxetine CR, sertraline, venlafaxine XR
**Second-line**	Alprazolam, clomipramine, clonazepam, diazepam, imipramine, lorazepam, mirtazapine, reboxetine

**Third-line**	Bupropion SR, divalproex, duloxetine, gabapentin, levetiracetam, milnacipran, moclobemide, olanzapine, phenelzine, quetiapine, risperidone, tranylcypromine

**Adjunctive therapy**	**Second-line**: alprazolam ODT, clonazepam **Third-line**: aripiprazole, divalproex, olanzapine, pindolol, risperidone

**Not recommended**	Buspirone, propranolol, tiagabine, trazodone

CR = controlled release; ODT = orally disintegrating tablets; SR = sustained release; XR = extended release.

#### First-line agents

*SSRIs:* Evidence from meta-analyses [195-197] and RCTs supports the use of the SSRIs citalopram [198-200], fluoxetine [201-204], fluvoxamine [195,205-210], paroxetine [211-219], and sertraline [183,220,221,223,224] (all Level 1), as well as escitalopram [198] and paroxetine controlled-release (CR) [225] (both Level 2) for the treatment of panic disorder. In meta-analyses, SSRIs demonstrated significant improvements in panic symptoms, agoraphobic avoidance, depressive symptomatology, and general anxiety [195-197,226]. Effect sizes for SSRIs and TCAs are similar [195,196], although dropout rates may be lower with SSRIs [195].

*SNRIs:* Venlafaxine extended-release (XR) has been shown to be useful in reducing the severity of panic disorder symptoms in RCTs (Level 1) [215,216,227-229]. Two studies found significantly greater rates of panic-free patients compared with placebo [215,216] while two did not [228,229].

#### Second-line agents

*TCAs:* There is good evidence from RCTs to support the use of the TCAs clomipramine [199,211,213,232,233] and imipramine [207,224,233-240] in panic disorder (Level 1). In meta-analyses, TCAs have demonstrated efficacy for the treatment of panic symptoms and agoraphobia [195-197,226]. Efficacy is generally equivalent to SSRIs, however, since TCAs tend to be less well tolerated and have higher discontinuation rates than SSRIs [195], they are recommended as second-line options.

*Other antidepressants:* Although there is level 1 evidence to support the use of reboxetine [200,219,244], limited experience with this agent in Canada, and its side effect profile, which includes dry mouth, constipation, and insomnia [244], led to its recommendation as a second-line option. Mirtazapine has demonstrated efficacy for the treatment of panic disorder in several open trials [245,246] and one small RCT [203] (Level 2). It appears to be as effective as fluoxetine [203] and may be a useful second-line choice.

*Benzodiazepines:* Alprazolam [234,249-254], clonazepam [218,250,255-258], lorazepam [251,259,260], and diazepam [261-263] have demonstrated efficacy for the treatment of panic disorder (Level 1). While it has been suggested that alprazolam may be more effective, a meta-analysis found no evidence that it was superior to other benzodiazepines for the treatment of panic disorder [252]. Although benzodiazepines are second-line options, they may be useful at any time during therapy for the short-term management of acute or severe agitation or anxiety. They may also be useful at the initiation of SSRI treatment to hasten response (Level 1) [264-266].

#### Third-line agents

*MAOIs and RIMAs:* Results with moclobemide for the management of panic disorder have been conflicting (Level 1). In clinical trials, moclobemide demonstrated efficacy similar to that of clomipramine and fluoxetine [204,232], but was not superior to placebo [241,242]. However, significant efficacy in more severely ill patients [241], suggests it may be useful in treatment-resistant patients. In a RCT, phenelzine was more effective than placebo and as effective as imipramine (Level 2) [240]. In a small randomized, uncontrolled trial, tranylcypromine demonstrated efficacy for patients with comorbid panic and social anxiety disorders (Level 3) [243].

*Atypical antipsychotics:* There is some evidence that atypical antipsychotics may have some benefits in the treatment of patients with refractory panic disorder [217,267,268]. In a RCT, risperidone monotherapy was as effective as paroxetine (Level 2) [217]. Open-label data also support the use of risperidone [267], olanzapine [268], and quetiapine [267]. There are also open-label data supporting the use of some atypical antipsychotics as adjunctive therapy (see below).

*Other therapies:* The antidepressants duloxetine [230], milnacipran [231], and bupropion sustained release (SR) [247,248] have shown some efficacy in open trials, as have the anticonvulsants divalproex [272-275] and levetiracetam [276] (all Level 3). In a RCT, gabapentin was superior to placebo in patients who were more severely ill, but not in the overall group (Level 2, negative) [277]. These agents are recommended only as third-line options in patients with refractory panic disorder.

#### Adjunctive therapy

There is good evidence that adjunctive clonazepam [264,265] (Level 1), and open-label evidence that adjunctive alprazolam orally-disintegrating tablet (ODT) [266] (Level 3), used short-term (<8 weeks including taper) at the initiation of SSRI treatment, can lead to a more rapid response [264-266].

In a RCT, pindolol added to fluoxetine therapy in patients with treatment-resistant panic disorder was associated with significant improvement in panic disorder symptoms compared with fluoxetine plus placebo (Level 2) [286]. Open-label data also support the use of the atypical antipsychotics aripiprazole [269], olanzapine [270], and risperidone [271] (all Level 3), as well as the anticonvulsant divalproex [281], as adjunctive strategies for patients with treatment-resistant panic disorder.

#### Not recommended

Buspirone (Level 1, negative) [254,282], propranolol (Level 2, negative) [262,284,285], tiagabine [278,279] (Level 2, negative), and trazodone (Level 2, negative) [283] have not demonstrated efficacy and are not recommended for the treatment of panic disorder. Carbamazepine (Level 3, negative) [280] also does not appear to be effective in this disorder.

#### Maintenance pharmacological treatment

In long-term, open, follow-up studies, citalopram [287,288], fluoxetine [204,288], fluvoxamine [288], paroxetine [288-290], and moclobemide [204], as well as clomipramine [287,289] and imipramine [291,292] demonstrated maintenance of benefits and continued improvements over six months to three years of ongoing treatment. In a RCT, sertraline and imipramine were equally effective over a six month period [224]. However, in another RCT, imipramine was not superior to placebo in the proportion of panic-free patients after eight months of therapy [293].

Venlafaxine XR [294] and imipramine [295] have been shown to prevent relapse in randomized, placebo-controlled, discontinuation studies. After three months of acute treatment, relapse rates were significantly lower with ongoing venlafaxine XR [294] or imipramine [295] therapy compared with switching to placebo during six to 12 months of follow-up.

Benzodiazepines are generally recommended for short-term use only. However, several trials have demonstrated the benefits of up to two years of alprazolam maintenance therapy [291,293]. There was no evidence of tolerance, but up to one-third of patients were unable to discontinue therapy [293]. The efficacy of clonazepam was maintained over a three-year course of treatment [290], and patients who had been asymptomatic for at least one year were able to successfully discontinue the medication, using a slow tapering strategy over four to seven months, and improvement in panic disorder was maintained [296].

### Biological and alternative therapies

*Biological therapies:* In open-label case series, noninvasive brain stimulation using a radioelectric asymmetric conveyor (REAC) demonstrated efficacy for panic symptoms and agoraphobia (Level 3) [297,298]. A small case series suggested repetitive transcranial magnetic stimulation (rTMS) could improve panic and anxiety in patients with panic disorder with comorbid MDD (Level 4) [299]. However, a small RCT found no additional benefit of rTMS compared to sham rTMS as an add-on to SSRI therapy in patients with panic disorder (Level 2, negative) [300].

*Alternative therapies:* In a RCT, capnometry-assisted respiratory training was as effective as cognitive training in reducing panic symptom severity and panic-related cognitions and improving perceived control (Level 2) [301]. However, breathing training did not significantly improve reactivity or recovery after a respiratory challenge in another small trial (Level 2, negative) [302]. In a RCT, patients with panic disorder randomized to the exercise groups (plus paroxetine or placebo) had a trend toward better improvement compared to relaxation training, but this was not significant (Level 2, negative) [303]. However, in an open cross-over study, acute aerobic exercise was found to reduce anxiety as well as panic attack frequency and intensity in patients with panic disorder compared to a quiet rest condition (Level 3) [304]. These therapies may be useful for some patients; however, more data are needed.

### Summary

As much as 40% of the general population has experienced a panic attack at some point in their lifetime. However, patients with actual panic disorder experience recurrent, unexpected panic attacks as well as persistent concern or behavioral change around further attacks.

Data support pharmacotherapy, CBT alone, and CBT combined with pharmacotherapy as initial treatments for panic disorder. CBT alone may be insufficient in patients with comorbid moderate-to-severe major depression, or in those with severe, frequent panic attacks, or rapid worsening of agoraphobia, and/or suicidal ideation, as well as in situations where one might consider initial rescue treatment with a benzodiazepine to minimize or stop the panic attacks while waiting the 4-12 weeks for the first-line pharmacotherapy to become effective. Also there are patients who are not motivated to participate in CBT (preferring medication as initial treatment) or are too fearful to engage in any kind of exposure before being treated with a first-line pharmacotherapeutic agent. At the very least, if agoraphobic distress or avoidance persists, these patients need instruction and support to engage in exposure exercises. For panic symptoms, strategies should include exposure; and combined strategies should be considered for patients with agoraphobia. CBT can be effectively delivered in both individual and group settings, as well as via self-help books, virtual reality, and internet-based programs. The benefits of CBT are maintained during follow-up. In addition, data suggest that combination of psychotherapy and pharmacotherapy may be superior to pharmacotherapy alone during follow-up.

Pharmacotherapeutic approaches should begin with a first-line agent. If response to optimal dosing is inadequate or the agent is not tolerated, treatment should be switched to another first-line agent before considering second-line medications. First-line options for the treatment of panic disorder include citalopram, fluoxetine, fluvoxamine, paroxetine, sertraline, venlafaxine XR, escitalopram, or paroxetine CR. Second-line choices include the TCAs (clomipramine and imipramine), mirtazapine, reboxetine, or benzodiazepines (alprazolam, clonazepam, lorazepam, and diazepam).

Patients who do not respond to first- or second-line agents are considered to have treatment-refractory illness. In such patients it is important to reassess the diagnosis and consider comorbid medical (e.g., ischemic heart disease) and psychiatric conditions (e.g., SUDs) that may be affecting response to therapy. Third-line agents, adjunctive therapies, as well as biological and alternative therapies may be useful when patients fail to respond to an optimal treatment trial of first- and second-line therapies used alone and in combination.

## Specific phobia

### Epidemiology

A specific phobia is an intense fear of a specific object or situation and is usually associated with avoidance of the feared object. The most prevalent phobia types include animal (e.g., insects, snakes), natural environment (e.g., heights, storms, water), situational (e.g., flying, enclosed spaces), and blood-injection-injury (B-I-I) (e.g., blood, dentists, hospitals) [305,306]. Large US and European epidemiologic surveys report lifetime prevalence estimates of 10-13% and 12-month prevalence rates of 7-9% [2,3,305,307]. Rates among adolescents may be particularly high with lifetime prevalence estimates of 36.5% and 12-month prevalence rates of 27.3% being reported [308]. Specific phobias are more common in women than men [306]. Age of onset is usually in the range of five to 12 years (median: seven years) [2]; however, this varies by type of phobia. Animal and B-I-I phobias generally begin in childhood, whereas situational phobias (e.g., driving phobia, claustrophobia) have a later onset, typically during late adolescence or early adulthood [306].

Specific phobias are associated with significant distress, regardless of the number of feared stimuli reported [305]. Specific phobias have a negative impact on social/occupational functioning and lead to restriction of usual daily activities, which increases with an increasing number of fears [305].

#### Comorbidities

Specific phobias tend to co-occur with other specific phobias, with less than 10% of patients having only one fear [305]. The mean number of fears, in one survey, was three [305]. In addition, specific phobias are frequently comorbid with other psychiatric disorders, including SUDs, mood disorders, and other anxiety or related disorders (particularly panic disorder, SAD, and GAD), as well as personality disorders [305,309,310].

### Diagnosis

To receive a DSM-5 diagnosis of specific phobia a patient must experience marked (intense) fear or anxiety about a specific object or situation, which is associated with significant distress or functional impairment (Table 16) [26]. The object or situation will be actively avoided or endured with intense anxiety. Compared to the DSM-IV-TR criteria for specific phobia [144], few changes were made in the DSM-5 [26,306]. Of note, recognition that the fear is excessive or unreasonable has been removed and a new criterion stating “the fear or anxiety is out of proportion to danger posed” has been added. Avoidance has been clarified as “actively avoided” to distinguish the avoidance seen in specific phobias from passive avoidance that may occur for other reasons [26,306].

**Table 16:** DSM-5 diagnosis of specific phobia

• Marked fear or anxiety about a specific object or situation (e.g., flying, seeing blood) • The phobic object or situation almost always provokes immediate fear or anxiety and is actively avoided or endured with marked fear or anxiety • The fear or anxiety is out of proportion to the actual danger posed by the specific object or situation • The fear, anxiety, or avoidance is persistent, typically ≥6 months • There is marked distress or functional impairment

Adapted from DSM-5 [26].

While the most up-to-date DSM-5 diagnostic criteria are presented here, it is important to note that most of the treatment data described within this section are based on patients meeting DSM-IV criteria (or older).

Specific phobias are delineated into five types: animal type, natural environment type, B-I-I type, situational type, or other type (Table 17) [26]. The fear of contracting an illness has been removed because of high relatedness to OCD and anxiety disorder related to medical condition [26].

**Table 17:** Specific phobia specifiers in DSM-5

Specifier	Examples
Animal	Spiders, insects, dogs

Natural environment	Heights, storms, water

Blood-injection-injury	Needles, invasive medical procedures

Situational	Airplanes, elevators, enclosed spaces

Other	Choking or vomiting. In children, loud sounds or costumed characters

Adapted from DSM-5 [26].

Specific phobias can be difficult to distinguish from panic disorder [311]. It is important to consider the focus of apprehension (e.g., fear of crashing while on an airplane versus fear of having a panic attack on an airplane), the types of panic attacks experienced (e.g., expected versus unexpected), and the range of situations associated with fear and avoidance [311].

### Psychological treatment

Psychosocial interventions, particularly exposure-based treatments, are the treatments of choice and are associated with a high degree of success in providing remission of specific phobias [311]. Both *in vivo* exposure and virtual reality exposure (VRE) can be effective [57,311,312], with *in vivo* exposure being shown to be superior to alternative types (e.g., imaginal, virtual reality, etc.) at posttreatment but not at follow-up [57].

In general, exposure-based therapy has been shown to be more effective if: sessions are grouped closely together; exposure is prolonged, real (not imagined), and provided in multiple different settings; and there is some degree of therapist involvement (not entirely self-directed) [32,311]. While one-session treatments have demonstrated efficacy [313], a meta-analysis found that a greater number of sessions predicted more favorable outcomes [57].

There is no evidence that either flooding or gradual exposure is more effective [314], however, progressive exposures are generally more tolerable to patients [311]. An example of graded exposure in a patient with arachnophobia would be to look at pictures of spiders, hold a rubber spider, look at a live spider in a jar, touch the jar containing the spider, stand two feet from a live spider, and finally touch a live spider. This approach can be used to guide exposure depending on the patient’s symptom severity and tolerance to each level of exposure.

While a meta-analysis of 33 RCTs of psychological approaches found that treatment outcomes were not moderated by type of specific phobia [57], studies have suggested that certain subtypes may respond more favorably to specific types of treatment (Table 18).

**Table 18:** Psychological treatments with demonstrated efficacy in specific phobias

Psychological treatment	Phobia
Exposure-based treatments	All specific phobias [57,311,312]

Virtual reality exposure	Heights [327-329], flying [319,321-324], spiders [331,332], claustrophobia [330]

Computer-based self-help programs	Spiders [334,335], flying [323], small animals [336,337]

Applied muscle tension (exposure combined with muscle tension exercises)	Blood-injection-injury type [311,315,316]

Cognitive therapy and exposure	Dental [318], flying [319,320]

For patients with B-I-I phobias, exposure therapy combined with muscle tension exercises (applied tension) designed to prevent fainting [311] has been shown to be effective [315,316]. Use of stress-reducing medical devices, such as decorated butterfly needles and syringes, has been shown to significantly reduce needle phobia and stress in both pediatric and adult patients [317]. CBT reduced avoidance of oral injections and decreased anxiety in patients with dental phobias [318].

Fear of flying has been effectively treated with group CBT [319,320]. In addition, computer-generated VRE has demonstrated efficacy [319,321-324], which was comparable to standard exposure therapy in several studies [322,324], and can have long-term benefits [325,326]. Bibliotherapy was found to be less effective than VRE or CBT for patients with fear of flying [319]. VRE has also been shown to be effective for patients with a fear of heights [327-329], and those with claustrophobia [330]. This approach may also be useful for treating fears for which *in vivo* exposure may not be practical (e.g., fear of storms) [32].

Arachnophobia has been successfully treated with *in vivo*[331] and VR [331,332] exposure, with little difference between the two modalities [331]. A spiderless form of VRE, which presented images that were not spiders, but had some of the characteristics of spiders, was shown to be useful in patients with severe arachnophobia who were reluctant to undergo direct exposure or VRE [333]. An internet-based self-help program was associated with improvement, but was not as effective as one session of *in vivo* exposure at the post-treatment assessment, although results were similar at follow-up [334]. However, even one session of VRE was associated with greater fear reduction compared to a control group, and may be a useful self-help intervention to reduce fear of spiders [335]. Computer-based self-help has also shown promise for other small-animal phobias (e.g., cockroaches, mice) [336,337].

#### Combined psychological and pharmacological treatment

It has been speculated that d-cycloserine, a partial agonist at the NMDA receptor, may improve extinction of fear in patients with phobias undergoing behavioral exposure therapy [338]. In a RCT (n=28), d-cycloserine as an adjunct to VRE resulted in significantly larger reductions of acrophobia symptoms compared with VRE alone [338]. In another study (n=100), adjunctive d-cycloserine did not improve the reduction of spider fears compared to exposure-based therapy alone, however, patients had heightened, but subclinical, spider fears [339].

In two RCTs, use of adjunctive cortisol, a glucocorticoid, significantly enhanced the benefits of exposure therapy compared with placebo in patients with acrophobia (n=40) [340] and arachnophobia (n=20) [341], with evidence suggesting that cortisol may facilitate the extinction of phobic fear at follow-up.

Enhanced emotional memory may be stimulated through elevated noradrenaline levels, and data suggest that yohimbine hydrochloride, a noradrenaline agonist, can facilitate fear extinction. In RCTs, there were no significant VRE-enhancing effects with adjunctive yohimbine compared with placebo in patients with fear of flying (n=48) [342] or claustrophobia (n=24) [343]. However, in the claustrophobia study, patients treated with yohimbine showed greater improvements in outcomes at the one-week follow-up [343].

In contrast, naltrexone was found to render one-session exposure therapy less effective compared with placebo or no treatment in 15 patients with specific phobias (animals) [344].

#### Long-term effects of psychological treatment

Long-term treatment of specific phobia is rare. As discussed above, CBT and exposure therapies have demonstrated sustained benefits at long-term follow-up assessments [325,326].

### Pharmacological treatment

There is a minimal role for pharmacotherapy in the treatment of specific phobias, largely due to the lack of research on medications in this condition, and the success of exposure-based therapies [32,311].

Antidepressants have been investigated in two small RCTs [345,346]. In a small RCT, paroxetine was significantly more effective than placebo in resolving anxiety in patients with specific phobias (n=11) [345]. Similarly, escitalopram was associated with a strong treatment effect in a small RCT (n=12); however, the trial was under-powered to show statistically significant superiority over placebo on the primary outcome [346]. In addition, cases of successful treatment of flying phobias with fluoxetine [347], and storm phobia with fluvoxamine [348], have been reported.

Benzodiazepines have usually been assessed as adjuncts to exposure therapy, and these studies have found no additional benefit with medication [349-351]. Benzodiazepines are often used in clinical practice to provide acute symptom relief when it is necessary for a patient with a specific phobia to face a feared situation (e.g., dental procedure, magnetic resonance imaging [MRI], unexpected flight) [32]. Nasal midazolam has proven useful in facilitating MRI in claustrophobic patients [352,353].

### Summary

Specific phobia is quite common, particularly among adolescents. Patients with specific phobia exhibit an intense fear or anxiety about a specific object or situation which is associated with significant distress or functional impairment. The most prevalent phobia types include animal, natural environment, situational, and B-I-I.

Exposure-based techniques, including virtual exposure, are highly effective, and are the foundation of treatment for specific phobias. Pharmacotherapy is generally unproven, and thus not a recommended treatment for most cases.

## Social anxiety disorder

### Epidemiology

SAD is one of the most common anxiety disorders, with lifetime prevalence estimates ranging from 8-12% among the international general population [2,354-356]. It is more common in women than men [355,357-360], and higher rates have been reported in developed (6.1%) versus developing (2.1%) countries [361]. SAD has an early age of onset, typically during adolescence (mean 12 years), and tends to have a chronic and unremitting course [2,362,363]. Factors such as low educational achievement, low socioeconomic status, being single or separated, and having comorbid MDD have been associated with a higher prevalence of SAD in epidemiological studies [359,360,364].

SAD is associated with significant impairments including problems with educational and occupational performance, family functioning, and an overall reduced QoL [14,15,17,354,363,365-369]. SAD also confers a substantial economic burden upon afflicted individuals and society in terms of work days missed and health care costs [370,371]. Canadians with SAD were twice as likely to report at least one disability day in the past two weeks, compared to those without SAD [356].

#### Psychiatric comorbidity

SAD is associated with significant comorbidity, with up to 72% of patients reporting criteria for another psychiatric disorder [372]. The highest rates of comorbidity have been found with MDD and other anxiety or related disorders [355,356,360]. Avoidant personality disorder [373], body dysmorphic disorder [374,375], SUD [356,376], ADHD [377,378], and schizophrenia [379] also commonly occur with SAD.

### Diagnosis

SAD is characterized by a persistent fear that in social and performance situations the individual will say or do something that will lead to humiliation, embarrassment, or negative evaluation by others (Table 19) [26]. Social situations are actively avoided or endured with distress, and the individual recognizes the fears as excessive or unreasonable. The avoidance or anxiety induced by these fears incurs significant functional impairment and distress [144]. Compared to the DSM-IV-TR [144], changes to the diagnostic criteria for SAD in the DSM-5 have been minimal, largely consisting of minor phrasing changes to improve clinical utility [26]. The criterion that the “person recognizes that the fear is excessive or unreasonable” has been changed to “out of proportion to the actual threat posed by the social situation.” Since patients with SAD are often unable to recognize that their fear may be excessive the clinician may be in a better position to judge this.

**Table 19:** DSM-5 diagnosis of SAD (social phobia)

• Marked fear or anxiety about social situations in which the person may be exposed to scrutiny by others
• Fear that actions or showing anxiety symptoms will cause negative evaluation (e.g., embarrassment, humiliation) or offend others
• The social situation: ○ Almost always provokes fear or anxiety ○ Is actively avoided or endured with marked fear or anxiety
• The fear, anxiety, or avoidance: ○ Is out of proportion to the actual threat posed by the social situation ○ Is persistent, typically ≥6 months ○ Causes significant distress or functional impairment
• If another medical condition is present (e.g., stuttering, obesity), the disturbance is unrelated or out of proportion to it
• Specify “performance only” if the fear is restricted to speaking or performing in public

Adapted from DSM-5 [26].

The DSM-IV-TR criteria excluded social fears/avoidance associated with and secondary to medical conditions, however, the DSM-5 recognizes that SAD may be secondary to a medical condition. Some patients experience excessive social anxiety about their medical symptoms (e.g., stuttering, tremulousness from Parkinson’s disease, obesity, disfigurement from burns or injury), and may experience disability due to their social anxiety [26].

In addition, the “generalized” subtype specifier included in DSM-IV-TR has been removed, while the “performance only” specifier has been added [26,380] for DSM-5. This change was made because there was little supporting evidence for the generalized specifier, and the evidence that SAD symptoms fall along a continuum of severity characterized by the number of fears [380]. The “performance only” specifier appears to represent a subset of SAD patients typically experiencing impairment from performance fears primarily related to their professional lives [26].

While the most up-to-date DSM-5 diagnostic criteria are presented here, it is important to note that all of the treatment data described within this section are based on patients meeting DSM-IV criteria (or older).

### Psychological treatment

Psychological treatment, in the form of CBT, is considered to be the gold-standard nonpharmacological treatment in SAD. Cognitive techniques involved in CBT for SAD include restructuring and challenging of maladaptive thoughts, while the behavioral component is typically in the form of exposure therapy. The efficacy of CBT compared with placebo, treatment-as-usual, or wait-list conditions, is supported by many RCTs as well as meta-analytic evidence [58,59,70,71,381]. Although results vary, several studies of acute SAD treatment have also found a similar efficacy between CBT and pharmacotherapy [382-387]. Some reports suggest that after treatment discontinuation, gains achieved with CBT may persist longer than those achieved with pharmacotherapy [388,389]. CBT for SAD can be administered in group or individual formats. Although some studies have reported that individual CBT is superior to group CBT [390,391], meta-analyses have failed to find significant differences in efficacy between the two modalities [58,59,381].

The treatment literature has also examined the efficacy of the individual components of CBT. There is evidence to support the effectiveness of exposure therapy alone [389,392], however the efficacy of exposure alone compared with CBT is equivocal in the current treatment literature [392-395].

There are several variants of CBT that have been examined in the literature. For example, videotaped feedback was not shown to enhance the effects of exposure-based treatment [396]. However, CBT with VRE was found to be more effective than wait-list control and as effective as CBT with imaginal or *in vivo* exposure according to two meta-analyses [80,150].

A form of CBT focused on interpersonal behavior found similar improvements in social anxiety compared to standard CBT but also increased relationship satisfaction and social approach behaviors [397]. Evidence to support interpersonal therapy (IPT) in SAD is conflicting [398-400]; while some results have been negative [398], it is likely that IPT is more effective than wait-list control [399], but less effective than traditional CBT [399,400].

Similarly, while less effective than traditional CBT, mindfulness-based therapy (MBT) has been associated with improvements in symptoms of SAD [401]. In addition, small studies of attentional bias training suggest there may be some benefit associated with training patients to disengage from negative social cues, but data are conflicting [402,403].

ICBT is a newer treatment that may increase the availability of CBT for anxiety and mood disorders in the future. Studies have evaluated this treatment in comparison to individual and group CBT. ICBT has demonstrated efficacy in RCTs of SAD, significantly improving social anxiety symptoms compared to wait-list control conditions [404-410]. Most ICBT programs include minimal therapist contact via email [404-410] or telephone [405,409]. Many programs involve a component of interaction with other participants through the use of internet discussion groups [411]. However, it remains unclear whether the therapist component is necessary, and studies comparing guided with unguided ICBT have yielded conflicting results. In one RCT, clinician-assisted ICBT was more effective than a self-guided ICBT, and the self-guided ICBT was not significantly better than the wait-list condition [406]. Similarly, a self-help program augmented with minimal therapist contact was more useful than a pure self-help strategy [412]. However, several other RCTs have found that unguided ICBT self-help was as effective as ICBT with therapist involvement [410,411]. A few ICBT programs included face-to-face *in vivo* exposure sessions [409,413], but one RCT found that adding this component did not significantly improve outcomes versus ICBT with self-directed exposure [413]. In addition, several RCTs have shown ICBT (with minimal therapist contact) to be as effective as face-to-face CBT [414,415], while being more cost-effective [416]. As with other RCTs, research on ICBT has involved pre-screening of participants in-person or by telephone, with posttreatment and follow-up assessments by telephone or through self-report measures. Little is known about the effectiveness of self-administered treatments (ICBT or self-help books) used with no pre-screening or planned follow-up contacts.

### Combined psychological and pharmacological treatments

When used in combination, pharmacotherapy has not been shown to add to the benefits of CBT in some studies [387,417], while one study found the combination of phenelzine and CBT superior to either modality alone [418]. D-cycloserine has also been found to enhance treatment outcomes when used during exposure exercises as an adjunct to exposure alone [419,420]. In addition, a study of psychodynamic group therapy with or without the addition of clonazepam also found combination treatment to be superior to clonazepam treatment alone [421].

### Long-term effects of psychological treatment

The benefits of CBT have been found to be maintained at six to 12 month follow-up visits [58,382,390,393,409,413,422,423], with sustained improvement being reported at five years posttreatment [424,425]. Long-term assessments post-ICBT have shown sustained improvement at one to five years follow-up [409,413,423,424]. Long-term benefits with psychotherapy appear to be more enduring than those of pharmacotherapy after treatment discontinuation [388,389].

## Pharmacological treatment

The management of patients with SAD should follow the principles discussed in Section 2. Pharmacological interventions that have good evidence for efficacy in treating SAD include SSRIs, SNRIs, anticonvulsants, and benzodiazepines. Treatments that have been investigated for use in SAD have been assessed according to the criteria for strength of evidence (Tables 1 and 2) and are summarized in Tables 20 and 21.

**Table 20:** Strength of evidence of pharmacotherapy for SAD

Agent	Level of evidence	Agent	Level of evidence
***Antidepressants***			

**SSRIs **[58,426-429]	1	**TCAs**	
Escitalopram [430-432]	1	Clomipramine [458,459]	3
Fluvoxamine [433-435]	1	Imipramine [460]	3 (-ve)
		
Fluvoxamine CR [436,437]	1	**MAOIs and RIMAs**	
Paroxetine [431,438-444]	1	Phenelzine [384,386,418,461,462]	1
Sertraline [445-448]	1	Moclobemide [417,462-466]	1*
		
Fluoxetine [382,387,449]	1*	**Other antidepressants**	
Citalopram [450,451]	2	Mirtazapine [467,468]	1*
Paroxetine CR [452]	2	Bupropion SR [469]	3
Adjunctive paroxetine [453]	3		
**SNRIs**			
Venlafaxine XR [439,441,454,255,456]	1		
Duloxetine [457]	2		

***Other therapies***			

**Anxiolytics**		**Anticonvulsants**	
** Benzodiazepines**		Pregabalin [474,475]	1
Clonazepam [385,470,471]	1	Gabapentin [476,477]	2
Alprazolam [386]	2	Levetiracetam [478-480]	2 (-ve)
Bromazepam [472]	2	Divalproex [481]	3
Adjunctive clonazepam [473]	2 (-ve)	Tiagabine [477,482]	3
		Topiramate [483]	3

**Other treatments**		**Atypical antipsychotics**	
Atenolol [461,484]	1 (-ve)	Olanzapine [493]	2
Buspirone [383,485]	1 (-ve)	Quetiapine [494,495]	2 (-ve)
Atomoxetine [486,487]	1*	Adjunctive aripiprazole [496]	3
Propranolol [488]	2 (-ve)	Adjunctive risperidone [271]	3
Selegiline [489]	3		
Pergolide [490]	3 (-ve)		
Adjunctive buspirone [491]	3		
Adjunctive pindolol [492]	2 (-ve)		

*Conflicting data. CR = controlled release; MAOI = monoamine oxidase inhibitor; RIMA = reversible inhibitor of monoamine oxidase A; SNRI = serotonin–norepinephrine reuptake inhibitor; SR = sustained release; SSRI = selective serotonin reuptake inhibitor; TCA = tricyclic antidepressant; XR = extended release; (-ve) = negative.

**Table 21:** Recommendations for pharmacotherapy for SAD

First-line	Escitalopram, fluvoxamine, fluvoxamine CR, paroxetine, paroxetine CR, pregabalin, sertraline, venlafaxine XR
**Second-line**	Alprazolam, bromazepam, citalopram, clonazepam, gabapentin, phenelzine

**Third-line**	Atomoxetine, bupropion SR, clomipramine, divalproex, duloxetine, fluoxetine, mirtazapine, moclobemide, olanzapine, selegiline, tiagabine, topiramate

**Adjunctive therapy**	**Third-line:** aripiprazole, buspirone, paroxetine, risperidone **Not recommended:** clonazepam, pindolol

**Not recommended**	Atenolol^*^, buspirone, imipramine, levetiracetam, propranolol^*^, quetiapine

CR = controlled release; SR = sustained release; XR = extended release.

*Beta-blockers have been successfully used in clinical practice for performance situations such as public speaking.

Note: although there is limited evidence for citalopram in SAD, it is likely as effective as the other SSRIs, in contrast there are negative trials of fluoxetine in SAD suggesting it may be less effective than other SSRIs [382,449].

### First-line agents

*Antidepressants:* Meta-analyses demonstrate that SSRIs and SNRIs are significantly more effective than placebo [58,426-429] and RIMAs [426,428] for the treatment of SAD. There is level 1, RCT evidence supporting the use of the SSRIs escitalopram [430,431], fluvoxamine [433-435], fluvoxamine CR [436,437], paroxetine [431,438-444], and sertraline [445-448], as well as the SNRI venlafaxine XR [439,441,454-456], for the first-line treatment of SAD. There is also good evidence for the efficacy of paroxetine CR (Level 2) [452].

*Pregabalin:* Pregabalin has also demonstrated efficacy versus placebo for the treatment of SAD in RCTs at higher (600 mg/day) but not lower dose levels (150-300 mg/day) (Level 1) [474,475]. Although there is Level 1 evidence for pregabalin, it is not clear how its efficacy compares to that of SSRIs. In addition, SSRIs may have a broader spectrum of efficacy for common comorbid conditions.

### Second-line agents

*Benzodiazepines:* In RCTs, the benzodiazepines clonazepam (Level 1) [470][385,471], alprazolam [386], and bromazepam [472] (both Level 2) have demonstrated efficacy in the treatment of SAD.

Although, a meta-analysis found benzodiazepines to be as effective as SSRIs [58], these agents are recommended as second-line options because of the lack of effect on common comorbidities and the potential for abuse/dependence in individuals with a history of SUDs.

*Antidepressants:* In RCTs, citalopram was found to be significantly more effective than placebo [451], and as effective as moclobemide [450] (Level 2). Although there is limited evidence for citalopram in SAD, it is likely as effective as the other SSRIs.

The efficacy of phenelzine has been established in multiple RCTs (Level 1) [384,386,418,461,462]; however, this agent is recommended as a second-line option because of concerns regarding dietary restrictions, drug interactions, and the potential for hypertensive crisis.

*Anticonvulsants:* Gabapentin was significantly more effective than placebo in a RCT [476], and as effective as tiagabine in a small cross-over study (Level 2) [477].

### Third-line agents

*Antidepressants:* Results with fluoxetine have been mixed (Level 1, conflicting) [382,387,449]. A large RCT found that fluoxetine was more effective than placebo and as effective as CBT [387]. However, in two other small RCTs, fluoxetine alone or when added to self-exposure showed no benefit over placebo, with or without self-exposure [382,449]. These negative trials with fluoxetine suggest it may be less effective than other SSRIs [382,449].

Similarly, results with moclobemide have also been mixed (Level 1, conflicting) [417,462-466], with some RCTs demonstrating significantly higher response rates with moclobemide compared with placebo (Level 1) [462-464], while others have not [465,466]. Moclobemide was found to be superior to CBT early in treatment; however, after six months CBT was found to be superior.

Data from two small RCTs assessing mirtazapine were also mixed (Level 1, conflicting), with one showing significant improvements over placebo [468] and the other showing no differences [467].

In a dose-finding study in which patients treated with open-label duloxetine 60 mg/day were randomized to continue or double their dose, both doses improved symptoms, but there was no significant advantage to the higher dose (Level 2) [457].

Small open-label trials have also suggested that bupropion SR [469] and clomipramine [458,459] (both Level 3) may be effective in patients with SAD.

*Anticonvulsants:* Open-label studies have demonstrated some efficacy with divalproex [481], topiramate [483], and tiagabine [482] (all Level 3). In addition, tiagabine was comparable to gabapentin in a small RCT, crossover study in eight adults [477].

*Other treatments:* Olanzapine was effective in a small RCT (Level 2) [493], and selegiline demonstrated efficacy in a small, open-label trial (Level 3) [489]. In a RCT, atomoxetine significantly improved SAD symptoms compared with placebo [487]; however, in a another small RCT, atomoxetine showed no significant difference in outcomes compared with placebo (Level 1, conflicting) [486].

All of these agents are recommended as third-line options, and may be useful in refractory patients after first- and second-line monotherapies and adjuncts have been unsuccessful.

### Adjunctive therapy

Adjunctive strategies have generally been studied in patients who have had an inadequate response to antidepressant therapy and can be considered for patients with treatment-resistant SAD.

*Third-line adjunctive therapies:* Open-label studies and case series have suggested that patients with refractory SAD may benefit from adjunctive therapy with aripiprazole [496], risperidone [271], buspirone [491], or paroxetine [453] (all Level 3).

*Not recommended adjunctive or combination therapies:* In RCTs, clonazepam [473] combined with paroxetine and pindolol augmentation of paroxetine [492] (both Level 2, negative) were not significantly superior to placebo in augmenting the effects of SSRI treatment for SAD.

### Not recommended

In RCTs there was no evidence of benefits with the beta-blockers atenolol (Level 1, negative) [461,484] or propranolol (Level 2, negative) [488], or for the following treatments: buspirone [383,485], levetiracetam [478-480] (both Level 1, negative), or quetiapine (Level 2, negative) [494,495]. These agents are not recommended for SAD. Imipramine [460] and pergolide (both Level 3, negative) [490] also do not appear to be effective in this disorder.

### Maintenance pharmacological treatment

Long-term therapy has been evaluated in relapse prevention and naturalistic follow-up studies. Relapse-prevention studies are those in which responders to medication are randomized to continued active treatment or placebo. A meta-analysis of four relapse prevention studies included 760 patients with SAD and found a highly significant reduction in relapse rates with continued SSRI treatment compared with placebo over three to six months. The relative risk (RR) for relapse was 0.39 (95% CI 0.30–0.49) and number needed to treat (NNT) was 3.57 (95% CI 2.94–4.76) [497]. The anticonvulsant pregabalin has also demonstrated reductions in relapse rates over six months [498].

In RCTs, escitalopram [431], fluvoxamine CR [499], and venlafaxine XR [456] have demonstrated continued improvement compared with placebo over approximately six months. Additional open follow-up data support the long-term efficacy of moclobemide over six to 24 months [464,500].

## Biological and alternative therapies

*Biological therapies:* In an open-label study, neuro psycho physical optimization-radio electric asymmetric conveyor (NPPO-REAC) (a brain stimulation technique) was as effective as sertraline for the treatment of SAD (Level 3) [501].

*Alternative therapies:* St John’s wort failed to demonstrate superiority over placebo, and is not recommended for the treatment of SAD (Level 2, negative) [502].

## Summary

SAD is one of the most common anxiety disorders, occurring more often in women than men. SAD has a negative impact on QoL, functional and occupational outcomes, and is often associated with other comorbid disorders, including MDD and other anxiety and related disorders. SAD is characterized by intense fear or anxiety relating to social or performance situations where the individual is exposed to scrutiny by others. These situations are often actively avoided.

CBT and exposure therapy alone are effective first-line options for the treatment of SAD, although limited data suggest that CBT may be more effective in maintaining benefits during follow-up. VRE and internet-based programs have also demonstrated efficacy. The benefits of CBT are maintained over one to five years of follow-up. CBT and pharmacotherapy appear to have similar efficacy for the acute treatment of SAD, but after treatment discontinuation, gains achieved with CBT appear to persist longer than those achieved with pharmacotherapy. In most studies, adding pharmacotherapy has not been shown to increase the benefits of CBT.

Pharmacotherapeutic approaches should begin with a first-line antidepressant such as escitalopram, fluvoxamine, fluvoxamine CR, paroxetine, paroxetine CR, sertraline, or venlafaxine XR, or the anticonvulsant pregabalin. If response to optimal doses is inadequate or the agent is not tolerated, therapy should be switched to another first-line agent before considering a second-line medication. Second-line choices include the benzodiazepines alprazolam, bromazepam, and clonazepam, as well as citalopram, gabapentin, and phenelzine. Pregabalin has also been shown to maintain benefits and prevent relapse in a six-month study.

Patients who do not respond to several medication trials and/or CBT are considered to have treatment-refractory illness. In such patients it is important to reassess the diagnosis and consider comorbid medical and psychiatric conditions that may be affecting response to therapy. Third-line agents and adjunctive therapies may be useful when patients fail to respond to optimal treatment trials of first- and second-line therapies used alone and in combination.

## Generalized anxiety disorder

### Epidemiology

The estimated 12-month prevalence of GAD ranges from 1-4%, and the lifetime prevalence is approximately 6% [2,3,16,503]. GAD is more frequent in Caucasians compared to other groups [504]. The usual age of onset varies and may be bimodal with the median age of onset being approximately 31 years [2] and mean age of onset being 32.7 years [505]. The prevalence of GAD is estimated to be 3% in children and 10.8% in adolescents [506], with the age of onset for children and adolescents being between ages 10 and 14 [507]. Some data suggest that women may be two to three times more likely to suffer from GAD than men [16,508], and GAD may be more common in older adults [509,510]. This disorder is reportedly frequently under-recognized with less than one-third of patients being adequately treated [511,512]. This is further complicated in children because of the previous designation of Overanxious Disorder of Childhood and its possible differentiation of childhood GAD from GAD in adults.

GAD is associated with functional [15,511,513], occupational [511], and QoL impairments [16,511], as well as substantial economic costs [511,514]. In addition, in primary care 60-94% of patients with GAD report painful physical symptoms [515,516], and these were the main reason for initial presentation to a physician in 72% of cases [516].

#### Comorbidity

GAD is associated with high rates of comorbid psychiatric conditions including other anxiety or related disorders and MDD [16]. The risk of medical conditions is also elevated [16], including pain syndromes [16,517], hypertension [16], as well as cardiovascular and gastric conditions [16,518]. The presence of comorbid depression increases the severity of illness, functional impairment [519], and economic costs [514].

### Diagnosis

GAD is characterized by excessive anxiety and worry about multiple events or activities such as school or work difficulties, which is apparent on a majority of days over the previous six months (Table 22) [26]. In addition, GAD is associated with restlessness, muscle tension, fatigue, concentration difficulties, irritability, and sleep issues [26].

**Table 22:** DSM-5 diagnosis of GAD

• Excessive anxiety and worry (apprehensive expectation) about a number of events or activities (e.g., school/work performance)
• The individual finds it difficult to control the worry
• Excessive anxiety and worry are associated with ≥3 of the following symptoms (with at least some occurring more days than not for ≥6 months): ○ Restlessness or feeling keyed-up or on edge, being easily fatigued, difficulty concentrating, irritability, muscle tension, or sleep disturbance
• The disturbance causes clinically significant distress or functional impairment

Adapted from DSM-5 [26].

The diagnostic criteria for GAD underwent one minor revision in the DSM-5 [26] compared to the DSM-IV-TR [144], the requirement that the disturbance not occur exclusively during a mood, psychotic, or pervasive developmental disorder was removed. However, it remains important to note that most of the treatment data described within this section are based on patients meeting DSM-IV criteria (or older).

### Psychological treatment

Meta-analyses clearly demonstrate that CBT significantly reduces GAD symptoms and is markedly more effective than placebo or wait-list control conditions for GAD (Level 1) [55,64,65,70,520]. Few studies have compared CBT and pharmacotherapy alone in the same trial, but the magnitude of benefits appear to be comparable for both groups [521-523]. Individual and group therapy appear to be equally effective in terms of anxiety symptom reduction, but individual therapy may lead to earlier improvement in worry and depression symptoms [65,520].

The intensity of therapy was assessed in a meta-analysis of 25 studies [65]. Regimens including fewer than eight sessions were as effective as those of eight or more for anxiety symptoms, but the more intense regimens were more effective in improving symptoms of worry and depression compared with fewer sessions [65].

Several studies have demonstrated the utility of internet-based or computer-based CBT programs [79,524-526]. ICBT has been shown to be significantly more effective than wait-list control [79,524,525], with benefits being maintained at long-term follow-up [525]. In addition, a peer-to-peer cognitive self-therapy program was as effective as treatment-as-usual, with a decreased need for therapist contact [527].

A meta-analysis of five trials found no significant differences between CBT and relaxation therapy [55]. However, more recent studies suggest that applied relaxation has limited efficacy [528-530]. One RCT found little evidence that patients with GAD can learn to relax in therapy or that a decrease in activation is associated with a reduction in anxiety [529]. Balneotherapy, a relaxation therapy involving spa-related treatments, demonstrated potential advantages over SSRI pharmacotherapy in improving anxiety scores and response rates in patients with GAD in a large RCT [531]; however, while this study may be interesting, concerns pertaining to blinding and potential bias indicate further study is needed [531].

Several research-based variables have been specifically identified among individuals with GAD in order to generate evidence-based CBT protocols for GAD, including: intolerance of uncertainty, poor problem-solving confidence, as well as positive and negative metacognitive beliefs about the function or utility of worry [532]. Specific psychotherapeutic protocols based upon models of the disorder that target variables underlying GAD have been developed to individualize therapy. Acceptance-based behavior therapy [533], meta-cognitive therapy [528,534], CBT targeting intolerance of uncertainty [530], and adjunctive MBCT [184] have demonstrated efficacy for the treatment of GAD. Targeting worry and relaxation [535], as well as looming vulnerability (the tendency to generate and maintain internal scenarios of increasing risk and danger) [536], may also be beneficial.

Psychodynamic therapy may also be of benefit, however the research findings to date are unclear. A RCT found that short-term psychodynamic psychotherapy was as effective as CBT in improving anxiety scores, but CBT was superior on measures of worry and depression [537]. Another study found no significant differences between brief psychodynamic therapy, pharmacotherapy, or the combination [523].

No significant benefits were found with the addition of interpersonal and emotional processing therapy to CBT when compared with CBT plus supportive listening [538]. However, pretreatment motivational interviewing as an adjunct to CBT was shown to help reduce resistance to therapy, improve homework compliance, and improve worry outcomes — this strategy may be particularly useful in more severe cases [539,540].

In clinical practice, the approach may need to be individualized to the problems experienced by the patient.

#### Psychological and pharmacological treatment

Few data are available on the use of combined psychological and pharmacological treatment. A meta-analysis concluded that combination pharmacotherapy and CBT was more effective than CBT alone at posttreatment but not at six-month follow-up [83]. While large effect sizes were found for GAD, data were available from only two studies, and these compared CBT plus diazepam or buspirone with CBT alone [83]. Compared to pharmacotherapy alone, the few studies that have assessed the benefits of adjunctive psychotherapy have been conflicting [184,523,541,542]. One study suggested benefits of the combination [184], while two other studies did not [523,541]. However, adjunctive CBT was shown to facilitate benzodiazepine tapering in patients with GAD [542].

There is no current evidence to support the routine combination of CBT and pharmacotherapy. However, as in other anxiety and related disorders, when patients do not benefit from CBT or have a limited response, a trial of pharmacotherapy is advisable. Similarly, patients who show limited benefit from pharmacotherapy may benefit from CBT.

#### Long-term effects of psychological treatment

Long-term follow-up data from a meta-analysis [520] and RCTs [523,525,535,543] suggest that benefits of psychological treatments are maintained at one to three years follow-up after treatment.

### Pharmacological treatment

The management of patients with GAD should follow the principles discussed in Section 2. Pharmacological interventions that have good evidence for efficacy in treating GAD include SSRIs, SNRIs, TCAs, benzodiazepines, pregabalin, quetiapine XR, and other therapies. Treatments that have been investigated for use in GAD have been assessed according to the criteria for strength of evidence (Tables 1 and 2) and are summarized in Tables 23 and 24.

**Table 23:** Strength of evidence for pharmacotherapy for GAD

Agent	Level of evidence	Agent	Level of evidence
***Antidepressants***			

**SSRIs**		**TCAs**	
Escitalopram [544-552]	1	Imipramine [553,581-583]	1
		
Paroxetine [546,547,553-558]	1	**Other antidepressants**	
Sertraline [556,559-561]	1	Agomelatine [584,585]	1
Citalopram [562]	3	Vortioxetine [586,587]	1*
Fluoxetine [563]	3	Bupropion XL [549]	2
Paroxetine CR [564,565]	3	Trazodone [583]	2
**SNRIs**		Mirtazapine [588]	3
Duloxetine [566-571]	1		
Venlafaxine XR [548,553,570-580]	1		

***Other therapies***			

**Anxiolytics**		**Atypical antipsychotics**	
** Benzodiazepines**		Quetiapine XR [551,557,602,603]	1
Alprazolam [589-593]	1	Adjunctive quetiapine [565,604,605]	1*
Bromazepam [589,594]	1	Adjunctive risperidone [606,607]	1*
Diazepam [583,589,595,596]	1	Adjunctive olanzapine [608]	2
Lorazepam [589,593,597-601]	1	Adjunctive aripiprazole [269,609]	3
		Adjunctive quetiapine XR [610]	3
		Adjunctive or monotx ziprasidone [611,612]	2 (-ve)

**Anticonvulsants**		**Other treatments**	
Pregabalin [576,577,592,593,597,613]	1	Buspirone [108,561,572,589,598,618,619]	1
Divalproex chrono [614]	2	Hydroxyzine [594,619,620]	1
Tiagabine [615,616]	1 (-ve)	Pexacerfont [552]	2 (-ve)
Adjunctive pregabalin [617]	2	Propranolol [621]	2 (-ve)
		Memantine [622]	4 (-ve)

*Conflicting data. SNRI = serotonin–norepinephrine reuptake inhibitor; SSRI = selective serotonin reuptake inhibitor; TCA = tricyclic antidepressant; XL = extended release; XR=extended release; (-ve) = negative.

**Table 24:** Recommendations for pharmacotherapy for GAD

First-line	Agomelatine, duloxetine, escitalopram, paroxetine, paroxetine CR, pregabalin, sertraline, venlafaxine XR
**Second-line**	Alprazolam^*^, bromazepam^*^, bupropion XL*, buspirone, diazepam^*^, hydroxyzine, imipramine, lorazepam^*^, quetiapine XR*, vortioxetine

**Third-line**	Citalopram, divalproex chrono, fluoxetine, mirtazapine, trazodone

**Adjunctive therapy**	**Second-line:** pregabalin **Third-line:** aripiprazole, olanzapine, quetiapine, quetiapine XR, risperidone **Not recommended:** ziprasidone

**Not recommended**	Beta blockers (propranolol), pexacerfont, tiagabine

CR = controlled release; XL = extended release; XR=extended release.

*Note: These have distinct mechanisms, efficacy and safety profiles. Within these second-line agents, benzodiazepines would be considered first in most cases, except where there is a risk of substance abuse, while bupropion XL would likely be reserved for later. Quetiapine XR remains a good choice in terms of efficacy, but given the metabolic concerns associated with atypical antipsychotic, it should be reserved for patients who cannot be provided antidepressants or benzodiazepines. Please refer to text for further rationale for the recommendations.

#### First-line agents

*Antidepressants* (*SSRIs & SNRIs*)*:* Evidence from RCTs supports the use of SSRIs including escitalopram [544-552] and sertraline [556,559-561], as well as the SNRIs duloxetine [566-571] and venlafaxine XR [548,553,570-580] (all Level 1) for the first-line treatment of GAD. Similar evidence exists for paroxetine [546,547,553-558] supporting its use as a first-line option. Paroxetine CR has a similar active ingredient, and although there are less data supporting its use, it is likely interchangeable with paroxetine as a first-line agent (Level 3) [564,565]. In head-to-head comparisons, the efficacy of SSRIs and SNRIs appear to be similar [546,547,549,556,558,570,571]. Some data suggest that escitalopram may be less effective than venlafaxine XR [548] or quetiapine XR [551]. Efficacy of venlafaxine was similar to pregabalin in one RCT [576], but less effective in another [577].

*Other antidepressants:* In two 12-week, double-blind RCTs, agomelatine was found to be more effective than placebo (Level 1) [584,585], and as effective as escitalopram [585].

*Pregabalin:* The anticonvulsant pregabalin was more effective than placebo in RCTs [576,577,592,593,597,613] and as effective as benzodiazepines [592,593,597] in patients with GAD (Level 1). Pregabalin was more effective than venlafaxine XR in one RCT [577], but equivalent in another [576].

#### Second-line agents

*Benzodiazepines:* Alprazolam [589-593], bromazepam [589,594], diazepam [583,589,595,596], and lorazepam [589,593,597-601] all have demonstrated efficacy for the treatment of GAD (all Level 1). While these agents have level 1 evidence for efficacy, they are recommended as second-line therapy, and usually only for short-term use, because of side effects, dependence, and withdrawal issues.

*TCAs and other antidepressants:* In RCTs, imipramine was superior to placebo and as effective as benzodiazepines for the treatment of GAD (Level 1) [553,581-583]. However, because of side effects and potential toxicity in overdose, imipramine is recommended as a second-line option. While there are little data on bupropion XL (Level 2), in a 12-week RCT in patients with GAD it was as effective as escitalopram (a first-line option), supporting its use as a second-line option [549].

Vortioxetine is a so-called "serotonin modulator" because of its activity in a variety of serotonin receptors. Results from two similar, eight-week, placebo-controlled RCTs with vortioxetine were conflicting, with one trial being positive [587] and the other negative (Level 1, conflicting) [586]. The differences in outcomes may be related to differences in recruitment between the two studies [623], and data suggest that vortioxetine may be useful in GAD.

*Quetiapine XR:* There is good evidence for the efficacy of quetiapine XR for the management of GAD (Level 1) [551,557,602,603]. Two meta-analyses [115,116] concluded that quetiapine was significantly superior to placebo and equivalent to antidepressants [115] for the treatment of GAD. However, quetiapine was associated with more weight gain and sedation, and higher dropout rates due to adverse events compared with placebo or antidepressants [115,116]. Due to tolerability and long-term safety concerns with atypical antipsychotics, this treatment is recommended as a second-line option for patients who cannot be provided antidepressants or benzodiazepines.

*Other treatments:* Buspirone was more effective than placebo and as effective as benzodiazepines in several RCTs (Level 1) [108,561,572,589,598,618,619]. There are limited data comparing buspirone to antidepressants, with it being less effective than venlafaxine XR in one study [572], but as effective as sertraline in another [561]. Limited effectiveness in clinical practice relegates buspirone to a second-line agent.

Hydroxyzine has demonstrated efficacy superior to placebo and similar to benzodiazepines and buspirone in RCTs (Level 1) [594,619,620]; however, clinical experience with this agent in the treatment of GAD remains limited.

#### Third-line agents

The following agents are recommended as third-line options because of limited data, side effects, or lack of clinical experience as a primary therapy for the treatment of GAD.

*Antidepressants:* In open-label studies or case series, the antidepressants citalopram [562], fluoxetine [563], paroxetine CR [564,565], and mirtazapine [588] have demonstrated efficacy in patients with GAD (all Level 3). In a RCT, trazodone was as effective as diazepam (Level 2) [583].

*Other treatments:* Divalproex chrono was superior to placebo for the treatment of GAD (Level 2) [614], however this formulation is not widely available.

#### Adjunctive therapy

Adjunctive strategies have generally been studied in patients who have had an inadequate response to SSRI therapy, and can be considered for patients with treatment-resistant GAD.

*Second-line adjunctive therapies:* Adjunctive pregabalin demonstrated good efficacy in a large RCT in patients with GAD who had an inadequate response to prior treatments (Level 2) [617].

*Third-line adjunctive therapies:* A meta-analysis of five RCTs of adjunctive atypical antipsychotics found no significant improvement in response rates but higher discontinuation rates versus placebo in patients with refractory GAD [116].

Two RCTs suggest that adjunctive risperidone (Level 1, conflicting) [606,607] may be useful in some patients, but in the larger RCT it demonstrated superiority over placebo only in patients with moderate to severe residual symptoms at baseline [607]. Similarly, data on adjunctive quetiapine have been inconsistent (Level 1, conflicting) [565,604,605], with one RCT being negative [565], while another, unblinded RCT showed some, but limited benefits [605]. Adjunctive olanzapine demonstrated efficacy in a small RCT in patients who remained symptomatic after six weeks of SSRI therapy [608]. Adjunctive treatment with quetiapine XR [610] or aripiprazole [269,609] (both Level 3) also had some benefit in open trials.

Because of the limited evidence for efficacy and their potential for weight gain and metabolic side effects, atypical antipsychotics should be reserved for highly treatment-refractory cases of GAD, and other than quetiapine XR, used only as an adjunctive treatment.

*Not recommended adjunctive therapies:* Ziprasidone does not appear to be effective as adjunctive therapy (Level 2, negative) [611].

#### Not recommended

Propranolol [621] and pexacerfont [552] (both Level 2, negative) have not demonstrated efficacy and are not recommended in the treatment of GAD. While a small randomized, open-label trial suggested that tiagabine was as effective as paroxetine, the results of three placebo-controlled RCTs do not support the efficacy of tiagabine in patients with GAD (Level 1, negative) [615,616]. Memantine also does not appear to be effective in this disorder (Level 4, negative) [622].

#### Maintenance pharmacological treatment

Long-term therapy has been evaluated in relapse prevention and naturalistic follow-up studies. Relapse-prevention studies are those in which responders to SSRI therapy are randomized to continued active treatment or placebo. A meta-analysis of three relapse prevention studies included 1342 patients with GAD and found a highly significant reduction in relapse rates with continued SSRI treatment compared with placebo over six to 12 months (odds ratio for relapse was 0.20) [497].

In RCT discontinuation studies, duloxetine [624], escitalopram [625], paroxetine [626], and venlafaxine XR [627] have demonstrated significantly lower relapse rates over six to 18 months in the range of 10-20% with active treatment compared to 40-56% with placebo. Pregabalin [628] and quetiapine XR [629] have also demonstrated significantly lower relapse rates over six to 12 months of continued treatment in discontinuation trials.

In long-term RCT studies, escitalopram [546], paroxetine [546], and venlafaxine XR [578,579] have demonstrated continued improvement compared with placebo over approximately six months.

### Biological and alternative therapies

In general, these therapies may be useful for some patients; however, more data are needed.

*Biological therapies:* In a small open trial, rTMS was effective as monotherapy or as an adjunct to SSRIs in patients with GAD (Level 3) [630], and improvements were largely maintained six months after treatment [631].

*Alternative therapies:* Several herbal preparations have demonstrated efficacy comparable to lorazepam for the treatment of GAD including silexan (lavender oil) (Level 1) [600,632] and *Galphimia glauca* extract (Level 2) [601]. Cochrane meta-analyses found two studies of passiflora (passion flower) indicating it was as effective as benzodiazepines (Level 2) [633], and one study of valerian which found no significant differences between placebo, valerian, or diazepam (Level 2, negative) [634,635]. Unfortunately, because these preparations are poorly standardized and have substantial variation in proportion of the active ingredient in different products, they cannot be widely recommended.

A RCT of adjunctive resistance training (weightlifting) or aerobic exercise found significant symptomatic improvements compared to a wait-list condition (Level 2) [636]. A systematic review included four studies of acupuncture in GAD or anxiety neurosis, and while all trials reported positive findings, methodological details were lacking and the authors concluded that there was insufficient evidence to determine efficacy (Level 2) [637]. Open-label studies suggest that adjunctive meditation and yoga-based treatments may be useful in patients with GAD (Level 3) [638,639].

*Not recommended alternative therapy:* In a RCT, there were no significant improvements with bright light therapy compared with placebo (Level 2, negative) [640], and this treatment is not recommended.

### Summary

The lifetime prevalence of GAD is approximately 6%, it is more frequent in women than in men, with age of onset reflecting a bimodal distribution (onset in late-teens to early-twenties, and again in the 30s and 40s). GAD is associated with substantial functional impairment and a high prevalence of comorbid psychiatric and medical disorders. According to DSM-5 criteria, GAD is characterized by excessive anxiety and worry about multiple situations and is associated with restlessness, muscle tension, and behavioral changes.

CBT is an effective first-line option for the treatment of GAD and is as effective as pharmacotherapy. Internet-based and computer-based CBT have also demonstrated efficacy. Evidence does not support the routine combination of CBT and pharmacotherapy, but when patients do not benefit from CBT, a trial of pharmacotherapy is advisable, and vice versa.

Pharmacotherapeutic approaches should begin with one of the first-line options including an SSRI such as escitalopram, paroxetine, or sertraline, an SNRI such as duloxetine or venlafaxine XR, or other antidepressant such as agomelatine. The anticonvulsant pregabalin is also a recommended first-line therapy.

If response to optimal doses is inadequate or the agent is not tolerated, therapy should be switched to another first-line agent before considering second-line medications. Second-line choices include bupropion XL, buspirone, hydroxyzine, imipramine, quetiapine XR, vortioxetine, as well as the benzodiazepines, alprazolam, bromazepam, diazepam, and lorazepam.

Patients who do not respond to multiple courses of therapy are considered to have treatment-refractory illness. In such patients it is important to reassess the diagnosis and consider comorbid medical and psychiatric conditions that may be affecting response to therapy. Third-line agents, adjunctive therapies, as well as biological and alternative therapies may be useful when patients fail to respond to an optimal treatment trial of first- and second-line therapies used alone and in combination.

## Obsessive-compulsive disorder

### Epidemiology

OCD is a relatively uncommon, yet severe, mental disorder, with an estimated lifetime and 12-month prevalence of 1.0-2.3% and 0.7%-1.2% in adults, respectively [2,3,641,642]. Mean age of onset of OCD is ~20 years of age, but symptoms can occur below the age of 10, with few new cases after the early 30s [2,641,643]. Rates of treatment-seeking have been estimated to be only about 14-56% of patients, suggesting that OCD may be under-recognized and under-treated [644,645]. Social isolation, history of physical abuse, and negative emotionality are risk factors for the development of OCD [646].

OCD is associated with a substantial negative impact on QoL for both patients [647,648] and their caregivers [649]. Patients experience cognitive, social, and occupational impairments [642,645,650,651]. In addition, up to one-quarter of patients with OCD have attempted suicide [645,652]. OCD symptoms are associated with increased rates of health care utilization compared to those without OCD symptoms [642], with health care costs estimated at $10.6 billion/year (2005) in the US [653].

#### Comorbidity

About 60-90% of patients with OCD also have a comorbid disorder [641,645]. Patients with OCD or OCD symptoms have a three-times higher rate of comorbidity compared to those without OCD symptoms [642]. Common comorbidities include mood, anxiety, and somatoform disorders, as well as SUDs, psychotic disorders, and bipolar disorders [641,642,645].

### Diagnosis

A diagnosis of OCD requires the presence of obsessions and/or compulsions (Table 25) [26]. Obsessions are defined as recurrent, persistent, and intrusive thoughts, images, or urges that cause marked anxiety, and compulsions are defined as repetitive behaviors or mental acts that the patient feels compelled to perform to reduce the obsession-related anxiety [26]. The obsessions or compulsions are time consuming and cause significant impairment in social or occupational functioning.

**Table 25:** DSM-5 diagnosis of OCD

• Presence of either obsessions, compulsions, or both ○ Obsessions are defined by the following: • Recurrent and persistent thoughts, urges, or images that are experienced as intrusive and unwanted and that cause marked anxiety or distress • The individual attempts to ignore or suppress such thoughts, urges, or images, or to neutralize them with other thoughts or actions ○ Compulsions are defined by the following: • Repetitive behaviors (e.g., hand washing, ordering, checking) or mental acts (e.g., praying, counting, repeating words silently) that the individual feels driven to perform in response to an obsession or according to rigid rules • Compulsions are aimed preventing or reducing anxiety or preventing some dreaded situation or event; however, they are not connected in a realistic way with what they are designed to neutralize or are clearly excessive
• The obsessions or compulsions are time-consuming (e.g., take >1 h/day) or cause clinically significant distress or functional impairment
• Specify patient’s degree of insight as to reality of OCD beliefs: ○ Good or fair insight (i.e., definitely or probably not true) ○ Poor insight (i.e., probably true) ○ Absent insight (i.e., completely convinced beliefs are true)
• Specify if "tic-related" OCD

Adapted from DSM-5 [26].

In the DSM-5, OCD has been moved from the “anxiety disorders” [144] to a new diagnostic category called “obsessive-compulsive and related disorders.” In addition to OCD, this new category also includes diagnostic criteria for body dysmorphic disorder, hoarding disorder, hair-pulling disorder (trichotillomania), and skin picking disorder [26].

Most of the other modifications to the OCD diagnostic criteria in the DSM-5 were minor wording changes designed to enhance clarity or further operationalize concepts that were considered too vague [26]. In particular, the definitions of obsessions and compulsions were clarified and simplified [26,654]. The requirement that the patient recognizes that the obsessions or compulsions are “excessive or unreasonable” has been deleted, since these terms are subject to interpretation and patients can have varying levels of insight. As a result, the previous DSM-IV-TR specifier of “poor insight” has been expanded to include: good or fair, poor, and absent insight [26]. Finally, a specifier of "tic-related" OCD has been added [26].

While the most up-to-date DSM-5 diagnostic criteria are presented here, it is important to note that most of the treatment data described within this section are based on patients meeting DSM-IV criteria (or older).

### Psychological treatment

Meta-analyses support the beneficial effects of psychological treatment for OCD, mainly CBT, generally including exposure with response prevention (ERP) [60-63,70,71,655-657]. CBT is equivalent or superior to pharmacotherapy [71,658-660]. Results with CBT were generally similar in comparisons of interventions with an emphasis on ERP and those with an emphasis on cognitive elements [60,63,655]. A treatment specifically designed to address fear of contamination with infectious substances, using a cognitive intervention that includes no direct exposure (“danger ideation reduction therapy, DIRT”), was found to be more efficacious than ERP [661,662]. Cognitive interventions may be important in patients who do not have overt compulsions, which can make ERP more difficult. One meta-analysis found that exposure *in vivo* combined with imaginal exposure was better than exposure *in vivo* alone [60].

Several meta-analyses have demonstrated no significant differences in efficacy between group and individual CBT [60,62,663]. However, results of head-to-head trials are conflicting, with some RCTs finding no significant differences in efficacy between group and individual therapy [663,664], and others showing individual therapy to be superior [665-667]. Differences in results may be explained by the fact that in individual therapy the therapist may have the advantage of being more aware of the patient’s dysfunctional beliefs, however, the group therapy setting may offer the advantages of group encouragement, reciprocal support, imitation, and interpersonal learning which may result in an increased motivation and reduced discontinuation of treatment [62].

An important practical question concerns the intensity and duration of treatment. The intensive ERP program described by Foa’s group involves 15 two-hour sessions scheduled five days a week over three weeks [658,668]. A similar program administered twice-weekly (a more practical approach for many patients and therapists) was as effective at the end of follow-up as the intensive five-days/week strategy [669]. A step-care approach in which patients received six weeks of low-intensity counseling with ERP bibliotherapy followed by standard ERP for non-responders only was found to be as effective as initial therapy with standard ERP (17 sessions twice weekly), but was significantly less costly [670].

Other techniques that may be useful include acceptance and commitment therapy (ACT) [671], modular cognitive therapy (CT) addressing OCD beliefs [672,673], CT addressing obsessional doubt [674], organizational training [675,676], and mindfulness training [677]. RCTs on the benefits of adding motivational interviewing to CBT have been conflicting, with one showing no additional benefits [678], while another demonstrated improved symptom reduction and remission rates compared with CBT alone [679]. While EMDR was more effective than an SSRI in a RCT [680], data are limited and this technique is not generally recommended for patients with OCD.

Data suggest that therapist-guided exposure is better than self-exposure [60]. While both treatment conditions showed significant symptom reduction, therapist-administered ERP was superior to self-administered ERP in improving OCD symptoms and self-reported functional impairment [681]. Other data suggest that ERP delivered by telephone is equivalent to face-to-face ERP [682]. Bibliotherapy in the form of self-help manuals delivered to patients via email has demonstrated significantly greater improvements in OCD symptoms compared with wait-list control groups in two RCTs [683,684].

ICBT is an easily accessible treatment that has the potential to reach untreated patients and motivate them for face-to-face psychotherapy if necessary [684,685]. Several RCTs have demonstrated that ICBT programs are significantly more effective than supportive therapy or relaxation control strategies [685-687]. ICBT was as effective as therapist-led CBT only when patients completed at least one self-exposure session [687]. ICBT was associated with significantly better outcomes when it included brief, scheduled, therapist-initiated telephone support compared with on-demand phone support [688].

Family accommodation (i.e., family members taking part in the performance of rituals, avoidance of anxiety-provoking situations, or modification of daily routines to assist a relative with OCD) has been associated with poorer response to both behavioral and pharmacological treatments [689]. Clinicians may want to consider targeting family accommodation in order to improve treatment outcomes for some patients.

Although hoarding disorder is now a separate diagnosis [690], the limited data available on the treatment of hoarding will be mentioned in this section on OCD. One RCT found that group CBT significantly reduced hoarding and depression symptoms while bibliotherapy alone was associated with very limited improvements [691]. The addition of posttreatment, nonclinician, home assistance did not significantly improve outcomes.

#### Combined psychological and pharmacological treatment

The combination of psychological and pharmacological treatment has been shown to be superior to medication alone [657,658,692-694], but not to CBT alone [83,658,692,694,695]. These findings suggest that if pharmacotherapy is required or preferred, adding CBT to pharmacological treatment of OCD may enhance response rates and reduce relapse rates. Unlike in some anxiety and related disorders, there does not appear to be any contraindication to combining CBT with medications in patients with OCD [696], and combined treatment may improve relapse prevention [697].

Adding d-cycloserine may hasten the onset of improvements with ERP, with significant benefits over placebo during the first four or five ERP sessions [698-700], but this effect has not been seen in all studies [701].

#### Long-term effects of psychological treatment

Follow-up studies suggest that the benefits of CBT are maintained at one to five years of follow-up [664,695,702-704].

### Pharmacological treatment

The management of patients with OCD should follow the principles discussed in Section 2. SSRIs are recommended first-line pharmacological interventions for OCD, while SNRIs, clomipramine, and other antidepressants are recommended second- and third-line treatments. Treatments that have been investigated for use in OCD have been assessed according to the criteria for strength of evidence (Tables 1 and 2) and are summarized in Tables 26 and 27.

**Table 26:** Strength of evidence of pharmacotherapy for OCD

Agent	Level of evidence	Agent	Level of evidence
***Antidepressants***			

**SSRIs**		**MAOIs**	
Escitalopram [705-709]	1	Phenelzine [737,738]	2*
Fluoxetine [660,710-716]	1	Tranylcypromine [739]	4
		
Fluvoxamine [711,713,714,717-719]	1	**TCAs**	
Paroxetine [705,720-722]	1	Clomipramine [658,711,713,714,716-718,720,724,740,741]	1
Sertraline [659,710,711,713,714,723-725]	1	IV clomipramine [742-744]	2
Citalopram [680,726-728]	2	Desipramine [723,745]	2 (-ve)
IV citalopram [729]	3	Adjunctive clomipramine [746,747]	2 (-ve)
		
Adjunctive citalopram [730]	3	**Other antidepressants**	
**SNRIs**		Mirtazapine [748]	2
Venlafaxine XR [721,731-733]	2	Bupropion [749]	3 (-ve)
Duloxetine [734-736]	4	Adjunctive mirtazapine [727]	3

***Other therapies***			

**Antipsychotics**		**Anxiolytics**	
Adjunctive aripiprazole [750-755]	1	** Benzodiazepines**	
Adjunctive risperidone [755-761]	1*	Clonazepam [771]	2 (-ve)
Adjunctive olanzapine [760,762,763]	1*	Adjunctive clonazepam [772]	2 (-ve)
		
Adjunctive quetiapine [728,746,747,764-768]	1*	**Other treatments**	
Adjunctive haloperidol [758,769]	2	Clonidine [773]	2 (-ve)
Adjunctive amisulpride [770]	3	Adjunctive pindolol [774-776]	1*
Adjunctive ziprasidone [767]	4	Adjunctive celecoxib [777]	2
		
**Anticonvulsants**		Adjunctive granisetron [778]	2
Adjunctive topiramate [795-798]	1*	Adjunctive IV ketamine [779,780]	2
Adjunctive lamotrigine [799,800]	2	Adjunctive memantine [622,781-783]	2
Adjunctive pregabalin [801,802]	3	Adjunctive ondansetron [784,785]	2
Adjunctive gabapentin [803,804]	3 (-ve)	Adjunctive N-acetylcysteine [786,787]	2
		
**Opioids**		Adjunctive riluzole [788,789]	3
Tramadol [805,806]	4	Adjunctive lithium [790,791]	1 (-ve)
Naltrexone [807]	3 (-ve)	Adjunctive buspirone [792,793]	2 (-ve)
Adjunctive morphine [808]	2	Adjunctive minocycline [794]	4 (-ve)

*Conflicting data. IV = intravenous; MAOI = monoamine oxidase inhibitor; SNRI = serotonin–norepinephrine reuptake inhibitor; SSRI = selective serotonin reuptake inhibitor; TCA = tricyclic antidepressant; XR = extended release; (-ve) = negative.

**Table 27:** Recommendations for pharmacotherapy for OCD

First-line	Escitalopram, fluoxetine, fluvoxamine, paroxetine, sertraline
**Second-line**	Citalopram, clomipramine, mirtazapine, venlafaxine XR

**Third-line**	IV citalopram, IV clomipramine, duloxetine, phenelzine, tramadol, tranylcypromine

**Adjunctive therapy**	**First-line:** aripiprazole, risperidone **Second-line:** memantine, quetiapine, topiramate **Third-line:** amisulpride, celecoxib, citalopram, granisetron, haloperidol, IV ketamine, mirtazapine, N-acetylcysteine, olanzapine, ondansetron, pindolol, pregabalin, riluzole, ziprasidone **Not recommended:** buspirone, clonazepam, lithium, morphine

**Not recommended**	Clonazepam, clonidine, desipramine

IV = intravenous; XR = extended release.

#### First-line agents

*SSRIs:* Evidence from RCTs and meta-analyses support the use of SSRIs, including escitalopram [705-709], fluoxetine [660,710-716], fluvoxamine [711,713,714,717-719], paroxetine [705,720-722], and sertraline [659,710,711,713,714,723-725] (all Level 1), in the treatment of OCD. In meta-analyses, response rates with SSRIs are generally twice those of placebo [809], at 40-60% with treatment versus <20% with placebo [711,713,714,740,741]. Pooled response rates are not significantly different between SSRIs [809]. In meta-analyses and head-to-head trials, compared with clomipramine, the SSRIs fluoxetine, fluvoxamine, paroxetine, and sertraline had similar efficacy but better tolerability [711,713,714,716-718,720,724].

Dimensional analyses have suggested that symmetry/hoarding symptoms may be associated with a poorer response to SSRI therapy [810,811], while aggressive/religious/sexual symptoms may predict better outcomes [810,812]. It has been hypothesized that the symmetry/hoarding symptom dimension may be mediated by the dopamine system and aggressive behaviors by the serotonin system [810,812].

#### Second-line agents

*Clomipramine:* There is good evidence to support the use of clomipramine in the treatment of OCD (Level 1) [658,711,713,714,716-718,720,724,740,741]. Clomipramine has efficacy similar to SSRIs, but SSRIs are generally better tolerated [711,713,714,716-718,720,724]. Side effects and safety are issues with clomipramine and therefore it is recommended as a second-line choice. Common adverse effects include anticholinergic effects such as dry mouth, constipation, and blurred vision, as well as urinary retention, orthostatic hypotension, weight gain, and sedation [813,814]. The major safety concerns are cardiac arrhythmias, seizures, drug interactions, and toxicity in overdose [813,814].

*Antidepressants:* In RCTs, citalopram was more effective than placebo but less effective than psychotherapy (Level 2) [680,726]. Additional data from augmentation studies support the efficacy of citalopram for the treatment of OCD [727,728]. However, given that other SSRIs have much stronger evidence, citalopram was designated a second-line option. The only RCT data on the use of mirtazapine in OCD are from a discontinuation study in which continued mirtazapine was associated with continued improvement (Level 2) [748]. There is some evidence to support the use of venlafaxine XR for the treatment of OCD (Level 2) [721,731-733]. In RCTs, venlafaxine XR was more effective than placebo [732], and as effective as paroxetine [721] and clomipramine [731]. In a double-blind extension of a RCT [721], paroxetine was more efficacious than venlafaxine in the treatment of non-responders to previous treatment with the alternate antidepressant [733].

#### Third-line agents

*Intravenous clomipramine:* In a RCT, intravenous (IV) clomipramine was more effective than placebo in patients with OCD (Level 2) [742]. Initiating therapy with IV then switching to oral therapy does not appear to be associated with greater benefit compared with oral therapy alone [743,744].

*Other agents:* IV citalopram [729] (Level 3), as well as duloxetine [734-736], tramadol [805,806], and tranylcypromine [739] (all Level 4) have demonstrated some efficacy in open trials or case reports. Results with phenelzine have been inconsistent. In one RCT, phenelzine was not significantly better than placebo [738], but in another it was as effective as clomipramine (Level 2) [737]. In the placebo-controlled trial, post-hoc analysis suggested that phenelzine may be beneficial in patients with symmetry or other atypical obsessions [738].

These agents are recommended as third-line options, and may be useful in refractory patients after first- and second-line monotherapies and adjuncts have been unsuccessful.

#### Adjunctive therapy

Adjunctive strategies have generally been studied in patients who have had an inadequate response to SSRI therapy, and can be considered for patients with treatment-resistant OCD. A meta-analysis demonstrated that response rates with adjunctive medication were twice those of placebo, however these were still quite low (31.8% versus 13.6%) [815]. Meta-analyses of RCTs found that adding risperidone (and possibly quetiapine) to antidepressants increased efficacy but decreased tolerability, while adjunctive olanzapine did not improve response rates [816,817].

*First-line adjunctive therapies:* In RCTs, adjunctive aripiprazole was significantly more effective than placebo (Level 1) [750,754], and may be as effective as risperidone [755]. Additional open-label data also support the beneficial effects of adjunctive aripiprazole [751-753].

As adjunctive therapy for treatment-resistant OCD, risperidone was more effective than placebo (Level 1) [756-759] and as effective as olanzapine [760] and aripiprazole overall [755]. Compared with aripiprazole, risperidone may provide greater improvement in obsessions [755]. Risperidone was also as effective as haloperidol for obsessions, but less so for compulsions, however it was better tolerated [758]. More recently an open, randomized study found that while augmentation with ERP was superior to risperidone or pill placebo, risperidone was not significantly more effective than placebo [761]. However, patients in this study had some response to SSRI therapy and may have been less refractory compared to those in other studies. Considering the tolerability concerns of atypical antipsychotics, these data reinforce that this augmentation strategy should be reserved for patients with treatment-resistant OCD.

*Second-line adjunctive therapies:* RCT evidence demonstrated that adjunctive memantine was superior to placebo (Level 2) [783]. Additional open-label data also support this therapy [622,781,782]. Another option which may be useful as an adjunctive therapy in those with refractory OCD is the atypical antipsychotic quetiapine (Level 1, conflicting) [728,746,747,764-766,768].

Data from small RCTs suggest that topiramate may be a useful adjunctive therapy, but data are conflicting (Level 1) [796,797]. In one RCT, adjunctive topiramate significantly improved Yale-Brown Obsessive Compulsive Scale (Y-BOCS) scores compared with placebo [797], while in another trial, adjunctive topiramate significantly improved compulsions but not obsessions [796]. Additional open-label data support the use of adjunctive topiramate [795,798].

*Third-line adjunctive therapies:* The agents discussed below are recommended as third-line adjunctive options, since some data are available to suggest they may be useful but there is conflicting or inadequate evidence to warrant stronger recommendations. These agents may be useful for some patients, but more data are needed.

Other atypical antipsychotics have been assessed as adjunctive therapies in patients with refractory OCD, including olanzapine (Level 1, conflicting) [760,762,763], amisulpride (Level 3) [770], and ziprasidone (Level 4) [767].

There is level 2 evidence to support the use of adjunctive haloperidol in patients with refractory OCD [758,769], and although it may be as effective as adjunctive risperidone, it is a third-line choice because it was less well tolerated [758].

Adjunctive mirtazapine was associated with an earlier onset of response of OCD symptoms compared with citalopram alone, but there was no advantage of the combination over time (Level 2) [727]. Some data also support the efficacy of adjunctive citalopram for treatment-resistant OCD (Level 3) [730].

Adjunctive anticonvulsants may be useful for some patients with refractory illness [799-802]. In a small RCT, adjunctive lamotrigine improved both obsessions and compulsions compared to SSRI therapy (Level 2) [799]. Open-label data also suggest that adjunctive pregabalin may be useful (Level 3) [801,802].

Other agents that have been studied as adjunctive therapy for treatment-resistant OCD include celecoxib [777], granisetron [778], IV ketamine [779,780], ondansetron [784,785], N-acetylcysteine [786,787] (all Level 2), and riluzole (Level 3) [788,789]. There is little clinical experience with these agents for refractory OCD, therefore they are recommended as third-line adjunctive options only.

Results with pindolol augmentation have been inconsistent, with significant improvements in one small RCT [774], but not in other randomized or open trials (Level 1, conflicting) [775,776].

In two randomized, quetiapine-controlled trials, adjunctive clomipramine was not superior to SSRI therapy (Level 2, negative) [746,747]. Clinical experience suggests that some patients may benefit from adjunctive clomipramine; however, plasma levels should be monitored because of the risk of drug interactions with SSRIs [747,813].

#### Not recommended

Clonazepam [771], clonidine [773], and desipramine (all Level 2, negative) [723,745] have not demonstrated efficacy and are not recommended in the treatment of OCD. Bupropion [749] and naltrexone (both Level 3, negative) [807] also do not appear to be effective in this disorder.

Adjunctive buspirone [792,793], clonazepam [772] (Level 2, negative), or lithium [790,791] (Level 1, negative) have not demonstrated efficacy for the treatment of OCD. There is currently no evidence for the efficacy of adjunctive gabapentin (Level 3, negative) [803,804] or minocycline (Level 4, negative) [794], but there are insufficient data to make recommendations at this time. In a RCT, adjunctive once-weekly oral morphine was effective in patients who had failed six SSRI trials (Level 2) [808], however, morphine is not generally recommended because of its potential for abuse.

#### Maintenance pharmacological treatment

Long-term therapy has been evaluated in relapse prevention and naturalistic follow-up studies. Relapse-prevention studies are those in which responders to SSRI therapy are randomized to continued active treatment or placebo. A meta-analysis of six relapse prevention studies included 951 patients with OCD and found a highly significant reduction in relapse rates with continued SSRI treatment compared with placebo over six to 12 months (odds ratio for relapse was 0.38) [497]. In RCTs, escitalopram [818], paroxetine [722], sertraline [819], and high-dose fluoxetine [820] have demonstrated reductions in relapse rates. In RCT discontinuation studies, mirtazapine [748] and clomipramine [821] have demonstrated continued improvement compared with placebo over approximately six to 12 months. Additional data support the long-term efficacy of fluoxetine, fluvoxamine XR, and sertraline over six to 24 months [710,822-824].

### Biological and alternative therapies

*Biological therapies:* Biological therapies may be useful in patients with OCD who have not responded to CBT and multiple medication trials. Open trials have suggested that rTMS may be a promising adjunctive therapy in patients with treatment-refractory OCD [825,826]. However, results of sham-controlled trials are conflicting, with some trials finding significant improvements [827,828] and others concluding that rTMS was ineffective for treatment-resistant OCD (Level 1, conflicting) [829-831]. Some data suggest that rTMS may improve comorbid depressive symptoms in patients with OCD [829,830].

Several very small studies have suggested that deep brain stimulation may improve symptoms and functionality in up to two-thirds of patients with highly treatment-refractory OCD (Level 4) [832-834].

Open trials suggest that capsulotomy (Level 3) [835-839] or cingulotomy (Level 3) [840-842] may be effective in reducing symptoms in patients with severe, treatment-refractory OCD, however these treatments are usually considered last resorts.

*Alternative therapies:* A meta-analysis of meditation therapies found only two small studies and showed that transcendental meditation and Kundalini yoga were likely no more effective than other kinds of relaxation therapies in treating OCD (Level 3, negative) [843]. Open studies suggest that adjunctive moderate-intensity aerobic exercise may help improve OCD symptoms (Level 3) [844,845].

Small RCTs and open trials have suggested that herbal therapies such as milk thistle (Silybum marianum L. Gaertn.) (Level 2) [715], valerian root (Valeriana officinalis L.) (Level 2) [846], and St John’s wort (Hypericum perforatum) (Level 3) [847] may be useful in patients with OCD. Unfortunately, because these preparations are poorly standardized and have substantial variation in the proportion of the active ingredient in different products, they cannot be widely recommended. These therapies may be useful for some patients; however, more data are needed.

### Summary

OCD is a relatively rare, yet severe, mental disorder, with an onset in the 20s or earlier. It is characterized by the presence of obsessions (persistent, intrusive thoughts) and/or compulsions (repetitive behaviors the individual feels compelled to perform). OCD is associated with substantial functional impairment and a high prevalence of comorbid disorders.

CBT, and notably ERP, are effective first-line options for the treatment of OCD, being equivalent or superior to pharmacotherapy. CBT can be effectively delivered in both individual and group settings, as well as via self-exposure, self-help books, telephone, and internet-based programs. The benefits of CBT are maintained over one to five years of follow-up. The combination of psychotherapy and pharmacotherapy appears to be superior to pharmacotherapy alone, but not to CBT alone, and data suggest that adding CBT to pharmacological treatment may yield better long-term outcomes.

Pharmacotherapeutic approaches should begin with a first-line SSRI such as escitalopram, fluoxetine, fluvoxamine, paroxetine, or sertraline. If response to optimal doses is inadequate or the agent is not tolerated, therapy should be switched to another first-line agent before considering second-line medications. Second-line choices include citalopram, clomipramine, mirtazapine, and venlafaxine XR. OCD can be difficult to treat; therefore, in order to preserve any benefits of a therapy, adjunctive strategies may be important early in treatment.

Patients who do not respond to multiple courses of therapy are considered to have treatment-refractory illness. In such patients it is important to reassess the diagnosis and consider comorbid medical and psychiatric conditions that may be affecting response to therapy. Third-line agents, adjunctive therapies, as well as biological and alternative therapies may be useful when patients fail to respond to optimal treatment trials of first- and second-line therapies used alone and in combination.

## Posttraumatic stress disorder

### Epidemiology

The lifetime prevalence of PTSD in Canada was estimated to be 9.2%, and current (1-month) rates were 2.4% [848]. Over 76% of Canadians reported exposure to a significantly traumatic event [848]. US and European community studies report lifetime prevalence rates of 6.4-6.8% and 12-month rates of 1.1-3.5% [2,849,850]. The most common forms of trauma resulting in PTSD included unexpected death of someone close, sexual assault, serious illness or injury to someone close, having a child with serious illness, and being beaten by a partner or caregiver [848-850]. Onset is generally in the mid to late 20s [2], and the prevalence is about twice as high among women versus men [849,851].

PTSD was associated with significant QoL [852] and functional impairments [848,853-855], which increase with increasing severity of symptoms [855]. In addition, PTSD is associated with high rates of chronic pain [856-859], sleep problems [860], and sexual dysfunction [861], as well as cognitive dysfunction [862,863] and alexithymia [864]. The risk of suicide attempts is increased two- to three-fold by the presence of PTSD [20,849,865].

In primary care, PTSD was associated with more and longer hospitalizations as well as a greater use of mental health care [866]. Among Canadian military personnel, greater use of mental health care was associated with cumulative lifetime trauma exposure, index trauma type, PTSD symptom interference, suicidal ideation, female gender, and comorbid MDD [867,868].

#### Comorbidity

An estimated 75% of patients with PTSD have another comorbid psychiatric disorder [3,848]; and rates are particularly high for other anxiety and related disorders [3,849,859,869,870], MDD [3,849,859,871,872], oppositional defiant disorder [3], ADHD [3], SUD [849], alcohol dependence [3,873], and borderline personality disorder (BPD) [874]. Comorbid panic or mood disorders have been associated with greater functional impairment than PTSD alone [870,871]. Patients with comorbid PTSD and BPD had a poorer QoL, more comorbidity with other psychiatric conditions, and increased odds of a lifetime suicide attempt versus patients with either condition alone [20,874].

### Diagnosis

By definition PTSD requires exposure to trauma, including actual or threatened death, serious injury, or sexual violation [26]. It is characterized by intrusive and distressing memories or dreams, dissociative reactions, and substantial psychological or physiological distress related to the event (Table 28) [26]. A diagnosis of PTSD requires the disturbances to be present for longer than one month; symptoms of >3 days but less than one month may be diagnosed as acute stress disorder (ASD), if the required ASD criteria are met [26].

**Table 28:** DSM-5 diagnosis of PTSD

• The person has been exposed to actual or threatened death, serious injury, or sexual violation in ≥1 of the following ways: ○ Directly experienced or witnessed the traumatic event, learned that trauma occurred to close family member or friend (actual or threatened death must have been violent or accidental), experienced repeated exposure to aversive details of trauma
• Presence of ≥1 of the following intrusion symptoms associated with the trauma: ○ Recurrent, involuntary, and intrusive distressing memories, distressing dreams, dissociative reactions (e.g., flashbacks), psychological or physiological distress at reminders of trauma
• Persistent avoidance of stimuli associated with the trauma, including ≥1 of the following: ○ Avoidance of distressing memories or feelings and external reminders (e.g., people, places) of the trauma
• Negative alterations in cognitions and mood associated with the trauma, including ≥2 of the following: ○ Inability to recall important aspect of the trauma, diminished interest or participation in activities, feeling of detachment or estrangement from others, persistent negative beliefs, distorted blame, and negative emotional state
• Marked alterations in arousal and reactivity associated with the trauma, including ≥2 of the following: ○ Irritable or aggressive behavior, reckless or self-destructive behavior, hypervigilance, exaggerated startle response, problems with concentration, sleep disturbance
• Duration of disturbance >1 month
• Symptoms cause clinically significant distress or impaired functioning
• Specify whether with dissociative symptoms (depersonalization or derealization) or with delayed expression (full criteria not met until at least 6 months after the event)

Adapted from DSM-5 [26].

Compared to the DSM-IV-TR [144], changes to the diagnostic criteria for PTSD in the DSM-5 include adjusting the symptom clusters, adding some new symptoms, and re-classifying PTSD as a ‘‘trauma- and stressor-related disorder’’ instead of an anxiety disorder [26,875]. In addition to PTSD, this new category also includes diagnostic criteria for reactive attachment disorder, disinhibited social engagement disorder, ASD, and adjustment disorders [26]. The DSM-5 diagnostic criteria for PTSD sharpens the definition of “traumatic event,” and there are now four symptom clusters rather than three with the “avoidance” and “numbing of responsiveness” being separated (Table 28). The DSM-5 also eliminated the acute and chronic PTSD specifiers. The PTSD diagnostic criteria apply to adults, adolescents, and children >6 years of age. A subtype has been added for children ≤6 years of age, as well as a dissociative symptoms specifier for patients of all ages [26].

While the most up-to-date DSM-5 diagnostic criteria are being presented here, it is important to note that the treatment data described within this section are based on patients meeting DSM-IV criteria (or older).

PTSD is frequently comorbid with other psychiatric disorders, including other anxiety and related disorders, MDD, and SUDs, which may complicate diagnosis and management [849,859]. In addition, patients with PTSD frequently present with somatic symptoms and pain [859]. It is important to ask patients with psychological or somatic symptoms about trauma [32,859].

### Prevention and early intervention

A number of studies have assessed early intervention with psychological and pharmacological strategies for the prevention of PTSD. Meta-analyses do not support the efficacy of wide spread use of single-session [876,877] or multiple-session [878] psychological debriefing after trauma in preventing or reducing the intensity of PTSD in individuals who have been exposed to a traumatic event but have not been identified as suffering from any specific psychological difficulties. In fact, these interventions may have an adverse effect on some individuals [876,878]. These findings pertain to individual debriefings only; there is insufficient evidence to comment on the utility of group debriefings.

Conversely, meta-analyses have demonstrated the benefit of multisession trauma-focused-CBT (TF-CBT) in patients with ASD or PTSD [879,880]. Therefore, debriefing of all trauma victims is not recommended, rather, screening and treating appropriate individuals is preferred [876]. For the prevention of chronic PTSD in patients with ASD or acute PTSD, brief TF-CBT was more effective than both wait-list and supportive counseling interventions, but there was no evidence of the effectiveness of structured writing compared to minimal intervention [880].

There are few data on the use of pharmacotherapy for the prevention of PTSD. In a cohort study and a RCT, the early use of benzodiazepines following trauma was not beneficial, and may increase the risk of developing PTSD [881,882]. Similarly, retrospective data suggested that gabapentin or pregabalin had no effect on PTSD development [883]. Data from cohort studies on the use of the beta-blocker propranolol have been conflicting [884-888], but one small RCT did show a significant decrease in the severity of PTSD symptoms and lower likelihood of developing subsequent PTSD [889]. SSRI therapy was significantly more effective than placebo in preventing PTSD symptoms according to parent reports but not child reports in a RCT in children [890]. Cohort studies suggest that the early use of morphine during trauma care may reduce the risk of the subsequent development of PTSD in children and adults [891-894].

### Psychological treatment

Psychological therapies for PTSD generally include education about the disorder and its treatment, as well as exposure to cues relating to the traumatic event. Psychotherapy has demonstrated significant efficacy, although a meta-analysis suggested it may be less effective than pharmacotherapy in improving PTSD and comorbid depression symptoms [895].

Meta-analyses of over 30 RCTs of psychological interventions provide evidence of the efficacy of several CBT approaches for the management of chronic PTSD compared with wait-list or usual care control groups [66,67]. There was evidence that individual TF-CBT, EMDR, stress management, and group TF-CBT were effective, while other nontrauma focused psychological treatments (supportive therapy, nondirective counseling, psychodynamic therapy, and hypnotherapy) did not reduce PTSD symptoms as significantly [66,67]. Individual TF-CBT and EMDR appeared to be equally effective, but superior to stress management in the treatment of PTSD [66]. Another meta-analysis also found EMDR and TF-CBT were equally effective [68]. However, in a head-to-head RCT, EMDR resulted in faster recovery compared with the more gradual improvement with brief TF-CBT [896]. Cognitive therapy approaches have been used effectively in treating PTSD following sexual or interpersonal violence [897-901], civilian trauma [902-908], and military trauma [909-914].

Cognitive processing therapy (CPT) is an effective protocol that combines cognitive therapy and written accounts [899-901,910-913]; however, an analysis of the components found no differences in outcomes with either component alone or the combined protocol [899].

Prolonged exposure (PE) is a widely studied CBT approach. A meta-analysis of 13 RCTs concluded that PE therapy was more effective than wait-list or psychological placebo control conditions, and as effective as other active treatments (e.g., CBT, CPT, EMDR) [69]. One study found that 30-minute imaginal exposure sessions were as effective as 60-minute sessions [915]. Imaginal appears to be as effective as *in vivo* exposure [69,916].

Data are conflicting as to the benefits of adding cognitive restructuring to exposure therapy; several studies suggest that exposure alone is superior to the combination [917-919], however, another large RCT found the combination to be significantly better than imaginal or *in vivo* exposure alone [916]. When used as an adjunct to exposure therapy, cognitive restructuring may improve non-fear problems like anger and guilt, and may be a useful adjunct in patients in which these emotions predominate [920,921]. Similarly, the addition of social emotional rehabilitation to exposure therapy did not improve PTSD symptoms but did improve social functioning in male combat veterans with chronic PTSD [922].

Meta-analyses and systematic reviews reveal two current limitations of CBT for PTSD. The first is that about one-third to one-half of patients experience substantial residual symptoms and functional impairments posttreatment, still report symptoms meeting diagnostic criteria at follow-up, or relapse and require booster sessions [923-925]. The second issue pertains to external validity. While CBT for PTSD has been shown to be efficacious in RCTs, there is a dearth of effectiveness studies to suggest that CBT can be generalized to many patients commonly found in clinical practice. Many RCTs have excluded patients with complex clinical profiles including childhood abuse histories, current SUDs, personality disorders, suicidality or self-injurious behavior, homelessness, refugees, intimate partner violence, and significant dissociative symptoms among others [926,927]. In this regard, Bradley et al. [923] found a positive association between the number of exclusion criteria and the strength of effect sizes, such that studies with stricter inclusion criteria tended to report larger treatment effects. Additionally, numerous studies fail to report whether patients experience any adverse effects from psychological treatments [66], or whether dropout rates (ranging between 0-50%) result from treatment demands.

Dialectical behavior therapy (DBT), which was developed to reduce self-harm behavior in patients with BPD, was shown to be useful in patients with PTSD [928-930]. When used as a pretreatment, DBT reduced self-harm behaviors allowing over half of patients to become suitable candidates for PTSD treatment [929].

Another study [931] demonstrated some success with PE treatment of PTSD and comorbid substance abuse. Results of a recent expert clinician survey on best practices suggests that CBT is useful for fear-based PTSD, while this treatment approach may require an additional treatment module targeting affective regulation for patients presenting with a diagnosis of Disorders of Extreme Stress (DESNOS) or complex PTSD [932].

Internet-based treatments are being increasingly investigated, in part because they can be administered remotely and anonymously to under-served or disaster-stricken areas at a relatively low-cost [933]. RCTs have shown that therapist-assisted ICBT is more effective than wait-list or supportive care control strategies in improving PTSD symptoms, depression, anxiety, and disability [934-940]. In addition, a strong therapeutic relationship can be established through the internet, which improved the treatment process [936]. VRE therapy has also demonstrated some utility in improving PTSD symptoms [941-943]. Compared to face-to-face CBT, video-conference CBT was equally effective [944] but telehealth CBT was less effective [914]; however, both were effective compared with pre-treatment.

#### Combined psychological and pharmacological treatment

Research evaluating combined treatment in PTSD is limited; a meta-analysis found only four small trials [945]. Combination SSRI plus psychotherapy was not superior to psychotherapy alone in two RCTs [946,947], but was superior to pharmacotherapy alone in the other two trials [948,949]. In contrast, a more recent RCT found that combination therapy was superior to psychotherapy alone [950]. The role of combining psychotherapy and medication requires further study.

Adjunctive propranolol with trauma reactivation therapy was found to help prevent reconsolidation of the traumatic memory and thus decreased physiological responses and PTSD symptoms during subsequent follow-up in randomized and open trials [951,952]. Two RCTs have found that use of d-cycloserine did not enhance the overall treatment effects of exposure therapy [953,954], and may in fact decrease response to psychotherapy [954].

#### Long-term effects of psychological treatment

Open follow-up data of psychological treatments suggest that benefits are maintained at six- to 18-month assessments after treatment [923,955-958]. Longer-term follow-up of patients treated with EMDR showed that benefits were maintained at three years, with the majority of patients who had initially remitted being at full working capacity [959]. Very long-term follow-up showed that improvements in PTSD and related symptoms achieved with CPT and PE were maintained over an extended five to 10 year period [901].

### Pharmacological treatment

The management of patients with PTSD should follow the principles discussed in Section 2. Pharmacological interventions that have good evidence for efficacy in treating PTSD include fluoxetine, paroxetine, sertraline, and venlafaxine XR. Treatments that have been investigated for use in PTSD have been assessed according to the criteria for strength of evidence (Tables 1 and 2) and are summarized in Tables 29 and 30.

**Table 29:** Strength of evidence of pharmacotherapy for core symptoms of PTSD

Agent	Level of evidence	Agent	Level of evidence
***Antidepressants***			

**SSRIs**		**TCAs**	
Fluoxetine [960-965]	1*	Imipramine [992,993]	1
Paroxetine [966-970]	1	Amitriptyline [994]	2
Sertraline [971-978]	1*	Desipramine [970,995]	2*
		
Fluvoxamine [979-984]	2	**MAOIs and RIMAs**	
Escitalopram [985]	3	Phenelzine [992,993,996]	1*
Citalopram [974,986,787,988]	2 (-ve)	Moclobemide [997,998]	3
		
**SNRIs**		**Other antidepressants**	
Venlafaxine XR [975,989]	1	Mirtazapine [999-1002]	2
Duloxetine [990,991]	3	Reboxetine [984]	2
		Bupropion SR [1003]	3
		Tianeptine [997,1004]	3
		Adjunctive bupropion SR [1005]	2 (-ve)

***Other therapies***			

**Anxiolytics**		**Anticonvulsants**	
** Benzodiazepines**		Topiramate [1009,1010]	1*
Alprazolam [1006]	2 (-ve)	Lamotrigine [1011]	2
Clonazepam [881,1007,1008]	3 (-ve)	Carbamazepine [1012,1013]	3
		
**Atypical antipsychotics**		Divalproex [1014-1017]	1 (-ve)
Risperidone [1030]	2	Tiagabine [1018]	2 (-ve)
Aripiprazole [1031-1033]	3	Adjunctive gabapentin [1019,1020]	4
Quetiapine [1034,1035]	3	Adjunctive levetiracetam [1021]	4
Olanzapine [1036-1038]	2 (-ve)	Adjunctive pregabalin [1022]	4
Adjunctive risperidone [1039-1044]	1*	Adjunctive tiagabine [1023-1025]	4
Adjunctive olanzapine [1045]	2	Adjunctive topiramate [1026-1029]	2 (-ve)
		
Adjunctive aripiprazole [1033,1046,1047]	3	**Other treatments**	
Adjunctive quetiapine [1048-1050]	3	Buspirone [1051,1052]	4
		Trazodone [1053]	4
		Memantine [1054]	4
		Adjunctive eszopiclone [1055]	2
		Adjunctive clonidine [1056]	3
		Adjunctive guanfacine [1057,1058]	1 (-ve)
		Adjunctive zolpidem [1059]	2 (-ve)

*Conflicting data. MAOI = monoamine oxidase inhibitor; RIMA = reversible inhibitor of monoamine oxidase A; SNRI = serotonin–norepinephrine reuptake inhibitor; SR = sustained release; SSRI = selective serotonin reuptake inhibitor; TCA = tricyclic antidepressant; XR=extended release; (-ve) = negative.

**Table 30:** Recommendations for pharmacotherapy for core symptoms of PTSD

First-line	Fluoxetine, paroxetine, sertraline, venlafaxine XR
**Second-line**	Fluvoxamine, mirtazapine, phenelzine

**Third-line**	Amitriptyline, aripiprazole, bupropion SR, buspirone, carbamazepine, desipramine, duloxetine, escitalopram, imipramine, lamotrigine, memantine, moclobemide, quetiapine, reboxetine, risperidone, tianeptine, topiramate, trazodone

**Adjunctive therapy**	**Second-line:** eszopiclone, olanzapine, risperidone **Third-line**: aripiprazole, clonidine, gabapentin, levetiracetam, pregabalin, quetiapine, reboxetine, tiagabine **Not recommended**: bupropion SR, guanfacine, topiramate, zolpidem

**Not recommended**	Alprazolam, citalopram, clonazepam, desipramine, divalproex, olanzapine, tiagabine

SR = sustained release; XR = extended release.

#### First-line agents

*Antidepressants* (*SSRIs & SNRIs*)*:* Evidence from meta-analyses [895,1060] and RCTs supports the use of the SSRI paroxetine [966-970] and the SNRI venlafaxine XR [975,989] (both Level 1) for the first-line treatment of PTSD. Data with fluoxetine are mixed, with both positive [960-962] and negative [963-965] RCTs (Level 1, conflicting). Similarly, RCTs with sertraline have yielded both positive [971,972,975,976,978] and negative [973,974,977] results (Level 1, conflicting). However, there appear to be sufficient data from the larger RCTs to suggest that these agents can be effective first-line options. Conflicting results may be related to the types of traumas, symptom clusters, and comorbidities included in the various studies.

#### Second-line agents

*Antidepressants:* The efficacy of mirtazapine was demonstrated in one small RCT (Level 2) [1000] and three open trials [999,1001,1002]. In a randomized, open-label trial, response rates were significantly higher with mirtazapine than sertraline [1001].

Fluvoxamine demonstrated efficacy for PTSD in open trials [979-983], and in a RCT was as effective as reboxetine (Level 2) [984].

Phenelzine was more effective than placebo in two RCTs [992,993], but not significantly different from placebo in a RCT crossover study (Level 1, conflicting) [996]. Caution is needed when using MAOIs because of the dietary restrictions and potential for drug interactions.

#### Third-line agents

The following agents are recommended as third-line options because of limited data, side effects, or lack of clinical experience as a primary therapy for the treatment of PTSD.

*Antidepressants:* In small RCTs, imipramine (Level 1) [992,993] and amitriptyline (Level 2) [994] demonstrated some efficacy in patients with PTSD. Data with desipramine are mixed, with one RCT showing significant benefit, which were comparable to paroxetine [970], and the other showing improvements in depression only [995]. While RCTs with the TCAs suggest some benefit with these agents, it appears to be limited.

Reboxetine and fluvoxamine were equally effective in a small RCT (both Level 2) [984], and open-label studies suggest that bupropion SR [1003], duloxetine [990,991], escitalopram [985], moclobemide [997,998], and tianeptine [997,1004] (all Level 3) may be useful in PTSD.

*Anticonvulsants:* Data on topiramate are mixed, with one RCT finding significant benefits over placebo [1010], while the other did not [1009] (Level 1, conflicting). There are also limited data suggesting efficacy of other anticonvulsants, including lamotrigine (Level 2) [1011] and carbamazepine (Level 3) [1012,1013].

*Atypical antipsychotics:* Some data suggest that the atypical antipsychotics, risperidone (Level 2) [1030], aripiprazole (Level 3) [1031,1032], and quetiapine (Level 3) [1034,1035] may be a useful alternative to SSRIs for some patients with PTSD. A meta-analysis of seven RCTs using atypical antipsychotics, either as monotherapy or adjunctively, concluded that these agents may be beneficial in the treatment of PTSD, particularly for the symptom of “intrusion” [1061].

*Other therapies:* Small, open case series have suggested benefits with trazodone [1053], buspirone [1051,1052], and memantine [1054] (all Level 4).

#### Adjunctive therapies

Adjunctive strategies have generally been studied in patients who have had an inadequate response to adequate antidepressant therapy, and can be considered for patients with treatment-resistant PTSD.

*Second-line adjunctive therapies:* In a RCT, adjunctive eszopiclone was significantly more effective than placebo in improving PTSD and sleep symptoms (Level 2) [1055]. There is RCT evidence for the use of adjunctive atypical antipsychotics, including risperidone (Level 1, conflicting) [1039-1044] and olanzapine (Level 2) [1045], for patients with treatment-resistant PTSD. While a number of small RCTs demonstrated benefits with adjunctive risperidone [1039-1042], a large, six-month trial in approximately 250 patients failed to show improvements in PTSD symptoms compared with placebo [1043].

*Third-line adjunctive therapies:* Open-label trials and case series suggest that adjunctive quetiapine [1048-1050] or aripiprazole [1033,1046,1047] (both Level 3) are useful in patients with refractory PTSD.

Similarly, there are some data suggesting adjunctive anticonvulsants including: gabapentin [1019,1020], levetiracetam [1021], pregabalin [1022], or tiagabine [1023-1025] (all Level 4), as well as the alpha-adrenergic agonist clonidine (Level 3) [1056], can improve symptoms in patients with treatment-resistant PTSD.

*Not recommended adjunctive therapies:* Small RCTs failed to show the superiority of adjunctive therapy with guanfacine (Level 1, negative) [1057,1058], bupropion SR [1005] (Level 2, negative), or zolpidem [1059] (Level 2, negative). While case series suggested that adjunctive topiramate [1026,1027,1029] may be effective in treatment-resistant PTSD, a RCT failed to show superiority over placebo [1028] (Level 2, negative).

#### Treatments for specific PTSD-associated symptoms

Several agents have been used to target particular symptoms associated with PTSD. Prazosin has demonstrated significant efficacy for reducing trauma nightmares and improving sleep quality in patients with PTSD compared with placebo (Level 1) [1035,1062-1066]. Some open-label data suggest that naltrexone may help reduce flashbacks (Level 3) [1067-1070], and fluphenazine may improve trauma re-experiencing symptoms (Level 3) [1037].

Cyproheptadine was not effective for nightmares or sleep problems in patients with PTSD and may actually exacerbate sleep disturbance (Level 2, negative) [1071].

#### Not recommended

In general, data do not currently support the use of divalproex [1014-1017] (Level 1, negative), alprazolam [1006], citalopram [974,986-988], olanzapine [1036-1038], tiagabine [1018] (all Level 2, negative), or clonazepam (Level 3, negative) [881,1007,1008].

#### Maintenance pharmacological treatment

Long-term therapy has been evaluated in relapse-prevention and naturalistic follow-up studies. Relapse-prevention studies are those in which responders to SSRI therapy are randomized to continued active treatment or placebo. A meta-analysis of three relapse-prevention studies included 272 patients with PTSD, and found a highly significant reduction in relapse rates with continued SSRI treatment compared with placebo over approximately six months (odds ratio for relapse was 0.25) [497].

In RCT discontinuation studies, fluoxetine [1072,1073] and sertraline [1074] have demonstrated significantly lower relapse rates over six months in the range of 5-22% with active treatment compared to 16-50% with placebo [1072-1074]. However, in a small discontinuation RCT, tiagabine was not superior to placebo in preventing relapse [1075].

Open follow-up studies with paroxetine [1076] and sertraline [1077] have demonstrated sustained and continued improvement over six to 12 months of continued SSRI therapy.

### Biological and alternative therapies

In general, these therapies may be useful for some patients; however, more data are needed.

*Biological therapies:* In RCTs, rTMS was effective as monotherapy or as an adjunct to SSRIs in patients with PTSD (Level 1) [1078-1080], and at least some improvements were maintained at two to three months after treatment [1078,1079]. Open prospective and retrospective data suggest that adjunctive electroconvulsive therapy may be helpful in patients with refractory PTSD (Level 3) [1081,1082].

*Alternative therapies:* In a RCT, acupuncture was more effective than a wait-list control and as effective as group CBT (Level 2) [1083]. Adjunctive use of symptom-oriented hypnotherapy [1059] or mantra repetition [1084] (both Level 2) improved PTSD symptoms in small trials; and in a small case series, patients with PTSD benefited from transcendental meditation (Level 4) [1085].

### Summary

The lifetime prevalence of PTSD is around 6-9%; it is more frequent in women than in men, with an onset generally in the mid to late 20s. PTSD is associated with high rates of functional impairment, somatic complaints, suicide risk, and comorbid psychiatric disorders. A diagnosis of PTSD requires evidence of exposure to trauma, and is characterized by intrusive and dissociative symptoms.

Evidence does not support the wide spread use of early intervention with psychological strategies for the prevention of PTSD. Debriefing of all trauma victims is not recommended, rather, screening and treating appropriate individuals is preferred. In general, there is little evidence supporting the use of pharmacotherapy for the prevention of PTSD, with most studies suggesting no preventive benefits.

CBT is an effective first-line option for the treatment of PTSD. Effective approaches include TF-CBT, EMDR, PE, and stress management therapy. ICBT and VRE have also demonstrated efficacy. Benefits are maintained during long-term follow-up of up to one to 10 years after treatment. Research evaluating combined psychological and pharmacological treatments in PTSD is limited, and this requires further study.

Pharmacotherapeutic approaches should begin with one of the first-line options which include SSRIs such as fluoxetine, paroxetine, or sertraline, or the SNRI venlafaxine XR. If response to optimal doses is inadequate or the agent is not tolerated, therapy should be switched to another first- or second-line agent, or a second-line agent should be added. Patients with PTSD may make few gains during treatment, and it is important to preserve even small gains achieved with initial therapy. Therefore, augmentation with second- or third-line agents may be important early in treatment.

Patients who do not respond to multiple courses of therapy are considered to have treatment-refractory illness. In such patients it is important to reassess the diagnosis and consider comorbid medical and psychiatric conditions that may be affecting response to therapy. Third-line agents, adjunctive therapies, as well as biological and alternative therapies may be useful when patients fail to respond to an optimal treatment trial of first- and second-line therapies used alone and in combination.

## Special populations

### Women during pregnancy and the postpartum period

#### Epidemiology

Women have been found to be at higher risk for anxiety and related disorders than men [2]. Anxiety disorders during the perinatal period are increasingly gaining research attention. Although further investigation is needed, data from a large national survey suggest the overall prevalence of anxiety and related disorders is unchanged in women during pregnancy [1086]; however, other data have found an increased risk for individual disorders, such as GAD [1087,1088]. Similarly, some data suggest that anxiety disorders are also not more prevalent during the postpartum period [1086], but other studies suggest higher rates of OCD and GAD during this period [1089,1090]. In addition, PTSD can develop as a result of pregnancy complications that are experienced as traumatic [1091,1092].

Anxiety and related disorders during pregnancy or postpartum may have a negative impact on the pregnancy, the child, or the mother. While studies report that maternal anxiety disorders are associated with adverse pregnancy outcomes such as a shorter gestational age, premature delivery, or elective cesarean delivery [1093-1095], a meta-analysis found no relationship between anxiety symptoms per se and adverse perinatal outcomes [1096]. Anxiety symptoms during pregnancy have been associated with depressive symptoms, substance use, and anemia, as well as decreased use of prenatal vitamins [1093,1097-1099].

Parenting may also be affected by maternal anxiety and related disorders. Mothers with anxiety disorders have been found to be less promoting of psychological autonomy than those mothers without anxiety [1100]. Maternal anxiety has been found to be predictive of child cognitive development [1101], associated with behavioral/emotional problems in childhood [1101,1102], and maternal anxiety and related disorders have been found to be related to subsequent development of an anxiety disorder in the child [1103].

#### Treatment issues

Psychosocial treatments, with CBT specifically, have strong empirical support for the treatment of anxiety and related disorders [63,70,71,1104], but evidence of their efficacy in perinatal women with anxiety disorders is lacking. Cohort studies have shown beneficial effects of group CBT in pregnant women with B-I-I phobia [1105], and individual CBT in women with OCD in the postnatal period [1106]. Arch et al. argued that although exposure-based CBT or behavioral therapy may have been avoided in the past because of concerns of potential harm, they likely can be viable, safe alternatives in pregnancy [1107]. The lack of data on the use of structured psychosocial interventions for anxiety and related disorders during the perinatal period is a significant gap in the literature.

It is important to consider the risks and benefits of pharmacotherapy during pregnancy and while breastfeeding during the postpartum period. Risks to the fetus and newborn should be weighed against that of the potential harm of untreated anxiety and related disorders, an area that is gaining increasingly more research attention. Treatment decisions should attempt to optimize outcomes for both mother and baby.

Detailed recommendations on the use of psychiatric medications during pregnancy and lactation are available from the American Congress of Obstetricians and Gynecologists (ACOG) Practice Bulletin [1108]. Although it is over five years old, risks associated with various psychotropic medications are summarized [1108]. The FDA pregnancy risk category system has been criticized as being insufficient [1109] and is currently under the process of revision. The Canadian Hospital for Sick Children Motherisk website (http://www.Motherisk.org) is also a useful resource.

*Antidepressants:* There appears to be little evidence of an association between maternal antidepressant use and increased risks of congenital malformations in general, and major congenital malformations in infants [1110-1113]. The exception is a statistically increased risk of cardiac defects with antidepressants, and with paroxetine specifically, although the clinical significance of this has been questioned [1108,1113-1117]. There have been reports of increased rates of spontaneous abortion following antidepressant use during pregnancy; in the most recent meta-analysis, this was not supported using data from studies with higher study quality but found by others who included all studies [1118-1120]. In terms of delivery outcomes, a recent meta-analysis found a statistically increased risk for preterm birth, lower gestational age, birth weight, and APGAR scores – but the effects were small, generally in the normal range, and of questionable clinical significance [1118]. However, data support an increased risk for poor neonatal adaptation syndrome (PNAS) [1121-1123], while findings of increased risk for persistent pulmonary hypertension in the antenatally exposed infant have not been consistent [1124-1127]. Systematic reviews suggest that overall prenatal exposure to antidepressants does not appear to be associated with changes in long-term neurocognitive or behavioral development in children [1128-1130] and that illness itself appears to play a role in negative outcomes (although this study examined the effects of maternal depression) [1131]. Two reports link prenatal antidepressant use to childhood autism spectrum disorders [1132,1133] and two others link bupropion exposure to childhood ADHD [1134,1135]. These studies have limitations and further research is required.

In terms of breastfeeding, potential risks of antidepressant use during lactation must be weighed against the recognized benefits for the infant. Antidepressants are excreted into breast milk and although data are limited, the majority are found in very low amounts with few isolated instances of adverse signs [1108]. If antidepressant treatment is indicated, sertraline or paroxetine is preferred [1136]. Long-term data on potential neurobehavioral effects are lacking. Clinicians can consult LactMed at http://toxnet.nlm.nih.gov/cgi-bin/sis/htmlgen?LACT for the latest information available.

*Benzodiazepines*: The data on benzodiazepines remain more limited. A recent meta-analysis did not find an increased risk of major malformations or cardiac defects following prenatal benzodiazepine exposure, but concluded the significant increase in risk of oral cleft remains based on data derived from case-control studies [1137], although another meta-analysis reported the absolute risk is small (<1%) [1138]. A case-control study published in 2002 examining exposure to five benzodiazepines (including clonazepam) and not included in the above meta-analyses, with over 60,000 infants, did not find an association with various congenital malformations or oral clefts [1139]. Although there are a lack of meta-analytic data, neonatal withdrawal or toxicity syndrome has been described with antenatal benzodiazepine exposure and close monitoring of the infant has been recommended [1108]. The neurobehavioral effects on the child over the long-term due to antenatal exposure have been topics of debate and remain uncertain [1108]. Benzodiazepines are excreted into breast milk at low levels generally. A recent study with 124 mothers documented low levels of adverse effects (sedation in particular) and supported the initiation of breastfeeding [1140]. Caution may be advised regardless however in infants who poorly metabolize benzodiazepines [1108].

*Atypical antipsychotics:* Data on the use of antipsychotics during pregnancy continue to be limited [1141]. Thus far, there does not appear to be an increased risk for malformations although inconsistent data have been reported with some suggesting the data are inconclusive [1141-1143]. These drugs have been found to be associated with both increased and decreased birth weight as well as increased risk for preterm birth [1144-1149]. The second-generation antipsychotics can increase the risk of complications given the risk of metabolic syndrome, and thus diabetes, in the mother. Monitoring has been recommended [1150]. Both the FDA and Health Canada have issued safety alerts advising of the potential risk for abnormal muscle movements and withdrawal symptoms in infants exposed to antipsychotic medications during the 3^rd^ trimester of pregnancy [1151-1153]. Data on breastfeeding are more limited, but levels in breast milk have typically been shown to be low although adverse effects have been reported [1154].

#### Summary

The management of anxiety and related disorders in women who are pregnant or lactating requires careful consideration of both the potential risks of any treatment option as well as risks of an untreated anxiety disorder. Antidepressants are generally associated with low teratogenic risk and adverse delivery outcomes. Patients should be counseled about PNAS and its management. Less is known about the risk of benzodiazepine and atypical antipsychotic exposure during pregnancy as the data are more limited. Treatment must be individualized and decisions should be made with the most up-to-date information with the best course of action decided upon with the patient. Poorly or untreated psychiatric illness carries its own risks, both in the short- and long-term.

### Children and adolescents

#### Epidemiology

Anxiety and related disorders were the most common psychiatric disorders noted in the National Comorbidity Survey-Adolescent supplement (NCS-A) (age 13-18 years), with a lifetime and 12-month prevalence of 31.9% and 24.9%, respectively [1155,1156]. Prevalence rates for individual anxiety and related disorders are shown in Table 31[1155,1156].

**Table 31:** Prevalence estimates of anxiety and related disorders among youths in the NCS-A (age 13-18 years)

Anxiety and related disorder	Estimated prevalence (%)	
	
	12-month	Lifetime
Any anxiety disorder	24.9	31.9
Separation anxiety disorder	1.6	7.6
Specific phobia	15.8	19.3
Social anxiety disorder	8.2	9.1
Posttraumatic stress disorder	3.9	5.0
Panic disorder	1.9	2.3
Generalized anxiety disorder	1.1	2.2

Adapted from references [1155,1156]. NCS-A = National Comorbidity Survey-Adolescent supplement

Specific phobias are very common in children. However, although most adolescents reported at least one fear (77%), lifetime prevalence rates are in the range of 10-35% [308,1156]. A study including children as young as five years of age found lower rates of diagnosed specific phobias (1%) [1157]. B-I-I and animal fears are the most common types reported in pediatric populations [308,1157]. The prevalence of OCD is only 0.25% in children [1158], but is 1-2% in adolescents, which is comparable to the rate seen in adults [2,1159,1160].

In the adolescent population, anxiety and related disorders were found to have the earliest median age of onset (six years), compared to other psychiatric disorders (11-15 years) [1156]. Similarly, in the adult population, the median age of onset was earliest for anxiety and related disorders (11 years) compared to other psychiatric disorders (20-30 years) [2]. Separation anxiety disorder and the phobias (seven to 14 years) have much earlier median ages of onset compared to OCD, GAD, panic disorder, or PTSD (20-30 years) [1,2,1161].

Anxiety and related disorders can have a substantial long-term impact, putting children at elevated risk for MDD, other anxiety disorders, and SUD in adulthood [11,12]. Anxiety and related disorders among younger patients are associated with high rates of comorbid psychiatric conditions [1162-1165], SUD [1166-1169], sleep problems [1170-1173], somatic symptoms [1174], and suicidality [1175], as well as problems with cognition/attention [1164,1176,1177], academic performance [1178,1179], and peer relationships [1180].

#### Diagnostic issues

Diagnostic evaluation of pediatric patients should be based on DSM-5 criteria, but use developmentally appropriate language, and consider collateral information from parents and teachers. Children may express anxiety through crying, tantrums, freezing, or clinging, as well as through play. The DSM-5 provides some modifications to adult criteria to assist in the diagnosis of anxiety and related disorders in children (Table 32) [26]. In particular, a separate subtype for patients ≤6 years of age has been added to the criteria for PTSD to make it more developmentally sensitive to young children [26].

**Table 32:** DSM-5 diagnostic criteria for anxiety and related disorders specific to children

Anxiety or related disorder	DSM-5 diagnoses specific to children
Separation anxiety disorder	• Developmentally inappropriate and excessive fear or anxiety concerning separation from those to whom the individual is attached, as evidenced by ≥3 of the following: ○ Distress when separation occurs, worry about loss or separation, reluctance to leave home, be alone, or go to sleep because of fear of separation, nightmares involving separation, or complaints of physical symptoms (e.g., headaches, upset stomach) when separation occurs • Duration of at least 4 weeks • Onset before 18 years of age • The disturbance causes clinically significant distress or impairment in social, academic (occupational), or other important areas of functioning

Selective mutism	• Consistent failure to speak in specific social situations in which there is an expectation for speaking (e.g., at school) despite speaking in other situations

**Anxiety or related disorder**	**Changes to adult DSM-5 diagnostic criteria specific to children**

Specific phobia	• The fear or anxiety may be expressed by crying, tantrums, freezing, or clinging • Other specifiers: loud sounds or costumed characters

SAD (social phobia)	• The anxiety must occur in peer settings, not just during interactions with adults • The fear or anxiety may be expressed by crying, tantrums, freezing, clinging, shrinking, or failure to speak in social situations

OCD, panic disorder	• No pediatric specific criteria

PTSD	• Qualifiers in children ○ Intrusion symptoms: repetitive play may occur in which themes or aspects of the traumatic event(s) are expressed; there may be frightening dreams without recognizable content; trauma-specific re-enactment may occur in play • Specific subtype for children ≤6 years of age

GAD	• Less stringent criteria for symptoms than in adults

Adapted from DSM-5 [26].

#### Prevention strategies

Psychoeducational programs for children and adolescents aimed at preventing the development of an anxiety or related disorder have shown small, but significant effects [1181]. Both universal (administered to all children within target population) [1182] and indicated prevention programs (administered to children demonstrating highly anxious symptoms) [1183,1184] demonstrate benefits, but indicated programs are associated with larger effect sizes than universal programs [1181].

Both psychological and pharmacological strategies have been assessed for the prevention of PTSD. An early psychological intervention with children involved in road traffic accidents failed to result in any significant benefits over a control group [1185].

In a RCT in burn victims, sertraline was more effective in preventing PTSD symptoms than placebo according to parent report but not child report [890]. Data do not support the use of propranolol in preventing PTSD [1186] or ASD [886] in pediatric injury patients.

#### Treatment issues

##### Psychological treatment

Psychological therapies for children often need to be adapted to suit the chronological and developmental ages of young patients and to include parental involvement. Meta-analyses support the efficacy of CBT for the treatment of anxiety and related disorders in children and adolescents [1187-1191]. A meta-analysis of 24 clinical trials showed that almost 70% of youths who received CBT no longer met diagnostic criteria for their anxiety disorder compared to only 13% of wait-list controls [1189]. Meta-analyses and RCTs have confirmed the efficacy of CBT in children with SAD [1192-1197], panic disorder [1198], OCD [1199-1204], PTSD [946,1205-1211], school refusal [1212-1215], and separation anxiety disorder [1216].

CBT has demonstrated efficacy in both group and individual formats [1189,1190,1194,1217,1218], as well as in computer- or internet-based formats [1219,1220].

One commonly used pediatric CBT protocol is the “Coping Cat” program [1221,1222], which has demonstrated efficacy in RCTs [1221,1223,1224] and in long-term follow-up studies [1222,1225]. In a RCT, Coping Cat CBT was as effective as pharmacotherapy with an SSRI, but less effective than combination therapy [1223,1224].

Additional specific psychological approaches that have demonstrated efficacy in treating anxiety in children and adolescents include: attention bias modification (ABM) [1226], MBCT [1195], and social effectiveness therapy (SET) [1227,1228] for SAD; ERP [1229,1230], family-based CBT [1231,1232], and meta-cognitive therapy [1229] for OCD; cognitive behavioral writing therapy (CBWT) [1233], spiritual-hypnosis assisted therapy (SHAT) [1234], emotion regulation therapy [1235], exposure therapy [1236], and EMDR [905,1237,1238] for PTSD; and exposure therapy for specific phobias [313].

Approaches that include parental or family involvement may have some additional benefit over strategies that include children only [1239-1245], especially when parents suffer from an anxiety or related disorder themselves [1246]. Parental training only has also demonstrated beneficial effects on children with an anxiety disorder [1247,1248].

The presence of comorbidities may have a negative impact on the efficacy of CBT in pediatric patients [1249]. However, integrated CBT protocols designed to target both conditions have demonstrated efficacy in youths with anxiety and related disorders and comorbid ADHD [1250], aggression [1251], or comorbid SUD [1252].

Long-term follow-up studies have shown sustained benefits of CBT over two to seven years posttreatment [1218,1225,1228,1253,1254].

##### Pharmacological treatment

Complete treatment recommendations for the management of anxiety and related disorders in youths are beyond the scope of these guidelines and the reader is referred to specific guidelines for the assessment and treatment of children and adolescents with anxiety disorders, such as those developed by the American Academy of Child and Adolescent Psychiatry (AACAP) [1255-1258].

For children and adolescents, psychological treatments are generally preferred over pharmacotherapy, or if warranted combination therapy may be an option. RCTs comparing combined pharmacological and psychological treatments in younger patients with anxiety have demonstrated efficacy equal or superior to either treatment alone [1199,1223,1224,1259]. In the pediatric population, safety concerns associated with antidepressants (see “Safety Issues”) should be weighed against the potential benefits of therapy. Medication may be warranted in children and adolescents with severe impairment or those who are unlikely to respond to CBT due to cognitive or other issues. A “start low and go slow” approach is advised when using medications in this patient population.

The strength of evidence for pharmacotherapeutic agents in the treatment of pediatric patients is shown in Table 33. When pharmacotherapy is felt to be warranted, SSRIs are generally preferred for children and adolescents with anxiety and related disorders.

**Table 33:** Strength of evidence of treatments for anxiety and related disorders in children and adolescents

Disorder	Antidepressants	Benzodiazepines and other treatments
OCD	Fluoxetine (Level 1) [1264-1269]	**Antipsychotics**
	Clomipramine (Level 1) [1274-1276]	Adjunctive aripiprazole (Level 3) [1293]
	Citalopram (Level 2) [1264,1270]	**Other**
	Fluvoxamine (Level 2) [1271]	Riluzole (Level 4) [1294]
	Paroxetine (Level 2) [1272]	
	Sertraline (Level 2) [1273]	

Panic disorder		**Anxiolytics**
		Clonazepam (Level 4) [1287,1288]
		Alprazolam (Level 4) [1289]

SAD	Fluoxetine (Level 1) [1227,1277]	**Anxiolytics**
	Fluvoxamine (Level 2) [1278]	Alprazolam (Level 2, -ve) [1290]
	Paroxetine (Level 2) [1279]	
	Venlafaxine XR (Level 2) [1282]	
	Escitalopram (Level 3) [1280]	
	Sertraline (Level 3) [1281]	
	Mirtazapine (Level 3) [1283]	

Separation anxiety disorder	Fluoxetine (Level 2) [1277]	**Anxiolytics**
	Fluvoxamine (Level 2) [1278]	Clonazepam (Level 2, -ve) [1292]

GAD	Fluoxetine (Level 2) [1277]	**Anxiolytics**
	Fluvoxamine (Level 2) [1278]	Alprazolam (Level 2, -ve) [1290]
	Sertraline (Level 2) [1284]	

School-refusal	Citalopram (Level 4) [1285]	**Anxiolytics**
	Adjunctive imipramine (Level 2) [1259]	Alprazolam (Level 2, -ve) [1291]

PTSD	Sertraline (Level 2, -ve) [1286]	
	Adjunctive sertraline (Level 2, -ve) [946]	

XR = extended release; (-ve) = negative.

*Antidepressants:* SSRIs and TCAs have been well studied in pediatric patients with anxiety and related disorders (Table 33) [1260-1262], although these agents should be used with caution in youths as discussed in the section on safety issues below. Most of the data in pediatric patients are in those with OCD [1204,1261,1263] or SAD [1197].

There is good evidence for the efficacy of SSRIs in children and adolescents with OCD, including fluoxetine (Level 1) [1264-1269], citalopram (Level 2) [1264,1270], fluvoxamine (Level 2) [1271], paroxetine (Level 2) [1272], and sertraline (Level 2) [1273], as well as for the TCA clomipramine (Level 1) [1274-1276].

Similarly, there is good evidence for the efficacy of SSRIs in SAD, including fluoxetine (Level 1) [1227,1277], fluvoxamine (Level 2) [1278], paroxetine (Level 2) [1279], escitalopram (Level 3) [1280], and sertraline (Level 3) [1281], as well as for the SNRI venlafaxine XR (Level 2) [1282], and some evidence for mirtazapine (Level 3) [1283].

There is level 2 evidence for the efficacy of fluoxetine [1277] and fluvoxamine [1278] in separation anxiety disorder, and for fluoxetine [1277], fluvoxamine [1278], and sertraline [1284] in GAD. In school-refusing children and adolescents, a small case-series suggested benefit with citalopram (Level 4) [1285], and a RCT demonstrated that imipramine as an adjunct to CBT was more effective than CBT alone (Level 2) [1259].

In pediatric PTSD, sertraline alone [1286] or as an adjunct to CBT [946] was not more effective than placebo or CBT (both Level 2, negative) and cannot be recommended at this time.

*Benzodiazepines:* There are little data demonstrating the efficacy of benzodiazepines in children and adolescents with anxiety and related disorders (Table 33) [1287-1292]. In fact, the few RCTs have demonstrated no significant improvements in anxiety symptoms with alprazolam over placebo in overanxious or avoidant disorders (Level 2, negative) [1290] or school-refusal (Level 2, negative) [1291], or with clonazepam in separation anxiety disorder (Level 2, negative) [1292]. Benzodiazepines have limited utility in youths, although they may be useful for short-term therapy in specific situations where there is a need to achieve rapid reduction in severe anxiety symptoms to allow exposure-related psychotherapy (e.g., panic disorder, school refusal behavior).

*Other treatments:* In open trials in pediatric patients with treatment-resistant OCD, the atypical antipsychotic aripiprazole (Level 3) [1293] and the glutamate antagonist riluzole (Level 4) [1294] have demonstrated some efficacy.

##### Combination psychological and pharmacological therapies

The combination of sertraline and CBT was significantly superior to both monotherapies in a large RCT in pediatric patients with separation anxiety disorder, GAD, or SAD [1223]. In pediatric patients with OCD, the addition of CBT in those with a partial response to SSRIs resulted in significantly greater response rates compared with the SSRI alone [1199], while the addition of d-cycloserine to CBT was not superior to placebo [1295].

##### Alternative therapies

There is currently little evidence supporting the efficacy of exercise in reducing anxiety symptoms in pediatric populations [1296], although some open data suggest it may have a small beneficial effect in pediatric PTSD [1297,1298].

##### Safety issues

An important consideration when using antidepressant medications in children and adolescents is the potential for an increased risk of suicidality. Clinicians should be aware of the potential activating side effects of SSRIs (insomnia, agitation, tremor, and anxiety), especially in young children [1299-1301]. Regulatory bodies in many countries have issued black-box warnings about suicidal ideation/suicide attempts with the use of antidepressants in patients younger than 19 years. However, in a comprehensive analysis, the pooled absolute risk difference for suicidal thinking or behavior between SSRI- and placebo-treated youth with anxiety and related disorders was non-significant (0.5-0.7%), and lower than the risk for youth treated for MDD (0.9%) [1302]. Anxiety and related disorders also increase the risk of suicidality — nearly eight times for suicidal ideation and six times for suicide attempts compared with not having an anxiety disorder [1175]. Therefore, risks and benefits of treatment should be discussed with both children and their parents.

The most common antidepressant adverse events are generally activation and vomiting in children, and somnolence in adolescents [1303]. More conservative dosing strategies may be needed especially in younger children or those with low body weight [1301].

#### Summary

The management of anxiety and related disorders in children and adolescents can be challenging. Diagnostic evaluation of pediatric patients should use developmentally appropriate language and consider collateral information from parents and teachers. Children may express anxiety through crying, tantrums, freezing, or clinging, as well as through play. For children and adolescents, psychological therapies are generally preferred over pharmacotherapy, or if warranted combination therapy may be an option. Psychological therapies often need to be adapted to suit the chronological and developmental ages of young patients and to include parental involvement. When pharmacotherapy is warranted, SSRIs are generally preferred, although antidepressants should be used with caution in pediatric patients.

### Elderly

#### Epidemiology

The lifetime and 12-month prevalence of any anxiety or related disorder among those age 65 or older is estimated to be 13.6% and 7.0%, respectively, compared with 27.8% and 17.8% in the overall adult population [1304]. Including subthreshold anxiety increases the 12-month prevalence from about 6% to over 26% in older adults [1305]. The prevalence rates of anxiety and related disorders have generally been shown to decline with age, and as in younger age groups, the prevalence is higher in women than in men [509,1304,1306,1307]. The decline in prevalence may be related to age biases in the assessment of anxiety and the masking effect of other risk factors that increase with aging [1308]. Under-diagnosis is common, with one study finding that only 34% of older patients with GAD had previously had anxiety symptoms documented [1309].

Among older adults (≥55 years) with mood or anxiety and related disorders, 60-70% do not use mental health care services [1310,1311], although use is higher among those with comorbid disorders [1312]. Older adults with anxiety and related disorders have higher rates of sleep disturbances [1313-1315] and greater impairment in cognitive functioning [1316-1319] compared to those without anxiety disorder. In addition, anxiety negatively impacts physical functioning and mobility [1320,1321], and health related QoL [1321,1322].

##### Comorbidities

Depression is among the most common comorbid disorders among older adults with anxiety and related disorders [1323-1325], and is associated with poorer outcomes of both disorders [1326]. Approximately 80% of adults ≥65 years of age have at least one chronic medical condition, and this may be even higher among those with anxiety disorders [1327]. Older patients with anxiety and related disorders report higher rates of diabetes, gastrointestinal conditions, and dementia [1325,1327,1328].

Chronic urinary incontinence, hearing impairment, hypertension, respiratory disease, and poor sleep were associated with elevated rates of anxiety symptoms or disorders [1315,1329]. Comorbid anxiety in patients with medical illnesses, particularly cardiovascular disease, has been associated with an increased risk of mortality [1330,1331]. Furthermore, the relationship between anxiety and related disorders in the elderly and cognitive impairment remains largely neglected [1332].

#### Diagnostic issues

The recognition and accurate diagnosis of anxiety and related disorders in older patients can be challenging [1333]. Modifications to the DSM-5 diagnostic criteria may assist clinicians in more accurately recognizing and diagnosing anxiety and related disorders in the elderly [1333].

Older patients with anxiety often present differently than younger patients [1327,1334]. Avoidance and excessive anxiety may be difficult to detect in older patients [1333]. Older adults may describe symptoms differently; for example, they may discuss concerns rather than worries [1327,1333]. They are less likely to attribute symptoms to anxiety and related disorders, but rather may attribute them to physical illness and they may have difficulty remembering symptoms [1327,1335]. Obtaining information from collateral sources may be useful. Assessing impact on work or social functioning may also be complicated by changes in responsibilities associated with aging (e.g., retirement) [1333]. It may be helpful to ask about activities relevant to older adults, such as visiting grandchildren. Similarly, avoidance may be harder to detect because of limitations in physical mobility or visual problems, leading to a decline in activities outside the home [1336].

Chronic medical illness or the use of medications can also complicate the diagnosis of anxiety and related disorders [1333]. Determining which came first, the physical illness or the anxiety symptoms can be helpful. However, when a medical illness is chronic, this precludes the likelihood that the anxiety would resolve when the medical condition resolves.

Late-onset anxiety and related disorders are relatively unusual [2], therefore older patients with new onset anxiety should be investigated for potential causative factors (e.g., physical illness, medication side effects).

#### Psychological treatment

Relaxation training, CBT, supportive therapy, and CT have support for the treatment of anxiety symptoms and disorders in older patients [1337]. Meta-analyses suggest the efficacy of psychological treatment is similar to that of pharmacotherapy for the treatment of anxiety and related disorders in older patients [1338,1339].

In meta-analyses, CBT was an effective option in reducing anxiety symptoms among older patients compared to wait-list or active controls [1340-1342]. Some data suggest that CBT may be less effective for anxiety and related disorders in older patients than in working-age adults [1337,1342]. Older patients may benefit from the inclusion of learning- and memory-aids with standard CBT [1343,1344]. In RCTs in older patients, CBT demonstrated efficacy for the treatment of GAD [1337,1343,1345-1347] and panic disorder [1348,1349]. Exposure therapy, with or without CBT, demonstrated efficacy in case-controlled studies in patients with PTSD [1350] or specific phobias [1351]. CBT may also be effectively delivered via telephone, although improvements may not be long lasting [1352].

In a case-control study, regular physical exercise reduced the risk of developing anxiety disorders among older adults [1353].

#### Pharmacological treatment

Data suggest that pharmacotherapy including antidepressants or anticonvulsants is likely as effective in older adults as it is in younger patients [575]. Most of the studies in older patients include those ≥60 or 65 years and have been conducted in patients with GAD or panic disorder.

The most robust data in elderly patients with GAD are from a large RCT (n=273), which demonstrated significant improvements and good tolerability with pregabalin compared with placebo [1354]. Pregabalin was also effective as adjunctive therapy in an open trial in older patients with comorbid GAD and depression [1355].

Pooled analyses of subsets of older patients from multiple RCTs demonstrate that duloxetine [1356] and venlafaxine [575] were effective for the treatment of GAD. Citalopram was effective in an eight-week RCT [1357] and in an open study over six months of treatment [1358]. Some data suggest that escitalopram may be useful in older patients with GAD [550,1359]; although in one RCT, response rates were not significantly different than placebo in the intention-to-treat analysis [550]. Sertraline was more effective than CBT [1349], particularly at a one-year follow-up assessment [1360], and was as effective as buspirone [561] in older adults with GAD.

In older patients with panic disorder, paroxetine was as effective as CBT and more effective than a wait-list control, and results were sustained at a six-month follow-up [1348]. Escitalopram [1361] and citalopram [1361] were equally effective in a small, open trial. A small, open trial also showed fluvoxamine to be effective in older patients with GAD, panic disorder, or OCD [1362]. Data in patients with MDD, suggest that mirtazapine may have beneficial anxiolytic effects in the elderly [1363,1364].

Data show that 45-60% of older patients (>55 years) with anxiety and related disorders are prescribed a benzodiazepine, which is higher than the rate of antidepressant use [1365-1367]. The very high use of these agents is a cause for concern since they are not a preferred long-term treatment strategy and elderly patients may be more sensitive to their negative effects [1365,1366].

##### Safety issues

The elderly maybe more susceptible to adverse drug events and drug-drug interactions (DDIs) due to gradual age-related physiologic changes that affect the pharmacokinetic and pharmacodynamic properties of many medications [1368,1369]. Factors which may alter drug metabolism and plasma concentrations among elderly patients include frailty, reduced homoeostatic mechanisms, and psychosocial issues [1368]. Age-related changes in body composition can result in increases or decreases of drug volume distribution, and hepatic or renal dysfunction can impair drug metabolism and drug clearance [1369,1370]. All of these changes are highly variable in elderly patients, further complicating use of medications in this population [1368,1369]. A review of the literature found that almost half of available antidepressants are associated with age-related clearance changes and identified at least 45 medications that could interact with specific antidepressants [1371].

DDIs may be more common in older adults because of the greater number of concomitant medication they may be taking to treat multiple comorbid conditions. In one study of US community-dwelling older adults, almost 30% used at least five prescription medications, 80% used at least one prescription medication, and almost half used over-the-counter and dietary supplements [1372].

Psychotropic medications have been associated with an increased risk of fractures [1369,1373,1374]. In a meta-analysis, the RR of fractures was 1.34 for benzodiazepines, 1.60 for antidepressants, 1.54 for anticonvulsants, and 1.59 for antipsychotics [1373]. In a prospective cohort study (The Rotterdam Study) of subjects over 55 years of age, the risk of non-vertebral fractures was 2.35 for current SSRI use versus non-use [1375]. The increased risk for hip fracture associated with benzodiazepines was further increased with increasing dose and the use of concomitant interacting drugs [1369,1374]. There does not appear to be any difference between atypical antipsychotic agents in the increased risk of falls or fractures [1376].

An increased mortality risk has been associated with the use antipsychotics in older patients with dementia [1377-1379], which appears to be greater with conventional compared to atypical antipsychotics [1378-1380].

Antidepressants are frequently used to treat symptoms of anxiety in older adults who suffer from comorbid medical conditions such as heart disease. In a meta-analysis of SSRIs versus placebo or no antidepressant therapy in patients with coronary heart disease (CHD) and depression, SSRIs were associated with lower rates of all-cause mortality and readmissions for CHD, indicating that treatment may improve CHD prognosis [1381]. Clinicians should weigh the risks associated with antidepressants against the potential benefits when making prescribing decisions.

#### Summary

While onset of anxiety and related disorders in late-life is uncommon, they do persist into older age and can have substantial impact on QoL and functionality. Older patients can present differently compared to younger patients, and diagnosis can be complicated by communication barriers, changes in role functioning, memory difficulties, and comorbid medical conditions.

Few treatment studies are conducted in older patients; however, data suggest that psychological treatment and pharmacotherapy appear to be similarly effective in older patients. Using pharmacotherapy in elderly patients can be challenging, and should consider patient factors such as body mass, hepatic and renal function, comorbid conditions, and use of concomitant medications.

## Anxiety with comorbid conditions

### Overview

Anxiety and related disorders often present together with other psychiatric or medical conditions [3,16,43,1382,1383]. About 60-80% of patients with an anxiety disorder have at least one other comorbid psychiatric condition, which most commonly include another anxiety or related disorder, MDD, bipolar disorder, ADHD, and SUD [3]. The presence of comorbid disorders has a negative impact on most aspects of care. Patients with psychiatric comorbidities have more severe symptoms [46,1384], poorer treatment outcomes for both disorders [47,1385-1387], greater functional impairment [46,871,1384], poorer QoL [1388,1389], and an increased risk of suicide [652].

Medical conditions and pain disorders are also common comorbidities in patients with anxiety and related disorders. Medical conditions frequently reported in patients with anxiety and related disorders include cardiovascular disease, gastrointestinal disease, arthritis, respiratory disease, thyroid disease, migraine, and allergic conditions [16,52]. Patients with both anxiety disorders and medical conditions experience elevated disability, including more psychiatric comorbidity and depressive symptoms, as well as poorer interpersonal and physical functioning [52,142]. Patients with chronically painful conditions such as arthritis, back pain, or migraine are at a two- to four-fold higher risk of having an anxiety or related disorder, particularly panic disorder or PTSD [1390].

The high probability of comorbid disorders should be considered when diagnosing and treating patients with anxiety and related disorders. In patients with comorbid psychiatric conditions, such as another anxiety disorder or mood disorder, consider therapies that are effective for both disorders [32]. Benzodiazepines should be prescribed with additional caution in patients with comorbid SUDs. In patients with comorbid medical conditions, the clinician must weigh the benefits and risks of medication for the anxiety or related disorder, but should also consider the impact of untreated anxiety [32].

### Major depressive disorder (MDD)

#### Q. What is the prevalence and impact of comorbid MDD and anxiety/related disorders?

MDD is very common in patients with anxiety, being reported in 20-36% of patients [121,310,360,1382]; and conversely, about 60% of patients with MDD will have a comorbid anxiety or related disorder [44]. In patients with anxiety, comorbid depression has been associated with more severe symptoms [46,1384], lower likelihood of remission [47], greater functional impairment [46,871,1384], an increased risk of suicide [652], and a greater risk of having another comorbid anxiety disorder [360]. Similarly, in patients with MDD, comorbid anxiety and related disorders were associated with poorer treatment outcomes including higher recurrence rates [1385-1387], poorer QoL [1391], and an increased risk of suicide [24,1387,1392,1393].

#### Q. What pharmacological treatment may be useful for patients with an anxiety/related disorder and comorbid MDD?

Guidelines generally recommend antidepressants (most commonly SSRIs and SNRIs) as first-line treatments in patients with both anxiety and depressive symptoms [32,1394]. SSRIs and SNRIs in patients with anxiety and related disorders, including panic disorder, GAD, OCD, or PTSD, with comorbid MDD have been shown to be effective in improving both disorders [224,723,1359,1395]. Among the atypical antipsychotics, quetiapine has been found to have efficacy as monotherapy in both MDD [1396] and GAD [1397], as well as MDD with anxiety [1398], while case series suggest that aripiprazole augmentation of antidepressants [496], and risperidone monotherapy [267] may also reduce comorbid depressive and anxiety symptoms.

### Bipolar disorder or psychoses

#### Q. What is the prevalence and impact of comorbid bipolar disorder or psychoses with anxiety/related disorders?

Among patients with anxiety and related disorders, almost 14% also met criteria for bipolar I or II disorder [121]. However, among patients with bipolar disorder the rates of comorbid anxiety disorders are very high compared to the general population, and the DSM-5 notes anxiety disorders as the most common comorbidities in patients with bipolar disorder [26]. In epidemiological surveys, the lifetime comorbidity rates for any anxiety or related disorder among patients with bipolar disorder was 52% in Canada [43] and 60-75% in the US [1389,1399]. In a clinic population, the rate of anxiety and related disorders was 22% in patients with bipolar disorder, compared to 17% in patients with schizophrenia, and 30% in those with schizoaffective disorder [1400]. A meta-analysis of prevalence studies found that the rates of various anxiety disorders in patients with schizophrenia and related psychotic disorders ranged from 10-15% [1401].

Comorbid anxiety and related disorders in patients with bipolar disorder were associated with a greater risk of MDD and drug use disorders, a poorer bipolar course, lower QoL, and lower psychosocial functioning [1388,1389]. Data are conflicting on the impact of anxiety and related disorders on suicidal tendencies in patients with bipolar disorder, with some analyses finding an increased risk [1389,1402], but not all [1403]. Similar findings have been reported in patients with schizophrenia, where comorbid anxiety and related disorders have been associated with more past SUDs, lower social adjustment and overall QoL, and greater suicidality [1404,1405].

#### Q. What pharmacological treatment may be useful for patients with an anxiety/related disorder and comorbid bipolar disorder or psychoses?

The management of patients with anxiety and related disorders and comorbid bipolar disorder, schizophrenia, or other psychosis should consider therapies that are effective for both disorders [32]. Atypical antipsychotics are recommended treatments for bipolar disorder and schizophrenia [111,1406], while the long-term use of antidepressants may destabilize patients with bipolar I disorder [111,1394].

Data in patients with a diagnosed anxiety or related disorder and comorbid bipolar disorder or psychosis are limited. In a RCT, risperidone monotherapy was shown to be no more effective than placebo for patients with bipolar and comorbid panic disorder or GAD [1407]. However, in a single-blind trial, olanzapine or lamotrigine when added to lithium demonstrated improvements in anxiety disorder symptoms in patients with remitted bipolar disorder [1408]; and in an open trial, switching to aripiprazole significantly improved social anxiety and psychosis in patients with SAD and schizophrenia [379]. In addition, atypical antipsychotics have demonstrated efficacy in RCTs in patients with anxiety and related disorders (see specific disorder sections for evidence), and data show that these agents can significantly reduce anxiety symptoms in patients with bipolar disorder [1409-1413]. Taken together, these data suggest these agents may be useful in comorbid patients.

Anticonvulsants have also demonstrated efficacy in the treatment of some anxiety and related disorders (see specific disorder sections for evidence) and are often used for the treatment of bipolar disorder [111]. In patients with bipolar disorder, adjunctive valproate and gabapentin have demonstrated efficacy for the treatment of panic disorder [281,1414] and resulted in reductions in anxiety symptoms [1415,1416].

### ADHD

#### Q. What is the prevalence of comorbid ADHD and anxiety/related disorders?

It is estimated that the lifetime rate of ADHD in children is 6-9%, with 70% persistence into adolescence and 50-60% into adulthood [45,1417,1418]. In a community-based survey, the estimated prevalence of current self-reported adult ADHD was 4.4% [45]. While ADHD has long been known to persist into adulthood [1419,1420], it has only recently become the focus of widespread clinical attention [1421-1423].

Of adults identified with ADHD in the National Comorbidity Survey-Replication (NCS-R), only one in 10 had received treatment within the previous year [45]. Of these individuals, it is estimated that approximately 47% meet criteria for an anxiety or related disorder within 12 months of assessment, with the most common being SAD (29.3%), followed by specific phobia (22.7%), PTSD (11.9%), panic disorder (8.9%), and GAD (8.0%) [45]. Patients with an anxiety or related disorder were reportedly four times more likely to meet criteria for ADHD than the general population [45]. Similar results were found in a Canadian survey of patients in an anxiety disorders clinic, where the rate of adult ADHD was 28% [378].

#### Q. What factors should be considered when treating patients with an anxiety/related disorder and comorbid ADHD?

When managing a patient with ADHD, it may be important to differentiate ADHD with anxious symptoms from comorbid ADHD and anxiety/related disorders. This can be challenging, as anxiety symptoms are frequently related to a sense of being overwhelmed or to compensatory skills in patients with ADHD. Stimulants may play a larger role in managing ADHD in patients with anxiety symptoms [1424,1425]; however, in an open trial, atomoxetine improved ADHD and comorbid symptoms of depression and anxiety [1426].

Treatment of patients with comorbid ADHD and an anxiety or related disorder may be more complicated. Generally, in patients with comorbid anxiety disorders and ADHD the diagnostic and treatment priority should be determined by the relative severity of symptoms and risks of each disorder [1427]. There are limited data on the role of stimulants in patients with ADHD and an anxiety disorder. In a RCT, atomoxetine significantly improved ADHD and SAD symptoms compared with placebo [487]. In separate open trials, adjunctive atomoxetine [1428] and adjunctive extended release mixed amphetamine salts [1429] significantly improved anxiety symptoms in patients with ADHD and GAD refractory to antidepressants alone.

### Medical comorbidities

#### Q. What is the prevalence and impact of comorbid medical conditions and anxiety/related disorders?

Medical conditions are also common comorbidities that must be considered when prescribing medication for patients with anxiety and related disorders. Medical conditions are reported in over 60% of patients with anxiety and related disorders including cardiovascular diseases, gastrointestinal diseases, arthritis, respiratory diseases such as asthma, thyroid disease, migraine headaches, back pain, and allergic conditions [16,52,1430-1432]. Comorbidities are particularly common among patients with GAD, panic disorder, and PTSD [16,140,515,517,1390,1433].

Patients with anxiety and related disorders and medical conditions experience more psychiatric comorbidity, depressive symptoms, and more severe anxiety disorder symptoms, as well as poorer interpersonal and physical functioning [52,140,142,515].

#### Q. What factors should be considered when treating patients with an anxiety/related disorder and comorbid chronic pain?

Chronically painful conditions (i.e., arthritis, back pain, and migraine) are commonly associated with anxiety [515,1390,1430,1434]. Patients with anxiety and related disorders are twice as likely to have painful physical symptoms compared to of those without, 45-60% versus 28% [515,1433]. About 60-70% of patients with anxiety disorders report migraine headaches [140,141].

For the management of anxiety and related disorders in patients with pain it may be helpful to consider treatments that have demonstrated efficacy in both anxiety disorders as well as pain. While there are few data available, duloxetine has demonstrated efficacy for both GAD and pain symptoms in RCTs [1435-1437]. TCAs, and to a lesser extent SSRIs, have been shown to reduce headache attacks in patients with migraine [1438], and provide moderate relief of neuropathic pain [1439].

#### Q. What factors should be considered when treating patients with an anxiety/related disorder and comorbid cardiovascular disease?

Although panic attacks can sometimes be mistaken for cardiovascular symptoms, it is important to be aware that patients with anxiety and related disorders do have a two- to three-times greater risk of cardiovascular disease compared to the general population [1431,1432]. In addition, anxiety disorders have been associated with increased risk of cardiovascular hospitalization rates and mortality risk [1440-1442]. In patients with cardiovascular or cerebrovascular comorbidity, it is important to consider the impact of treatments used for anxiety on heart rate, blood pressure, and lipid measures [1443-1445].

#### Q. What factors should be considered when treating patients with an anxiety/related disorder and comorbid diabetes and metabolic syndrome?

Patients with anxiety symptoms have an elevated risk of type 2 diabetes [1446]. While glycemic measures do not appear to be affected by anxiety symptoms [1447], some treatments, particularly some atypical antipsychotics, alter glucose parameters, lipid levels, and cause weight gain [109-116,1443]. Some antidepressants, including amitriptyline, mirtazapine, and paroxetine have also been associated with weight gain [1448].

## Canadian Anxiety Guidelines Initiative Group

### Additional authors

Martin M. Antony^1^, Stéphane Bouchard^2^, Alain Brunet^3^, Martine Flament^4^, Sophie Grigoriadis^5^, Sandra Mendlowitz^6^, Kieron O’Connor^7^, Kiran Rabheru^4^, Peggy M.A. Richter^5^, Melisa Robichaud^8^, John R. Walker^9^

^1^Department of Psychology, Ryerson University, Toronto, M5B 2K3, Canada; ^2^Department of Psychoeducation and Psychology, University of Québec in Outaouais, Gatineau, J9A 1L8, Canada; ^3^Department of Psychiatry, McGill University, Montreal, H3A 1A1, Canada; ^4^Department of Psychiatry, University of Ottawa, Ottawa, K1Z 7K4, Canada; ^5^Department of Psychiatry, University of Toronto, Toronto, M5S 1A1, Canada; ^6^Department of Child Psychiatry, University of Toronto, Toronto, M5S 1A1, Canada; ^7^Department of Psychiatry, University of Montreal, Montreal, H3C 3J7, Canada; ^8^Departments of Psychiatry and Psychology, University of British Columbia, Vancouver, V6T 2A1, Canada; ^9^Department of Clinical Health Psychology, University of Manitoba, Winnipeg, R3E 3N4, Canada

**Email:** Martin M. Antony - mantony@psych.ryerson.ca; Stéphane Bouchard - stephane.bouchard@uqo.ca; Alain Brunet - alain.brunet@mcgill.ca; Martine Flament - martine.flament@theroyal.ca; Sophie Grigoriadis - sophie.grigoriaidis@sunnybrook.ca; Sandra Mendlowitz - sandra.mendlowitz@sickkids.on.ca; Kieron O’Connor - kieron.oconnor@umontreal.ca; Kiran Rabheru - kiranrabheru@hotmail.com; Peggy M.A. Richter - peggy.richter@sunnybrook.ca; Melisa Robichaud - robichau@mail.ubc.ca; John R. Walker - jwalker@cc.umanitoba.ca

### Additional contributors to the comorbidity section

Gordon Asmundson^10^, Larry J. Klassen^11^, Raymond W. Lam^12^, Roger S. McIntyre^13^, Isaac Szpindel^14^

^10^Department of Psychology, University of Regina, Regina, S4S 0A2, Canada; ^11^Department of Psychiatry, Faculty of Medicine, University of Manitoba, Winnipeg, R3T 2N2, Canada; ^12^Department of Psychiatry, University of British Columbia, Vancouver, V6T 2A1, Canada; ^13^Departments of Psychiatry and Pharmacology, University of Toronto, Toronto, M5S 1A1, Canada; ^14^Attention and Learning Related Disorders, START Clinic, Toronto, M4W 2N4, Canada

**Email:** Gordon Asmundson - gordon.asmundson@uregina.ca; Larry J. Klassen - larryjklassen@hotmail.com; Raymond W. Lam - r.lam@ubc.ca; Roger S. McIntyre - roger.mcintyre@uhn.ca; Isaac Szpindel - iszpindel@startclinic.ca

## List of abbreviations used

AACAP: American Academy of Child and Adolescent Psychiatry; ABM: attention bias modification; ACOG: American Congress of Obstetricians and Gynecologists; ADHD: attention-deficit/hyperactivity disorder; APA: American Psychiatric Association; ASD: acute stress disorder; B-I-I: blood-injection-injury; BPD: borderline personality disorder; CBT: cognitive behavioral therapy; CBWT: cognitive behavioral writing therapy; CCHS: Canadian Community Health Survey; CHD: coronary heart disease; CPT: cognitive processing therapy; CR: controlled release; DBT: dialectical behavioral therapy; DDI: drug-drug interactions; DIRT: danger ideation reduction therapy; DSM-5: Diagnostic and Statistical Manual of Mental Disorders, 5^th^ Edition; EMDR: eye movement desensitization and reprocessing; ERP: exposure with response prevention; FDA: Food and Drug Administration; GAD: generalized anxiety disorder; HARS: Hamilton Anxiety Rating Scale; HDL: high-density lipoprotein; ICBT: internet-based CBT; IPT: interpersonal therapy; IV: intravenous; LDL: low-density lipoprotein; MAOI: monoamine oxidase inhibitor; MBCT: mindfulness-based cognitive therapy; MBT: mindfulness-based therapy; MDD: major depressive disorder; Mini-SPIN: Mini-Social Phobia Inventory; MRI: magnetic resonance imaging; N/A: not available; NaSSA: noradrenergic and specific serotonergic antidepressant; NCS-A: National Comorbidity Survey – Adolescent supplement; NCS-R: National Comorbidity Survey – Replication; NMDA: *N*-methyl-D-aspartate; NNT: number needed to treat; NPPO-REAC: neuro psycho physical optimization-radio electric asymmetric conveyor; NSAID: nonsteroidal anti-inflammatory drug; OCD: obsessive-compulsive disorder; ODT: orally disintegrating tablet; PNAS: poor neonatal adaptation syndrome; PTSD: posttraumatic stress disorder; QoL: quality of life; RCT: randomized controlled trial; REAC: radioelectric asymmetric conveyor; RIMA: reversible inhibitors of monoamine oxidase A; RR: relative risk; rTMS: repetitive transcranial magnetic stimulation; SAD: social anxiety disorder; SET: social effectiveness therapy; SHAT: spiritual-hypnosis assisted therapy; SNRI: serotonin–norepinephrine reuptake inhibitor; SR: sustained release; SSRI: selective serotonin reuptake inhibitor; SUD: substance use disorder; TC: total cholesterol; TCA: tricyclic antidepressant; TF-CBT: trauma-focused-CBT; TG: triglycerides; vLDL: very-low-density lipoprotein; VRE: virtual reality exposure; XL: extended release; XR: extended release; Y-BOCS: Yale-Brown Obsessive Compulsive Scale.

## Competing interests

Unrestricted educational grants for the development of these guidelines were provided by Astra Zeneca Canada, Eli-Lilly Canada, Janssen Inc., Lundbeck Canada, Pfizer Canada, Purdue Canada, Servier Canada Inc., Shire Canada, and Valeant Canada. None of the members received payment for participating in the development of these guidelines.

**The following authors do not have any competing interests to declare:** AB, GA, JRW, KO, MMA, MF, LK, MR, SM.

**Advisory board/speaker's bureau:** Astra Zeneca (KK, KR, MK, P. Bleau, P. Blier, RWL, RSM), Biovail (RWL), Boehringer Ingelheim (MK), BMS (KK, KR, MK, P. Bleau, P. Blier, PC, RWL, RSM), Canadian Institutes of Health Research (CIHR) (RWL), Canadian Network for Mood and Anxiety Treatments (CANMAT) (RWL), Canadian Psychiatric Association (CPA) Foundation (RWL), Eli Lilly Canada (MK, P. Bleau, P. Blier, PC, SG, RWL, RSM, IS), France Foundation (RSM), GlaxoSmithKline (MK, P. Blier, RSM, SG), Janssen Ortho (KK, MK, P. Blier, PC, RSM), Labopharm (P. Blier), Litebook Company (RWL), Lundbeck (KK, KR, MK, P. Bleau, P. Blier, PC, RWL, RSM, SG), Lundbeck Institute (RWL), Organon (MK, RSM), Merck (P. Blier, RSM), Mochida (RWL), Otsuka (KK), Pfizer (P. Bleau, P. Blier, PC, RWL, RSM, SG), Pierre Fabre (P. Blier), Purdue (IS), Servier (P. Blier, RWL, SG), Shire (MK, RSM, IS), Solvay (MK), St. Jude's Medical (RWL), Sunovion (KK, P. Blier), Takeda (P. Blier, RWL), UBC Institute of Mental Health/Coast Capital Savings (RWL), Valeant (P. Blier, IS), Wyeth (P. Bleau, MK, SG)

**Consultation fees:** AstraZeneca (MK), BMS (MK), Boehringer Ingelheim (MK), Clinique et Développement In Virtuo Inc. (SB), Eli Lilly Canada (MK, SG), GlaxoSmithKline (MK, SG), Janssen Ortho (MK), Lundbeck (MK, PR, SG), Organon (MK), Pfizer (SG), Servier (SG), Shire (MK), Solvay (MK), Wyeth (MK, SG)

**Educational support:** Astra Zeneca (RSM), BMS (RSM), CME Outfitters (RSM), Eli Lilly Canada (RSM, IS), France Foundation (RSM), I3CME (RSM), Merck (RSM), Optum Health (RSM), Pfizer (RSM), Physicians' Postgraduate Press (RSM), Shire (IS)

**Research grants/clinical trial funding:** AstraZeneca (KK, MK, P. Bleau, RSM, RWL), Biovail (RWL), BMS (KK, P. Bleau, RWL), Canadian Foundation for Innovation (MK), Canadian Institutes of Health Research (CIHR) (MK, RWL, SG), Canadian Network for Mood and Anxiety Treatments (CANMAT) (RWL), Canadian Psychiatric Association (CPA) Foundation (MK, RWL), Centre for Addiction and Mental Health Foundation (MK), CR Younger Foundation (SG), Eli Lilly Canada (MK, P. Bleau, PR, RSM, RWL), Genuine Health (MK), GlaxoSmithKline (MK), Janssen Ortho (MK, RSM), Litebook Company (RWL), Lundbeck (KK, MK, P. Bleau, RSM, RWL), Lundbeck Institute (RWL), Mochida (RWL), National Alliance for Research on Schizophrenia and Depression (NARSAD) (RSM), National Institutes of Mental Health (NIMH) (RSM), Ontario Ministry of Health (SG), Ontario Mental Health Foundation (SG), Organon (MK), Pfizer (P. Bleau, RSM, RWL), Roche (PR), Servier (RWL), Sick Kids Foundation (MK), Solvay (MK), St. Jude's Medical (RWL), Shire (MK, RSM), Stanley Medical Research Institute (RSM), Takeda (RWL), UBC Institute of Mental Health/Coast Capital Savings (RWL), Wyeth (MK, P. Bleau)

**Unrestricted grants**: Astra Zeneca Canada (MK), Eli Lilly Canada (MK), Janssen Inc. (MK), Lundbeck Canada (MK), Pfizer (MK), Purdue Pharma (MK), Servier Canada (MK), Shire Canada (MK), Valeant Canada (MK)

**Reimbursements, fees, funding, or salary:** In the past five years, MVA received reimbursements, fees, funding, or salary from: Astra Zeneca, Biovail, Canadian Foundation for Innovation (CFI), Cephalon, Eli Lilly, Forest Laboratories, GlaxoSmithKline, Hamilton Academic Health Sciences Organization (HAHSO) Innovation Grant (AFP Innovation Grant), Janssen Ortho, Labo Pharm, Lundbeck, National Institutes of Health (NIH), Novartis, Pfizer Inc., Servier, Shire, Sunovion, Valeant, Wyeth-Ayerst

**Stock/share ownership:** Clinique et Développement In Virtuo Inc. (SB)

## Authors’ contributions

We thank all co-authors for their considerable expertise in generating these guidelines. Authors who were members of the executive committee (MK, PB, PB, PC, KK, MVA) took part in teleconferences and a meeting in December 2012 to reach consensus on the strength of evidence and treatment recommendations. Draft guidelines were then developed by the core committee and revised by all co-authors. The entire content was subsequently circulated to all members of the Canadian Anxiety Guidelines Initiative Group for additional comments and approval during 2013. GA, LJK, RWL, RSM, and IS provided additional reviews of the comorbidity section. The final manuscript was then circulated to external reviewers (MP, DS, LDM) and revisions were made based on input from the core committee.

## Supplementary Material

Additional file 1 **Suggested dosing ranges** Dosing ranges of various psychiatric medications Click here for file

## Declarations

The development and publication of these guidelines was supported by unrestricted educational grants provided by Astra Zeneca Canada, Eli-Lilly Canada, Janssen Inc., Lundbeck Canada, Pfizer Canada, Purdue Canada, Servier Canada Inc., Shire Canada, and Valeant Canada. None of the members received payment for participating in the development of these guidelines.

This article has been published as part of *BMC Psychiatry* Volume 14 Supplement 1, 2014: Canadian Anxiety Disorders Guidelines Initiative: Clinical practice guidelines for the management of anxiety, posttraumatic stress and obsessive-compulsive disorders. The full contents of the supplement are available online at http://www.biomedcentral.com/bmcpsychiatry/supplements/14/S1.
