# Supplementary material for: Canadian clinical practice guidelines for the management of anxiety, posttraumatic stress and obsessive-compulsive disorders
Source: BMC Psychiatry. 2014 Jul 2;14(Suppl 1):S1. doi: 10.1186/1471-244X-14-S1-S1 (PMC4120194; doi:10.1186/1471-244X-14-S1-S1)
Supplement: Additional file 1 — Suggested dosing ranges Dosing ranges of various psychiatric medications [file 1471-244X-14-S1-S1-S1.doc]

*Additional file 1: Suggested dosing ranges*

Note: All ranges are for drugs if used as a monotherapy unless otherwise specified.

PANIC DISORDER

| **SSRIs** | **Dose range (mg/day)** |
| --- | --- |
| Citalopram | 10 - 40 |
| Escitalopram | 10 - 20 |
| Fluoxetine | 10 - 80 |
| Fluvoxamine | 50 - 300 |
| Paroxetine | 10 - 60 |
| Paroxetine CR | 12.5 - 75 |
| Sertraline | 50 - 200 |
| **SNRIs** | **Dose range (mg/day)** |
| Venlafaxine XR | 75 - 300 |
| Duloxetine | 30 – 120 |
| Milnacipran | 50 – 100 |
| **TCAs** | **Dose range (mg/day)** |
| Clomipramine | 50 – 250 |
| Imipramine | 50 – 300 |
| **MAOIs and RIMAs** | **Dose range (mg/day)** |
| Tranylcypromine | 20 – 60 |
| Phenelzine | 15 – 60 |
| Moclobemide | 150 – 600 |
| **Other antidepressants** | **Dose range (mg/day)** |
| Bupropion SR | 50 – 300 |
| Mirtazapine | 15 – 60 |
| **Benzodiazepines** | **Dose range (mg/day)** |
| Alprazolam* | 1 – 8 |
| Clonazepam* | 1 – 4 |
| Diazepam | 2 – 40 |
| Lorazepam | 1 – 8 |
| **Atypical antipsychotics** | **Dose range (mg/day)** |
| Risperidone* | 0.5 – 5 |
| Quetiapine XR | 50 – 300 |
| Olanzapine* | 2.5 – 20 |
| **Anticonvulsants** | **Dose range (mg/day)** |
| Gabapentin | 300 - 3600 |

CR = controlled release; MAOI = monoamine oxidase inhibitor; RIMA = reversible inhibitor of monoamine oxidase A; SNRI = serotonin–norepinephrine reuptake inhibitor; SR = sustained release; SSRI = selective serotonin reuptake inhibitor; TCA = tricyclic antidepressant; XR = extended release

*Monotherapy and augmentation

^†^Augmentation only, usually added to an antidepressant medication

GENERALIZED ANXIETY DISORDER

| **SSRIs** | **Dose range (mg/day)** |
| --- | --- |
| Citalopram | 10 – 40 |
| Escitalopram | 10 – 20 |
| Fluoxetine | 10 – 80 |
| Fluvoxamine | 50 – 300 |
| Paroxetine | 10 – 60 |
| Paroxetine CR | 12.5 – 75 |
| Sertraline | 50 – 200 |
| **SNRIs** | **Dose range (mg/day)** |
| Venlafaxine XR | 75 – 300 |
| Duloxetine | 30 – 120 |
| **TCAs** | **Dose range (mg/day)** |
| Imipramine | 50 – 300 |
| **Other antidepressants** | **Dose range (mg/day)** |
| Agomelatine | 25 – 50 |
| Bupropion XL | 150 – 300 |
| Buspirone | 10 – 60 |
| Vortioxetine | 5 – 10 |
| Mirtazapine | 30 – 60 |
| **Benzodiazepines** | **Dose range (mg/day)** |
| Alprazolam | 1 – 4 |
| Bromazepam | 6 – 30 |
| Clonazepam | 1 – 4 |
| Diazepam | 2 – 40 |
| Lorazepam | 1 – 8 |
| **Atypical antipsychotics** | **Dose range (mg/day)** |
| Aripiprazole^†^ | 2 – 30 |
| Quetiapine^†^ | 25 – 300 |
| Quetiapine XR* | 50 – 300 |
| Risperidone^†^ | 0.5 – 5 |
| **Anticonvulsants** | **Dose range (mg/day)** |
| Gabapentin | 300 - 3600 |
| Pregabalin* | 75 – 600 |
| **Other therapies** | **Dose range (mg/day)** |
| Hydroxyzine | 25 – 100 |
| Opipramol | 50 - 150 |

CR = controlled release; SNRI = serotonin–norepinephrine reuptake inhibitor; SSRI = selective serotonin reuptake inhibitor; TCA = tricyclic antidepressant; XL = extended release; XR = extended release

*Monotherapy and augmentation

^†^Augmentation only, usually added to an antidepressant medication

**SOCIAL ANXIETY DISORDER**

| **SSRI** | **Dose range (mg/day)** |
| --- | --- |
| Citalopram | 10 - 40 |
| Escitalopram | 10 - 20 |
| Fluoxetine | 10 - 80 |
| Fluvoxamine IR | 50 - 300 |
| Fluvoxamine CR | 100 - 300 |
| ^‡^Paroxetine IR* | 10 - 60 |
| Paroxetine CR | 12.5 - 75 |
| Sertraline | 50 - 200 |
| **SNRIs** | **Dose range (mg/day)** |
| Venlafaxine XR | 75 - 300 |
| Duloxetine | 30 - 120 |
| **TCAs** | **Dose range (mg/day)** |
| Clomipramine | 50 - 250 |
| **MAOIs and RIMAs** | **Dose range (mg/day)** |
| Moclobemide | 150 - 600 |
| Phenelzine* | 30 - 90 |
| **Other antidepressants** | **Dose range (mg/day)** |
| Bupropion SR | 50 - 300 |
| Buspirone^†^ | 10 - 60 |
| Mirtazapine | 15 - 60 |
| **Benzodiazepines** | **Dose range (mg/day)** |
| Alprazolam | 1 - 8 |
| Bromazepam | 6 - 30 |
| Clonazepam | 1 - 4 |
| Diazepam | 2 - 20 |
| Lorazepam | 1 - 8 |
| **Atypical antipsychotics** | **Dose range (mg/day)** |
| Olanzapine | 2.5 - 20 |
| Quetiapine | 25 - 300 |
| Aripiprazole^†^ | 2 - 30 |
| Risperidone^†^ | 0.5 - 5 |
| **Anticonvulsants** | **Dose range (mg/day)** |
| Gabapentin | 300 - 3600 |
| Pregabalin | 75 - 600 |
| Topiramate | 50 - 400 |

CR = controlled release; IR = immediate release; MAOI = monoamine oxidase inhibitor; RIMA = reversible inhibitor of monoamine oxidase A; SNRI = serotonin–norepinephrine reuptake inhibitor; SR = sustained release; SSRI = selective serotonin reuptake inhibitor; TCA = tricyclic antidepressant; XR = extended release

*Monotherapy and augmentation

^†^Augmentation only, usually added to an antidepressant medication

^‡^Used as monotherapy and in one study as an augmentation agent to mirtazapine

**OBSESSIVE-COMPULSIVE DISORDER**

| **SSRIs** | **Dose range (mg/day)** |
| --- | --- |
| Citalopram | 20 - 40 |
| IV Citalopram | 20 - 80 |
| Escitalopram | 10 - 20 |
| Fluoxetine* | 20 - 80 |
| Fluvoxamine | 50 - 300 |
| Paroxetine | 20 - 60 |
| Sertraline | 50 - 200 |
| **SNRIs** | **Dose range (mg/day)** |
| Venlafaxine XR | 75 - 300 |
| Duloxetine | 60 - 120 |
| **TCAs** | **Dose range (mg/day)** |
| ^§^Clomipramine* | 50 - 300 |
| **MAOIs and RIMAs** | **Dose range (mg/day)** |
| Phenelzine | 30 - 90 |
| **Other antidepressants** | **Dose range (mg/day)** |
| Mirtazapine* | 30 - 60 |
| **Atypical antipsychotics** | **Dose range (mg/day)** |
| Quetiapine^†^ | 50 - 600 |
| Olanzapine^†^ | 2.5 - 20 |
| Risperidone^†^ | 0.5 - 6 |
| Aripiprazole^†^ | 2 - 15 |
| **Anticonvulsants** | **Dose range (mg/day)** |
| Lamotrigine^†^ | 50 - 200 |
| Pregabalin^†^ | 75 - 600 |
| Topiramate^†^ | 50 - 400 |

IV = intravenous; MAOI = monoamine oxidase inhibitor; RIMA = reversible inhibitor of monoamine oxidase A; SNRI = serotonin–norepinephrine reuptake inhibitor; SSRI = selective serotonin reuptake inhibitor; TCA = tricyclic antidepressant; XR = extended release

*Monotherapy and augmentation

^†^Augmentation only, usually added to an antidepressant medication

^§^Two studies: combination of exposure therapy and clomipramine; another an augmentation study adding clomipramine to fluoxetine

**POSTTRAUMATIC STRESS DISORDER**

| **SSRIs** | **Dose range (mg/day)** |
| --- | --- |
| Escitalopram | 10 - 20 |
| Fluoxetine | 10 - 80 |
| Fluvoxamine | 50 - 300 |
| Paroxetine | 10 - 60 |
| Sertraline | 50 - 200 |
| **SNRIs** | **Dose range (mg/day)** |
| Venlafaxine XR | 75 - 300 |
| Duloxetine | 30 - 120 |
| **TCAs** | **Dose range (mg/day)** |
| Imipramine | 50 - 300 |
| **MAOIs and RIMAs** | **Dose range (mg/day)** |
| Moclobemide | 150 - 600 |
| Phenelzine | 30 - 90 |
| **Other antidepressants** | **Dose range (mg/day)** |
| Mirtazapine | 15 - 60 |
| Burpropion SR^†^ | 50 - 300 |
| **Atypical antipsychotics** | **Dose range (mg/day)** |
| Risperidone* | 0.5 - 8 |
| Aripiprazole* | 2 - 30 |
| Quetiapine* | 25 - 600 |
| Olanzapine^†^ | 2.5 - 20 |
| **Anticonvulsants** | **Dose range (mg/day)** |
| Lamotrigine | 50 - 200 |
| Pregabalin^†^ | 75 - 600 |
| Topiramate | 50 - 400 |

MAOI = monoamine oxidase inhibitor; RIMA = reversible inhibitor of monoamine oxidase A; SNRI = serotonin–norepinephrine reuptake inhibitor; SR = sustained release; SSRI = selective serotonin reuptake inhibitor; TCA = tricyclic antidepressant; XR = extended release

*Monotherapy and augmentation

^†^Augmentation only, usually added to an antidepressant medication
